# Interventions to improve hand hygiene in community settings: a systematic review of theories, barriers and enablers, behaviour change techniques and hand hygiene station design features

**DOI:** 10.1136/bmjgh-2025-018928

**Published:** 2025-09-16

**Authors:** Sridevi K Prasad, Jedidiah S Snyder, Erin LaFon, Lilly A O’Brien, Hannah K Rogers, Oliver Cumming, Joanna Esteves Mills, Bruce Gordon, Marlene K Wolfe, Matthew C Freeman, Bethany A Caruso

**Affiliations:** 1 Hubert Department of Global Health, Rollins School of Public Health, Emory University, Atlanta, Georgia, USA; 2 Gangarosa Department of Environmental Health, Rollins School of Public Health, Emory University, Atlanta, Georgia, USA; 3 Woodruff Health Sciences Center Library, Emory University, Atlanta, Georgia, USA; 4 Department of Disease Control, London School of Hygiene & Tropical Medicine, London, UK; 5 Water, Sanitation, Hygiene and Health Unit, World Health Organization, Geneva, Switzerland

**Keywords:** Hygiene, Systematic review, Prevention strategies, Health education and promotion

## Abstract

**Introduction:**

This systematic review identified and examined the theories, barriers and enablers, behaviour change techniques (BCTs), and design features of interventions that have been leveraged to improve and sustain hand hygiene in community settings. It was conducted to support the development of the WHO Guidelines for Hand Hygiene in Community Settings.

**Methods:**

We searched PubMed, Web of Science, EMBASE, CINAHL, Global Health, Cochrane Library, Global Index Medicus, Scopus, PAIS Index, WHO IRIS, UN Digital Library and World Bank eLibrary for studies published through 29 March 2023, and consulted experts. Eligible studies had an intervention targeting hand hygiene behaviour, quantitatively measured hand hygiene practice, were published in English after 1 January 1980 and were set in non-healthcare community settings. Studies in healthcare settings, nursing homes or long-term care facilities were excluded. Two reviewers independently extracted data from each study and appraised study quality (Mixed Method Appraisal Tool).

**Results:**

223 eligible studies (including 247 398 participants) met inclusion criteria, 82% of which were reported to be effective at improving hand hygiene. A minority (28%) used theory to inform intervention design. Interventions did not always address identified barriers or enablers. Most interventions addressed ‘action knowledge’ (eg, handwashing instruction), which was not a widely reported barrier or enabler. Interventions did not extensively address the physical environment (eg, resource availability) despite its importance for hand hygiene. Interventions leveraged a variety of BCT combinations, limiting comparability. We did not conduct a meta-analysis on effectiveness due to heterogeneity across studies. 10 studies evaluated hand hygiene station design adaptation, six examined variations in frequency or intensity of intervention delivery, and four focused on people with disabilities, revealing gaps in evidence.

**Conclusions:**

Findings are limited by inconsistent intervention reporting but more consistent identification and leveraging of barriers and enablers would improve alignment of hand hygiene interventions to local context.

**PROSPERO registration number:**

CRD42023429145.

**WHAT IS ALREADY KNOWN ON THIS TOPIC**

- Hand hygiene can prevent infectious diseases, yet little is known about what interventions have been delivered in community settings and if and how they have leveraged theory and behavioural factors, such as barriers and enablers, to improve hygiene behaviours.

**WHAT THIS STUDY ADDS**

- This systematic review examined hand hygiene interventions across community settings to assess if theory informed design and reported effectiveness, how and if barriers and enablers were leveraged, and to understand what intervention functions, behaviour change techniques and hand hygiene station design features have been tested.
- Most hand hygiene interventions in community settings were reported to have been effective, though they are not comparable because of variability in setting, focal population, outcome tested and intervention strategy.
- Despite their reported effectiveness, interventions did not always address identified barriers or enablers, potentially limiting impact.

**HOW THIS STUDY MIGHT AFFECT RESEARCH PRACTICE OR POLICY**

- Evidence from this review demonstrates the need for greater alignment between identified behavioural barriers/enablers and intervention activities.
- Researchers need to improve how they describe and report on interventions to facilitate understanding of what interventions were trying to do, how and among whom, which can facilitate future learning.
- Policymakers and practitioners should ensure that key physical resources, such as water, soap and hygiene stations, are present before implementing interventions that do not provide materials.

## Introduction

 The COVID-19 pandemic re-prioritised hand hygiene as a critical public health measure.^1^ Poor hand hygiene contributes to several infectious diseases, including enteric^2^ and respiratory^3^ infections, which account for a large burden of disease^4^ and burdensome healthcare costs.^5^ Interventions promoting hand hygiene, whether through handwashing with water and soap or use of other methods such as alcohol-based hand rubs, are relatively inexpensive to implement^6^ and are a key component of infectious disease prevention. Due to the impact of hand hygiene interventions, guidelines focused on child health, sanitation and home water supply have emphasised the importance of investing in hand hygiene as a core public health measure.^17^,^9^ Global guidelines and recommendations are essential to guide hand hygiene initiatives, protect public health and strengthen resilient health systems.^10^ Though guidelines for hand hygiene in healthcare settings have been well-established,^11^,^14^ gaps and inconsistencies remain in global guidance beyond healthcare.^15^

One important gap in global guidance is the lack of guidelines for hand hygiene in community settings. Though community settings are where people spend most of their day^16^ and comprise domestic, institutional and public spaces,^17^ there are no global guidelines for hand hygiene in these areas. A recent scoping review examined 51 existing international guidelines and identified behaviour change, in addition to effective hand hygiene, minimum requirements and government measures as areas where clear recommendations are needed for hand hygiene in community settings.^17^ Guidance on effective interventions to change hand hygiene behaviour in community settings is needed to protect public health. However, current understanding of which hand hygiene interventions in community settings are effective is limited, constraining the creation of such guidance.

While evidence reviews do exist, their scopes are limited. For example, reviews that have synthesised behaviour change theories and techniques used to design hand hygiene interventions are limited to settings with children^18 19^ and evidence synthesised on hand washing station designs or adaptations are limited to tippy taps (a low-cost, easily constructed handwashing station) and nudges (such as visual cues), respectively.^20 21^ Recent reviews that have synthesised measures to promote hand hygiene focus specifically on COVID-19.^22 23^ While these reviews and syntheses are informative, there is a need for a systematic review that identifies the theories, intervention functions, behaviour change techniques (BCTs) and design features that have been effective across all public settings.

The goal of this systematic review was to comprehensively examine the theories, barriers and enablers, intervention functions, BCTs and design features of interventions that have been leveraged to improve and sustain hand hygiene in community settings. The priority question and sub-questions for this review were generated through an extensive consultation process by WHO with external experts,^24 25^ following a scoping review of current international guidelines.^17^

## Methods

### Research questions

This systematic review sought to assess interventions to improve hand hygiene in community settings by answering the following seven questions (Sample, Phenomenon of Interest, Design, Evaluation, Research Type [SPIDER] and Population, Intervention, Comparison, Outcomes, Study Design [PICOS] criteria described in online supplemental file S1): (a) Which have been designed using behaviour change theories? (b) Which have effectively leveraged identified barriers and enablers of hand hygiene in community settings? (c) What BCTs have been implemented to effectively improve and sustain handwashing practices? (d) What hand hygiene station designs have been effective at improving and sustaining hand hygiene? (e) What hand hygiene station design adaptations (eg, placement, nudges and cues) have been effective at improving and sustaining hand hygiene? (f) What level of frequency and intensity of behaviour change interventions is necessary to effectively improve hand hygiene? and (g) How do hand hygiene practices vary by population groups, risk scenarios or over time?

### Search strategy

This review was pre-registered with PROSPERO and is reported following the Preferred Reporting Items for Systematic Reviews and Meta-Analyses^26^ (PRISMA) criteria (see online supplemental file S2—PRISMA checklist). This review was part of an integrated protocol^25^ for five related reviews to synthesise the evidence for effective hand hygiene in community settings.^27^,^30^ We adopted a two-phased approach for identifying relevant studies. Phase 1 involved a broad search of databases to capture all studies on hand hygiene in community settings that were relevant across all five of the related systematic reviews. Phase 2 leveraged the reduced sample identified in phase 1, along with additional studies identified through expert consultations and hand-searches of reference lists, to further screen studies (first by title and abstract, then full-text review) using criteria specific to this review. A full description of the research questions and the procedures followed for searches, study inclusion, outcomes data collection, analysis and reporting of the multiple related reviews is presented in the published protocol.^25^

The Phase 1 search included studies published through 29 March 2023 that were either published in English or had their title and abstract in English. We searched 12 peer-reviewed and grey literature databases. PubMed, Web of Science, EMBASE (Elsevier), CINAHL (EBSCOhost), Global Health (CAB), Cochrane Library, Global Index Medicus, Scopus (Elsevier), Public Affairs Information Service (PAIS) Index (ProQuest) were searched on 23 March 2023 and WHO Institutional Repository for Information Sharing (IRIS), UN Digital Library and World Bank eLibrary were searched on 28 March 2023 using search terms related to hand hygiene broadly and with restrictions on terms related to healthcare settings in the titles. We searched trial registries (International Clinical Trials Registry Platform, clinicaltrials.gov) for trials related to hand hygiene in community settings on 29 March 2023.

The Cochrane Library was searched in order to identify relevant studies in existing reviews. We conducted manual searches of reference lists of four relevant systematic reviews.^18^,^21^ We only searched references for articles that were included in the respective reviews. These reviews included 82 eligible references, of which 46 were duplicates, 17 were already identified in our database search and 19 were added to phase 2 title and abstract screening. We contacted 35 content experts and organisations, using snowballing methods, between April and May 2023 for information on relevant unpublished literature.

### Selection criteria

Eligible study designs were mixed-methods studies, randomised and non-randomised control trials or before-after studies of interventions to improve hand hygiene in community settings.

Hand hygiene is defined as any hand cleansing undertaken for the purpose of removing or deactivating pathogens from hands and efficacious hand hygiene is defined as any practice which effectively removes or deactivates pathogens from hands and thereby has the potential to limit disease transmission.^12^ We define ‘community settings’ as locations where healthcare is not routinely delivered and broadly spans all places where people ‘learn, play, work and love’, including domestic (eg, households), public (eg, markets, public transportation hubs, vulnerable populations [eg, people experiencing homelessness], parks, squares or other public outdoor spaces, shops, restaurants and cafes), and institutional (eg, workplace, schools and universities, places of worship, prisons and places of detention) spaces.^17^ Studies were excluded if they were in healthcare settings or were animal research. Studies focusing on care providers’ hand hygiene in nursing homes, home-based care settings and long-term care facilities were excluded during phase 2 screening as evidence they generated were determined to be similar to that from healthcare settings. There were no geographic restrictions.

We used Covidence software for systematic reviews.^31^ In both phases, screening of each article (phase 1—title and abstract only; phase 2—title and abstract, then full-text review) was performed independently by two reviewers, with discordance between reviewers reconciled by a third reviewer. To identify linked studies, one reviewer screened all included studies and mapped the intervention names, descriptions and trial registration numbers (if applicable). Linked studies were grouped together based on intervention name or trial registration number. If there were multiple linked studies involving the same intervention, we only extracted information from the most recent study that measured hand hygiene practice as a primary outcome. During this mapping process, the studies included were reassessed to confirm they met the eligibility criteria. Studies marked for exclusion were independently assessed by two reviewers to confirm exclusion. The stages and related outcomes of the search and screening process are described in the PRISMA flow chart (online supplemental file S3—PRISMA flow chart).

### Data analysis

Reviewers (SKP, EL) independently extracted data from each included study using a customised data extraction tool and the Mixed Method Appraisal Tool (MMAT) for appraising study quality.^32 33^ For appraising the quality of mixed-methods studies, the individual components were assessed using the appropriate categories: qualitative component, quantitative category for the quantitative component, and mixed-methods category. Final scores were determined based solely on the quantitative components of the MMAT assessment, given that only quantitative data were extracted from the mixed-methods studies included in the review. Any conflicts between reviewers over data extraction and bias assessment were resolved by discussion while re-checking the source. All data extraction templates are provided in the supplement (online supplemental file S4—Covidence extraction sheet; online supplemental file S5—outcome extraction sheet).

To provide a broad overview of included studies, we extracted information about study characteristics, including the region, location, setting, primary research population, primary outcome, hand hygiene practice assessed and intervention categories (defined in table 1).

**Table 1:** Definitions and examples of intervention categories and constructs from the COM-B framework* used to code study interventions.

Intervention category	Definition	Examples
Material provision	Provision of material resources to aid in handwashing	Provision of soap or soapy water Provision of alcohol-based hand rub Handwashing station installation
Education programmes†	Curriculum-based teaching programmes, where instruction is delivered using lectures	School-based education Food hygiene education Education—adults
Training programmes†	Demonstration-based teaching programmes, where instruction is delivered using interactive activities (eg, Glo-Germ demonstrations, food preparation demonstrations, etc)	School-based training Food hygiene training Training—adults
Multimedia messaging†	Media-based communication and messages used to improve handwashing	Posters Videos SMS Radio messages Art installations/flipcharts Pamphlets Multiple media types
Hygiene promotion	Community-based programmes to promote handwashing that encompass different interactive activities	Games or competitions Plays, skits, songs or dramas Group discussions Policy development or institutional strengthening Public pledging ceremonies Household visits from community health workers Diaries and journals Provision of incentives (gift cards, face masks, creams, etc)

COM-B construct	Definition‡	Examples
Capability	Knowledge, skills and abilities required to engage in a particular behaviour	
Physical capability	Physical strength, skill or stamina	Physical ability to wash hands
Psychological capability	Knowledge/psychological strength, skills or stamina	Knowledge on how to wash hands
Opportunity	External factors which make the execution of a particular behaviour possible	
Physical opportunity	Opportunities provided by the environment, such as time, location and resource	Availability of soap
Social opportunity	Opportunities as a result of social factors, like cultural norms and social cues	Cultural norm to wash hands
Motivation	The internal processes which influence decision making and behaviours	
Automatic motivation	Automatic processes, such as our desires, impulses and inhibitions	Have habit of washing hands
Reflective motivation	Reflective processes, such as making plans and evaluating things that have already happened	Believe handwashing reduces illness risk

COM-B intervention function	Definition*	Examples
Education	Increasing knowledge or understanding	Curriculum on hand hygiene
Persuasion	Using communication to induce positive or negative feelings or stimulate action	Multimedia messaging (eg, flyers, radio messages) drawing on disgust or fear to promote handwashing
Incentivisation	Creating an expectation of reward	Providing prizes for people who wash hands
Coercion	Creating an expectation of punishment or cost	Shaming people who do not wash hands
Training	Imparting skills	Workshops demonstrating how to wash hands
Restriction	Using rules to reduce opportunity to engage in target behaviour/Increase target behaviour by reducing opportunity to engage in competing behaviours	Rules that children have to wash hands before eating lunch
Environmental restructuring	Changing physical or social context	Provision of soap Installation of handwashing station
Modelling	Providing example for people to aspire to or imitate	Videos showing proper handwashing technique Community leaders demonstrating proper handwashing technique
Enablement	Increasing means/reducing barriers to increase capability or opportunity (beyond education, training or environmental restructuring)	Community health workers conducting weekly visits to bring handwashing materials and provide additional engagement on hand hygiene

* Definitions from Michie *et al*.^34^

† If multimedia materials (such as art, flipcharts, etc) were incorporated into a training or education curriculum, they were coded as ‘training’ or ‘education’. However, if an intervention provided multimedia outside of an education or training program, then these were coded as ‘Multimedia messaging’.

‡ Definitions from Social Change UK.^69^

COM-B, Capability, Opportunity, Motivation and Behaviour.

To determine if an intervention was reported to be effective, we assessed whether authors of the included studies either reported their intervention to have a statistically significant effect in improving hand hygiene outcomes or if they reported an increase in hand hygiene outcomes comparing the treatment group to a control scenario. Studies that did not report an improvement in hand hygiene outcomes were classified as not effective.

To answer question (a), we extracted information on what theories were applied and report on the frequency of theory application and the proportion of the interventions that were reported effective at improving hand hygiene outcomes.

To answer question (b), we first extracted data on which barriers and enablers were *reported* by study authors to influence the focal hand hygiene behaviour. Beyond what study authors reported, we also determined what barriers and enablers the described interventions *addressed*; referred to in subsequent tables as ‘reported’ and ‘addressed’. The reported and addressed barriers and enablers were independently identified by two reviewers and then reconciled if there was disagreement. Because an intervention component could address the same factor even if it was reported to be a barrier or enabler, we present addressed barriers and enablers as one category. For example, soap provision addresses both lack of soap (barrier) and presence of soap (enabler). We categorised all identified reported barriers and enablers, and whether or not they were addressed according to constructs of the Capability, Opportunity, Motivation and Behaviour (COM-B) framework^30^ (defined in table 1) using a codebook that was pre-reviewed by one of the creators of the COM-B framework.^34^

To answer question (c), we used the Behavioural Change Techniques Taxonomy, version 1,^35^ to identify and categorise BCTs used in each study. A BCT is defined as ‘an observable and replicable component (of an intervention) designed to change behaviour. It is the smallest component compatible with retaining the postulated active ingredients and can be used alone or in combination with other BCTs’ (note that ‘active ingredients’ here refer to intervention components directly responsible for the intervention’s impact on behaviour).^36^ Categorising intervention components using the BCT taxonomy standardises the components using a common vocabulary; this standardisation enables assessment, understanding and comparison of intervention components to facilitate how those designing, adopting, reviewing and evaluating interventions communicate.^35^ Because interventions can leverage one or more BCTs, we identified the ‘BCT Package’, defined as the suite of BCTs used for the described interventions, for each intervention. We then determined the frequency of each BCT package and the proportion that were reported effective by community setting type. Finally, we identified the intervention function(s) (defined in table 1), the broad categories of BCTs, associated with each BCT package.^36^

To answer questions (d) and (e), we identified all interventions that included hand hygiene stations and extracted data on reported design features and their effectiveness at improving and sustaining hand hygiene practices in community settings. We identified all intervention studies that reported assessing hand hygiene station adaptations and extracted data and reported on which adaptations were effective.

To answer question (f), we first identified all interventions that indicated varying the frequency or intensity of their behaviour change intervention. Among those, we extracted data on frequency and intensity and reported which were effective at improving hand hygiene.

To answer question (g), we extracted data on focal population groups, risk scenarios and time to report on variability.

Due to the methodological differences across study designs, interventions, populations and outcome measures, we did not conduct a statistical meta-analysis. We instead used vote-counting to summarise reported intervention effectiveness across a diverse evidence base.^37 38^

### Ethics and patient involvement statements

As this is a review of published documents, no ethical approval was required. Patients or the public were not involved directly in the design, or conduct, or reporting, or dissemination plans of our research. This evidence synthesis supports the forthcoming WHO Guidelines for Hand Hygiene in Community Settings, which developed the study questions in broad consultation with key partners and networks of partners.

### Data availability

All data and extraction templates will be made publicly available on publication at Figshare (DOI: 10.6084/m9.figshare.28254041).^39^

## Results

### Characteristics of the studies included in this review

We identified 261 reports of eligible studies, derived from 223 studies that met our inclusion criteria, after grouping duplicate mentions of interventions across different studies. Of these, 93% (208) were quantitative studies and 7% (15) were mixed-methods studies (online supplemental file S3—PRISMA flow chart and online supplemental file S6—overview of included studies). We found studies in all WHO regions, with the highest representation from Africa (29%), South-East Asia (28%) and the Americas (22%) (table 2). Most studies documented interventions in domestic (45%) or institutional (54%) settings with very few documented in public settings (4%) (some documented in multiple settings). Schools (37%) and universities (10%) were the most common locations after households. More than half of the included studies (72%) identified hand hygiene as the primary outcome of interest and 52% of studies targeted other behaviours in addition to hand hygiene. The largest proportion of studies (33%) focused on both girls and boys, 25% did not clearly specify the focal population, 20% focused on women only and 20% focused on women and men.

**Table 2:** Characteristics of the included studies (n=223)

Descriptive characteristics of studies	Total, n (%)
Total number of studies	223 (100.0)
Region*	
African Region	64 (28.7)
South-East Asian Region	63 (28.3)
Region of the Americas	49 (21.9)
Western Pacific Region	27 (12.1)
European Region	22 (9.9)
Eastern Mediterranean Region	8 (3.6)
Unspecified	1 (0.5)
Location*	
Rural	84 (37.7)
Urban	62 (27.8)
Peri-urban	13 (5.8)
Unspecified	83 (37.2)
Setting*	
Domestic (households)	101 (45.3)
Institutional	120 (53.8)
Childcare centres	3 (1.3)
Schools	83 (37.2)
Universities	22 (9.9)
Workplaces	12 (5.4)
Public	8 (3.6)
Markets	4 (1.8)
Internally displaced persons camps	2 (0.9)
Community health clubs	1 (0.4)
Public transportation hubs	1 (0.4)
Unspecified	1 (0.4)
Gender of primary research population	
Adults—men	3 (1.3)
Adults—women	45 (20.2)
Adults—women and men	43 (19.2)
Children—boys	3 (1.3)
Children—girls	2 (0.9)
Children—girls and boys	72 (32.7)
Unspecified	55 (24.7)
Primary outcome studied*	
Hand hygiene	161 (72.2)
Diarrhoeal diseases	19 (8.5)
Food hygiene	21 (9.5)
Nutrition	17 (7.6)
Respiratory infections	14 (6.3)
Other infectious diseases	12 (5.4)
Other WASH-related behaviours	12 (5.4)
Other non-infectious health outcomes	11 (3.9)
School absenteeism	8 (3.6)
Mental/social well-being	3 (1.3)
Soil-transmitted helminths	1 (0.4)
Work absenteeism	1 (0.4)
Specific hand hygiene practice assessed†	
Handwashing with soap and water	143 (64.1)
Handrubbing with alcohol-based rub	19 (8.5)
Handwashing with water only	15 (6.7)
Handwashing with soap alternatives	5 (2.2)
Hand drying	3 (1.3)
Handwashing with non-alcoholic antiseptics	3 (1.3)
Handwashing—unspecified	63 (28.3)
Intervention also targeted other behaviours aside from hand hygiene	112 (50.2)
Hand hygiene intervention category‡	
Material provision	89 (39.9)
Provision of soap	53 (23.7)
Installation of handwashing stations	46 (20.6)
Provision of alcohol-based hand rub	16 (7.2)
Education programmes	99 (44.4)
School-based education	44 (19.7)
Food hygiene education	3 (1.3)
Education—adults	52 (23.3)
Training programmes	86 (38.6)
School-based training	35 (15.7)
Food hygiene training	8 (3.6)
Training—adults	43 (19.3)
Multimedia messaging	93 (41.7)
Posters	38 (17.0)
Videos	19 (8.5)
Art installations/flipcharts	8 (3.6)
Radio messages	7 (3.1)
Pamphlets	5 (2.2)
SMS	3 (1.3)
Multiple	10 (4.5)
Unspecified§	4 (1.8)
Hygiene promotion	61 (27.4)
Policy development or institutional strengthening	12 (5.4)
Household visits from community health workers	11 (4.9)
Group discussions	9 (4.0)
Plays, skits, songs or dramas	6 (2.7)
Games or competitions	4 (1.8)
Diaries and journals	4 (1.8)
Provision of other incentives (gift cards, creams, face masks, prizes, etc)	3 (1.3)
Public pledging ceremonies	2 (0.9)
Other	12 (5.4)
Study quality rating (mean)	3.3

* Descriptive statistics for region, location, setting, primary outcome studied and hand hygiene intervention represent variables where multiple options could be selected for each study.

† To be included, all studies had to measure hand hygiene practice; however, studies could have assessed multiple measures of hand hygiene practice.

‡ Interventions are not mutually exclusive, so studies could have multiple intervention categories as well as multiple subcategories included per study.

§ Multimedia-unspecified refers to interventions where studies reported that they used media messaging but did not specify the mode of delivery.

WASH, Water, Sanitation and Hygiene.

Among the included studies, handwashing with soap and water was the most common hand hygiene practice targeted by interventions (64%), followed by hand cleansing with alcohol-based rub (9%); 28% of studies did not provide detail about the specific hand hygiene practice of focus (n=63). Hand hygiene intervention categories varied by study; none of the category types were dominant. Specifically, 40% of interventions included material provision (eg, soap, hand hygiene stations), 44% included education (eg, school-based education), 39% included trainings (eg, food hygiene trainings), 42% included multimedia messaging (eg, posters, videos) and 27% included other hygiene promotion activities (eg, household visits from community health workers; group discussions, games). Soap was only provided in 24% of interventions. Overall, 82% of interventions were reported to be effective. Additional study characteristics and the studies leveraged for each research sub-question can be found in the supplement (online supplemental file S6—overview of studies; online supplemental file S7—studies leveraged for each research question).

### Quality of the studies included in this review

The mean study quality was 3.3 out of 5, overall indicating good quality (3.2 for non-randomised studies (n=121), 3.4 for randomised control trials (n=87) and 3.6 for mixed-methods studies (n=15)). Quality appraisal scores for each included study are included in the supplement (online supplemental file S8—MMAT Assessment).

### Evidence to answer sub-questions

#### Among interventions to improve hand hygiene in community settings, which have been designed using behaviour change theories?

Of the 223 studies identified in our review, 28% (n=63) reported using a theory or model to inform the design of the hand hygiene intervention (online supplemental file S9—theory table). The earliest study included in our review that reported using theory was published in 2009, but there is no noticeable change in the proportion of studies that have used theory over time (online supplemental file S10—theory bar chart). There was no difference in reported effectiveness among interventions using theory (52/63; 83%) and those not (131/160; 82%). The theories, models and frameworks most commonly referenced include the *Theory of Planned Behaviour* (n=14; 22%; 71% reported effective),^40^ *Health Belief Model* (n=11; 18%; 91% reported effective),^41^ *Behaviour Centred Design/Evo-Eco Model* (n=7; 11%; 71% reported effective),^42^ *RANAS (Risks, Attitudes, Norms, Abilities and Self-regulation*) (n=6; 10%; 83% reported effective),^43^ *COM-B (Capability, Opportunity, Motivation and Behaviour*) (n=6; 10%; 83% reported effective),^34^ and *IBM-WASH (Integrated Behavioural Model for Water, Sanitation and Hygiene*) (n=5; 8%; 80% reported effective).^44^ Other behaviour change theories, models and frameworks were noted (n=19); however, each one was only referenced across one or two studies. Online supplemental file S5 indicates which theory was used for each study, if any.

#### Among interventions to improve hand hygiene in community settings, which have effectively leveraged identified barriers and enablers of hand hygiene in community settings?

**Figure 1:**
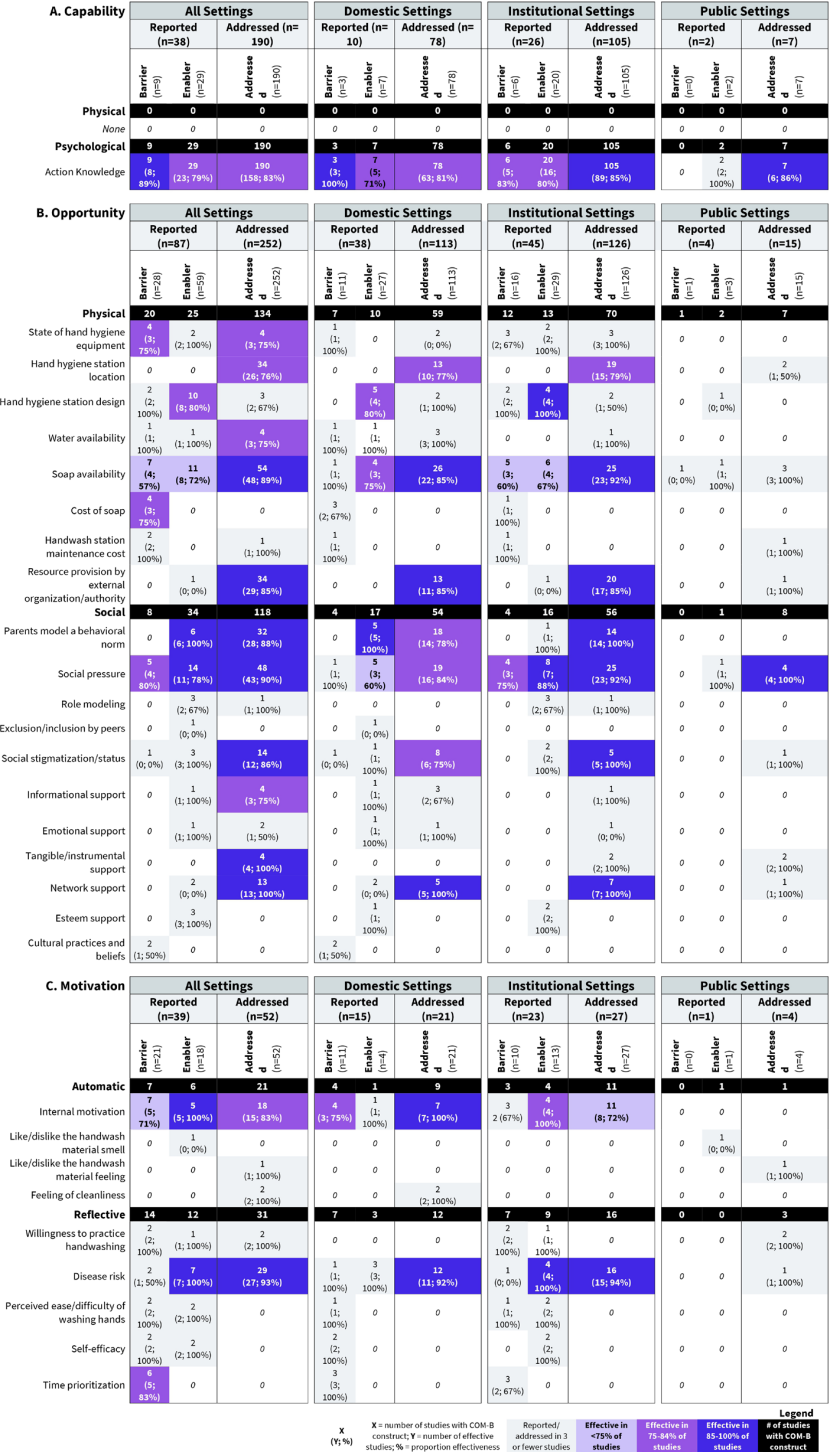
Summary of effective barriers and enablers targeted in hand hygiene interventions and categorised by (**A**) capability, (**B**) opportunity and (**C**) motivation components of the COM-B framework (n=223). If study authors explicitly mentioned barriers or enablers that influenced their intervention design, these were captured as ‘Reported barrier/enabler’. Otherwise, review authors assumed what barriers or enablers were addressed by the intervention design which were captured as ‘Addressed’. There could have been multiple barriers/enablers reported per study as studies could report multiple intervention categories. Proportion effectiveness refers to the number of studies that reported the intervention was effective at improving hand hygiene outcomes related to that barrier/enabler. Of those that were reported effective, we also provide proportions by setting type (domestic, institution or public). Effectiveness proportions were only provided for barriers/enablers that had at least three or more studies reported/addressed.COM-B, capability, opportunity, motivation and behaviour.

In all settings, barriers and/or enablers were identified across all broad COM-B constructs and most subconstructs (figure 1). Overall, studies largely reported a given barrier or enabler less often than it was addressed. In other words, interventions acted on a given barrier or enabler more often than the barrier or enabler was explicitly acknowledged to be an issue. Studies did not always report the same barrier and enabling factor for a given COM-B construct. Representative examples of misalignment between reported and addressed barriers/enablers by COM-B component can be found in online supplemental file S11.

Under Capability, no studies reported nor addressed any *Physical capability* barriers or enablers. ‘Action Knowledge’ was identified as the only reported or addressed *Psychological capability* barrier or enabler. ‘Action knowledge’ was reported to be a barrier (9) or enabler (29) far less frequently than it was addressed (190), indicating that interventions acted on ‘Action knowledge’ far more often than they reported it as a precondition. Across all studies and settings in which ‘Action Knowledge’ was either reported or addressed to be a barrier or an enabler, 70% or more interventions were reported to be effective.

Under Opportunity, several *Physical* and *Social opportunity* barriers and enablers were reported and addressed across settings. The *Physical opportunities* barriers/enablers most frequently reported or addressed include: ‘State of hygiene equipment’, ‘Hand hygiene station location’, ‘Soap availability’ and ‘Resource provision’. As with ‘Action Knowledge’, there was notable discordance in the number reported and addressed for several barriers/enablers. ‘Soap availability’ was reported as a barrier (7) or enabler (11) less often than it was addressed (54). ‘Hand hygiene station location’ was addressed in 34 interventions but was never explicitly reported to be a barrier or enabler. Among barriers and enablers classified as *Social opportunities*, ‘Social pressure’ and ‘Parent model a behavioural norm’ were the most reported and addressed, and across settings in which these were either reported or addressed to be a barrier or an enabler, 60% or more interventions were reported to be effective.

Under Motivation, several *Automatic* and *Reflective motivation* barriers and enablers were reported and addressed across settings. ‘Internal motivation’ was the most reported and addressed *Automatic motivation* factor (reported barrier: 7; reported enabler: 5; addressed: 18). Across all settings in which ‘Internal motivation’ barriers and enablers were either reported or addressed to be a barrier or an enabler, 60% or more interventions were reported to be effective. ‘Disease Risk’ was the most identified *Reflective motivation* factor (reported barrier: 2; reported enabler: 7; addressed: 29), though reported effectiveness was variable.

#### Among interventions to improve hand hygiene in community settings, what BCTs have been implemented to effectively improve and sustain handwashing practices?

From the 223 studies, 438 BCT packages were identified, representing 18 different BCT package ‘types’ (online supplemental file S12—frequency of BCT packages). Nearly half (45%) of studies evaluated just one package, 28% evaluated two; 17% evaluated three, the remaining 10% evaluated between 4 and 6 packages. Roughly a third (32%) of studies evaluated a package with just one BCT, 75% evaluated packages that had two BCTs and 49% evaluated a package that had three BCTs.

Among the 18 different package types identified, 17 different BCTs were used (a–q in table 3) and 4 BCTs were dominant. The most common BCT is ‘a. Instruction on how to perform the behaviour’, included in 55% of packages (n=240), followed by ‘b. Information about health consequences’ which was included in 183 packages (42%). The third most common BCT is ‘c. Adding objects to the environment’, which was included in 38% (n=166) of packages with the fourth most common BCT, ‘f. Demonstration of behaviour’, included in 99 packages (23%). The remaining BCTs featured in 13% or fewer packages (range: 2 (0.5%)–55 (12.6%)).

**Table 3:** Frequency of behaviour change techniques (BCTs) used across the package types (n=438)

BCT	Package type ID	Number of packages per type evaluated	Inclusion across packages
a. Instruction on how to perform the behaviour	1	105	240 (54.8%)
	2	61	
	8	43	
	9	19	
	16	12	
b. Information about health consequences	1	105	183 (41.8%)
	2	61	
	12	11	
	14	3	
	17	4	
c. Adding objects to the environment	2	61	166 (37.9%)
	3	53	
	4	35	
	5	9	
	6	8	
d. Restructuring the physical environment	4	35	44 (10.0%)
	5	9	
e. Prompts/cues	5	9	29 (6.6%)
	6	8	
	16	12	
f. Demonstration of behaviour	7	42	99 (22.6%)
	8	43	
	12	11	
	17	4	
g. Social support (practical)	7	42	55 (12.6%)
	12	11	
	14	3	
h. Feedback on behaviour	8	43	43 (9.8%)
i. Social support (unspecified)	10	12	12 (2.7%)
j. Restructuring the social environment	10	12	12 (2.7%)
k. Self-monitoring of behaviour	11	7	7 (2.9%)
l. Goal setting (behaviour)	11	7	7 (2.9%)
m. Action planning	13	9	11 (2.5%)
	15	2	
n. Information on others’ approval	13	9	11 (2.5%)
	15	2	
o. Commitment	15	2	2 (0.5%)
p. Material incentive	17	4	4 (0.9%)
q. Incentive (outcome)	18	3	3 (0.7%)

We do not report on effectiveness of every individual BCT, as most (14/17; 82%) were only used in combination with other BCTs; thus, isolating the individual contribution of each BCT in a package is not possible. Only three BCTs were evaluated independently, without other BCTs: ‘a. instructions on how to perform the behaviour’ (package 9: 84.2% reported effective); ‘c. Adding objects to the environment’ (package 3: 81.1% reported effective) and ‘q. Incentive’ (package 18: 67.7% reported effective) (table 4).

**Table 4:** Frequency of identified behaviour change technique (BCT) packages and represented activities by associated intervention functions, with their effectiveness and study quality rating across included studies (n=223)

Combinations (A–K) of the intervention functions (i–v) associated with BCT packages	Packages (1–18) with identified BCTs (a–q) (n=437)	Package, n (%)	Activities represented	Reported effectiveness* (by community setting type)	Mean MMAT quality rating† for studies with package
A. Education Training	1. a. Instruction on how to perform the behaviour b. Information about health consequences‡	105 (24.0)	School-based education	Overall: 91/105 (86.7)	3.2
			Edu.—adults	Domestic: 40/48 (83.3)	
			Plays, skits or songs	Institution: 49/55 (89.1)	
			Multimedia—unspecified§	Public: 2/2 (100.0)	
B. Education Training Environmental restructuring	2. a. Instruction on how to perform the behaviour b. Information about health consequences‡ c. Adding objects to the environment¶	61 (14.0)	Multimedia—posters	Overall: 50/61 (82.0)	3.4
			Multimedia—art/flipcharts	Domestic: 17/19 (89.5)	
			Multimedia—pamphlets	Institution: 30/38 (79.0)	
			Multimedia—multiple	Public: 3/4 (75.0)	
C. iii. Environmental restructuring	3. c. Adding objects to the environment	53 (12.1)	Soap provision	Overall: 43/53 (81.1)	3.3
			Alcohol rub provision	Domestic: 18/23 (78.3)	
				Institution: 22/26 (84.6)	
				Public: 3/4 (75.0)	
	4. c. Adding objects to the environment d. Restructuring the physical environment	35 (8.0)	Handwashing station provision**	Overall: 29/35 (82.9)	3.5
				Domestic: 12/15 (80.0)	
				Institution: 16/18 (88.9)	
				Public: 1/2 (50.0)	
	5. c. Adding objects to the environment d. Restructuring the physical environment e. Prompts/cues	9 (2.1)	Handwashing station provision** with design adaptations that includes cues to action	Overall: 7/9 (77.8)	3.4
				Domestic: 4/4 (100.0)	
				Institution: 3/5 (60.0) Public: 0/0 (0.0)	
	6. c. Adding objects to the environment e. Prompts/cues	8 (1.8)	Soap provision with adaptations that include cues to action	Overall: 8/8 (100.0)	3.9
				Domestic: 5/5 (100.0)	
				Institution: 2/2 (100.0)	
				Public: 1/1 (100.0)	
D. iv. Enablement ii. Training	7. f. Demonstration of behaviour g. Social support (practical)	42 (9.6)	Training—adults	Overall: 35/42 (83.3)	3.5
				Domestic: 23/29 (79.3)	
				Institution: 7/8 (87.5)	
				Public: 5/5 (100.0)	
E. ii. Training	8. a. Instruction on how to perform the behaviour f. Demonstration of behaviour h. Feedback on behaviour	43 (9.8)	School-based training	Overall: 36/43 (83.7)	3.2
			Food hygiene training	Domestic: 1/1 (100.0)	
				Institution: 35/42 (83.3) Public: 0/0 (0.0)	
	9. a. Instruction on how to perform the behaviour	19 (4.3)	Multimedia—videos	Overall: 16/19 (84.2)	2.8
				Domestic: 8/10 (80.0)	
				Institution: 7/8 (87.5)	
				Public: 1/1 (100.0)	
F. iv. Enablement	10. i. Social support (unspecified) j. Restructuring the social environment	12 (2.7)	Policy or institutional strengthening	Overall: 11/12 (91.7)	3.9
				Domestic: 2/3 (66.7)	
				Institution: 8/8 (100.0)	
				Public: 1/1 (100.0)	
	11. k. Self-monitoring of behaviour l. Goal setting (behaviour)	7 (1.6)	Multimedia—SMS	Overall: 7/7 (100.0)	3.7
			Diaries and journals	Domestic: 4/4 (100.0)	
				Institution: 3/3 (100.0) Public: 0/0 (0.0)	
G. i. Education ii. Training iv. Enablement	12. b. Information on health consequences‡ f. Demonstration of behaviour g. Social support (practical)	11 (2.5)	Community health worker visits	Overall: 6/11 (54.5)	3.5
				Domestic: 4/8 (50.0)	
				Institution: 1/2 (50.0)	
				Public: 1/1 (100.0)	
H. i. Education iv. Enablement	13. l. Goal setting (behaviour) m. Action planning n. Information on others’ approval	9 (2.1)	Group discussions	Overall: 7/9 (77.8)	2.8
				Domestic: 3/4 (75.0)	
				Institution: 3/4 (75.0)	
				Public: 1/1 (100.0)	
	14. b. Information about health consequences‡ g. Social support (practical)	3 (0.7)	School-based education	Overall: 1/3 (33.3) Domestic: 0/0 (0.0)	3.3
			Food hygiene education	Institution: 1/3 (33.3) Public: 0/0 (0.0)	
	15. m. Action planning n. Information on others’ approval o. Commitment	2 (0.5)	Public pledging ceremonies	Overall: 2/2 (100.0)	3.5
				Domestic: 2/2 (100.0) Institution: 0/0 (0.0) Public: 0/0 (0.0)	
I. ii. Training iii. Environmental restructuring	16. a. Instruction on how to perform the behaviour e. Prompts/cues	12 (2.7)	Other interventions	Overall: 12/12 (100.0)	3.0
				Domestic: 2/2 (100.0)	
				Institution: 9/9 (100.0)	
				Public: 1/1 (100.0)	
J. i. Education ii. Training v. Incentivisation	17. b. Information about health consequences‡ f. Demonstration of behaviour p. Material incentive	4 (0.9)	Games or competitions	Overall: 3/4 (75.0) Domestic: 0/0 (0.0)	3.3
				Institution: 3/4 (75.0) Public: 0/0 (0.0)	
K. v. Incentivisation	18. q. Incentive (outcome)	3 (0.7)	Other incentives provided	Overall: 2/3 (66.7)	3.0
				Domestic: 0/1 (0.0)	
				Institution: 2/2 (100.0) Public: 0/0 (0.0)	

* Reported effectiveness is determined if authors reported that the intervention was effective at improving hand hygiene outcomes.

† For individual ratings by study design, please see online supplemental file S8—MMAT Assessment.

‡ This BCT does not necessarily require that information is provided through a physical object (such as pamphlets, posters, etc) but also includes verbal communication of health consequences (such as through plays, skits or songs, or through multimedia communication like radio ads).

§ Multimedia-unspecified refers to interventions where studies reported that they used media messaging but did not specify the mode of delivery.

¶ In BCT package 2 specifically, none of the activities associated with BCT ‘c’ (adding objects to the environment) included adding water, soap or other hand hygiene related items. This package instead provided multimedia objects such as those described in the ‘Activities Represented’ column.

** In line with the Behaviour Change Wheel, one intervention activity can have multiple associated BCTs. Handwashing stations have been coded with these two BCTs as they are added objects to the environment and also handwashing stations restructure the physical environment.

MMAT, Mixed Method Appraisal Tool.

Four BCT intervention package types (packages 1–4, table 4) represent 58% (254/437) of the intervention packages assessed and were reported to be generally effective. Specifically, Package 1, which has two BCTs, represents 24% (105) of the packages evaluated. Package 2 includes three BCTs and represents 14% (61) of evaluated packages. Package 3 includes one BCT and represents 12% (53) of evaluated packages. Finally, Package 4 includes two BCTs and represents 8% (35) of evaluated packages. The intervention functions, BCTs and intervention activities leveraged in each study can be found in the supplement (online supplemental file S13—intervention functions, BCTs and activities).

#### Among interventions to improve hand hygiene in community settings, what hand hygiene station designs have been effective at improving and sustaining hand hygiene?

Hand hygiene station types and design features were reported in 46 (21%) of the included studies, the majority of which were for users in school (52%) and household (33%) settings. A summary of these designs and their reported effectiveness is provided in the supplement (online supplemental file S14—HH station designs). Of the 46 studies that reported providing a handwashing station, 37 (80%) were reported to be effective. The most common station was a raised bucket with tap/outlet (n=20; 75% reported effective). 10 (22%) studies did not report the type of station design; among those, 70% were reported to be effective.

Hand hygiene station designs varied by several features, including their mobility (fixed vs mobile), permanency (temporary vs permanent) and water supply (individual storage tank vs piped water), among others. However, sample sizes across these strata were small as a substantial proportion of studies did not report on station mobility (46%), permanency (44%), water supply (61%) or material (85%), nor did they indicate the specific location of the station in its setting (86%).

#### Among interventions to improve hand hygiene in community settings, what hand hygiene station design adaptations (eg, placement, nudges and cues) have been effective at improving and sustaining hand hygiene?

10 studies evaluated hand hygiene station design adaptations, 6 (40%) of which performed better than the standard design (online supplemental file S15—HH station design adaptations). Five studies evaluated the use of ‘nudges’ (eg, painted or paved footpaths), three evaluated cues to action and two studies evaluated changes to placement. Further description of these studies can be found in online supplemental file S15.

#### Among interventions to improve hand hygiene in community settings, what level of frequency and intensity of behaviour change interventions is necessary to effectively improve hand hygiene?

Six studies evaluated adaptations in the frequency (n=1) or intensity (n=5) of behaviour change interventions, three of which—all assessing higher intensity—performed better than the standard design. Further description of these six studies and the adaptations can be found in online supplemental file S16.

#### Among interventions to improve hand hygiene in community settings, how do hand hygiene practices vary by population groups, risk scenarios or over time?

Among all included studies, we found little variability in hand hygiene practices by population groups, risk scenarios and over time. Descriptions of the population groups, risk scenarios and time points included in this review are presented in online supplemental file S17. Full citations of all included studies are in online supplemental file S18—full ref list.

## Discussion

Our review of hand hygiene interventions in community settings highlights the wide geographic and contextual diversity of interventions that have been implemented, reflecting that there are myriad ways to improve hand hygiene. Most interventions took place in households or schools; there is limited research set in workplaces and public settings in general. Below, we discuss considerations for each of the focal research questions in turn. Notably: (a) theory was not extensively reported to be used in intervention design; the proportion of interventions that reported to use theory and were reported to be effective was no greater than those that did not report using theory. (b) A large proportion of interventions addressed ‘Action Knowledge’, or how to wash hands, despite this factor not being identified within our sample or in other studies as a notable barrier or enabler of hand hygiene. Further, interventions did not always address identified barriers or enablers, potentially missing critical opportunities to drive behaviour. (c) There was substantial heterogeneity in the BCTs used across the interventions; further research would benefit from intentional design and selection of specific BCTs to evaluate. (d/e) The handwashing station designs used in this study reported that they were mostly effective at improving hand hygiene; only 10 studies assessed design adaptations, of which six outperformed the standard design. (f) Too few studies (6) assessed changes in frequency or intensity of behaviour change interventions to draw firm conclusions. (g) Interventions that included or focused on people with disabilities were scarce. We identified inconsistencies in intervention reporting and thus conclude by presenting recommendations for intervention reporting to facilitate future learning and synthesis. Though we are not able to identify clear recommendations for each of our research questions, most of the interventions (82%) within this review were reported to be effective, demonstrating that there are multiple ways to improve hand hygiene. For policymakers seeking to understand how to improve hand hygiene within their context, the library of studies included in this systematic review can be used to inform local efforts.

### Use of theories or models to inform intervention design

Consistent with other research, theory was not explicitly reported to have been used extensively to inform intervention design.^45^ As such, our analysis suggests that reported theory use did not result in a difference in intervention effectiveness. 28% (n=63) of included studies in this review reported using a theory or model for designing a hand hygiene intervention with little difference in reported effectiveness. In a 2014 meta-analysis, Prestwich and colleagues assessed physical activity and healthy eating interventions to discern the extent to which theory influenced the effectiveness of health behaviour interventions. They found a marginally greater proportion of studies leveraged theory (56%) and only a weak relationship between the theory used and the extent of theory use, with intervention effectiveness. They also note that theory was not used extensively in the intervention design process.^45^ However, we suggest that further research is needed before ruling out theory use in the design of hand hygiene interventions. Within this review, there may be studies that used theory but did not explicitly report if and how theory was used, limiting our ability to understand the application of theory in hand hygiene research. Incorporating a theory of change into intervention design could ensure that the intervention is effective. Using a systematic approach to document and report on the use of theory of change would ensure that future research is able to assess the relationship between theory application and intervention effectiveness.^46^ Assessing this relationship was beyond the scope of this review, but future research understanding these assessments would provide useful information for intervention design.

### Effective leveraging of identified barriers and enablers

Within the review, we identified a lack of alignment between what studies reported to be barriers and/or enablers and what interventions actually addressed, suggesting that studies need to improve reporting of known barriers and enablers or better leverage evidence in intervention design. It is widely acknowledged that understanding the factors that influence health behaviours is essential for designing impactful interventions.^4243 47^,^49^ But it is not clear that studies optimally leveraged factors that influenced behaviour. ‘Hand hygiene station design’ was reported to be an enabler in ten studies and a barrier in two but was addressed in only three. Many factors addressed by interventions in our review were not reported as barriers or enablers within the studies. ‘Action Knowledge’ was reported to be a barrier or enabler in 38 (17%) studies yet was addressed in 190 (85%). Additionally, no studies reported hand hygiene station locations to be a barrier or an enabler, yet 34 (15%) interventions addressed hand hygiene station location. Taken together, our findings suggest the need for more extensive justification of why specific barriers or enablers are or are not addressed, especially to ensure that intervention design decisions are deliberate and aligned with local context.

We also found there to be a mismatch between the barriers and enablers addressed among the interventions reviewed in our study, and the barriers and enablers identified by other studies to be the greatest determinants of hand hygiene. Two recent systematic reviews that explored barriers and enablers to hand hygiene in community settings both found resources in the environment to be the most reported barriers or enablers.^30 50^ The findings from these systematic reviews raise a critical point—people cannot clean their hands if they do not have the resources to do so, regardless of their motivation or level of knowledge. Because access to these resources is fundamental for sustaining hand hygiene practice, interventions and programmes need to either provide access to adequate resources or ensure they are already in place. Otherwise, efforts to motivate hand hygiene or instil knowledge will not be effective, could waste valuable resources and may even be unethical if people are subject to messaging yet lack the ability to act. The resources provided should suit the needs of a given population, enabling hand washing to be easy, convenient and quick, particularly given time and responsibility constraints that have also been reported.^3050^,^56^ Across the public health sector, interventions that change the environmental context are the most recommended as they demonstrate large impact with minimal individual effort.^57^ Hand hygiene efforts should take heed to ensure the environmental context is sufficient and optimised to enable hand hygiene.

Within this review, we are not able to identify specific barriers or enablers that hand hygiene interventions must address to be effective. Though this review cannot provide specific recommendations, a recent review synthesises qualitative studies to understand the most common barriers and enablers that impact hand hygiene practice.^30^

### Implementation of BCTs

The interventions in this review used a range of BCTs across multiple package types and settings, limiting identification of which specific BCTs are most effective in improving hand hygiene and what was the added value of each additional BCT. Four BCT intervention package types addressing various combinations of BCTs represent the majority of the intervention packages identified in our review. The substantial variation in the types and number of BCTs used varied between packages. Another systematic review from the physical activity literature found substantial heterogeneity in the number of BCTs used per intervention, but they did not find that any specific combinations of BCTs were effective.^58^ Other systematic reviews have highlighted the difficulty in evaluating the effectiveness of BCTs and have recommended more intentional design of BCT evaluations to assess which ones are effective,^59^ going as far as proposing an ontology of behaviour change interventions to develop a standard terminology and classification system for behaviour change interventions.^60^ Those working in hand hygiene could consider leveraging the findings herein as a sector-specific taxonomy of hand hygiene intervention types to inform and identify future interventions. To better understand which BCTs are effective at improving hand hygiene, interventions need to be deliberately and thoughtfully designed, with consideration to which BCTs are most needed to address relevant barriers and enablers in a specific context.

### Lack of disability representation

Within our review, only four studies examined hand hygiene practice among a key vulnerable group, people with disabilities. Two studies evaluated intervention adaptations for intellectually disabled children and adults,^61 62^ one adapted an education programme for hearing-impaired children,^63^ and one did not specify the types of disability included in their focal population, nor did they specify how their intervention was adapted.^64^ People with disabilities face additional barriers in being able to access hand hygiene which varies by disability type. As found in Tanzania, people with physical disabilities may not be able to access handwashing stations, whereas those with visual disabilities may not be able to access educational leaflets or brochures.^65^ For hand hygiene interventions to be truly accessible for everyone, additional research is needed to understand how these interventions should be adapted for all types and levels of disability.

### Need for improved, systematic reporting of hand hygiene interventions

A common limitation across the included literature was inconsistent reporting of hand hygiene interventions in the included studies. A recent scoping review of the 40 most cited evaluations of WASH interventions published in the last 10 years (2012–2022) found that inconsistent reporting of WASH implementation illustrates a major challenge in the sector.^66^ Although our review revealed a wealth of knowledge on effective theories, barriers and enablers, and BCTs used in hand hygiene interventions, our findings were constrained by the quality of intervention reporting in the included studies. A quarter of the studies in our review failed to clearly specify the hand hygiene practices targeted for improvement or defined the focal population. Further, we found only six studies that changed the frequency or intensity of hand hygiene interventions, constraining our ability to understand what level of frequency and intensity of behaviour change interventions are needed to effectively enhance hand hygiene in community settings (sub-question f). Inconsistent reporting also affected our ability to learn about effective hand hygiene station design features and adaptations (sub-questions d and e). Though most studies reported that providing a hand hygiene station was effective in improving hand hygiene practice, our analysis was limited by incomplete reporting on station characteristics and design features. Study reporting should include detailed description of the intervention and reporting guidelines specific to hand hygiene are available.^67^

### Strengths and limitations

This review was part of an integrated protocol for multiple related reviews which included an exhaustive search strategy encompassing multiple databases and grey literature sources and a two-phased approach to identify relevant literature of hand hygiene in community settings. This is the first review to comprehensively examine interventions designed to improve hand hygiene in community settings. A key limitation of this effort, however, was the need to impose a structure (COM-B model and Behaviour Change Wheel)^34^ for categorising and presenting interventions that only 6 (3%) of included studies used. To use COM-B and the associated resources, we needed to classify reported barriers and aspects of the interventions ourselves. Our ability to make these classifications was limited by the information available in the studies, potentially limiting our ability to make classification decisions that would align with what the original study authors might have done. In some cases, several studies may have resulted from a single intervention. For the purpose of this review, we expected that papers leveraging the same intervention would report on that intervention consistently each time. Therefore, we only reported on an intervention once and identified a single paper from which to do so. If authors did not report on an intervention consistently across different papers, it is possible that elements of an intervention were missed and the COM-B classifications presented in this review may not align with what the original study authors would have intended. Despite the potential challenges with imposing an external framework, the use of COM-B is also a strength of this review. As the hand hygiene interventions included in the study are quite varied, using COM-B provided a means to identify and group like components to determine what basic elements were comparable and most leveraged.

In this review, we only included studies published in English. Though our review identified studies representing all WHO regions, we may have missed critical insights from studies published in other languages. Another key limitation is how we reported intervention effectiveness using vote-counting. Though vote-counting provided us with broad trends of reported intervention effectiveness, it does not account for effect sizes, study design, sample size or how outcomes were measured (eg, self-reported vs direct observation) which may oversimplify the complex findings in our included studies. We recommend interpreting the vote-counting results with caution and warn that they should not be used to draw definitive conclusions about effectiveness. Finally, it was beyond the scope of this review to analyse the various measures used for assessing hand washing behaviours. To inform and improve measurement in hand hygiene intervention trials as well as in other studies, we recommend an audit of measures, as has been done elsewhere.^68^ Such an audit could outline the strengths and weaknesses of various measurement approaches (eg, validity, reliability, cost) and give researchers a means to justify the approach they use and transparently note limitations.

## Conclusion

We identified a wide range of hand hygiene interventions that have been implemented across geographically diverse community settings. However, our ability to compare interventions was limited due to variability in research outcomes, settings, intervention components, functions and BCTs leveraged, preventing us from making specific recommendations to use or not use specific intervention types in community settings. That said, findings suggest critical ways that policymakers and practitioners can strengthen future interventions. Specifically, interventions can better leverage barriers and enablers of hand hygiene practices to ensure alignment with intervention components. Failing to address known barriers and enablers can limit or prevent impact, and adding additional components that are not needed may not be resource (eg, cost, time) effective. Finally, policymakers and practitioners should consider if resources in the physical environment are available for those engaged to wash hands, like water, soap and hygiene stations. As learnt from intervention research in the broader public health field, there will be greater likelihood of impact if these aspects of the environment do exist. If settings do not already have these critical hand hygiene components in the environment, interventions that seek to improve hand hygiene only through motivation, social pressure or by increasing knowledge should be reconsidered.

## Supplementary material

10.1136/bmjgh-2025-018928 online supplemental file 1

10.1136/bmjgh-2025-018928 online supplemental file 2

10.1136/bmjgh-2025-018928 online supplemental file 3

10.1136/bmjgh-2025-018928 online supplemental file 4

10.1136/bmjgh-2025-018928 online supplemental file 5

10.1136/bmjgh-2025-018928 online supplemental file 6

10.1136/bmjgh-2025-018928 online supplemental file 7

10.1136/bmjgh-2025-018928 online supplemental file 8

10.1136/bmjgh-2025-018928 online supplemental file 9

10.1136/bmjgh-2025-018928 online supplemental file 10

10.1136/bmjgh-2025-018928 online supplemental file 11

10.1136/bmjgh-2025-018928 online supplemental file 12

10.1136/bmjgh-2025-018928 online supplemental file 13

10.1136/bmjgh-2025-018928 online supplemental file 14

10.1136/bmjgh-2025-018928 online supplemental file 15

10.1136/bmjgh-2025-018928 online supplemental file 16

10.1136/bmjgh-2025-018928 online supplemental file 17

10.1136/bmjgh-2025-018928 online supplemental file 18

## Data Availability

Data are available in a public, open access repository. All data relevant to the study are included in the article, uploaded as supplementary information, or can be found in the linked public, open access repository.
