# Supplementary material for: Interventions to improve hand hygiene in community settings: a systematic review of theories, barriers and enablers, behaviour change techniques and hand hygiene station design features
Source: BMJ Glob Health. 2025 Sep 16;10(Suppl 7):e018928. doi: 10.1136/bmjgh-2025-018928 (PMC12443188; doi:10.1136/bmjgh-2025-018928)
Supplement: online supplemental file 1 [file bmjgh-10-Suppl_7-s001.docx]

**Interventions to improve hand hygiene in community settings: A systematic review of theories, barriers and enablers, behavior change techniques, and hand hygiene station design features**

*Authors*

Sridevi K. Prasad^1^ 0000-0003-0457-9534

Jedidiah S. Snyder^2^ 0000-0002-7688-4450

Erin LaFon^2^

Lilly A. O’Brien^2^ 0009-0004-1987-3706

Hannah Rogers^3^ 0000-0002-9515-1439

Oliver Cumming^4,5^ 0000-0002-5074-8709

Joanna Esteves Mills^5^

Bruce Gordon ^5^

Marlene Wolfe^2^ 0000-0002-6476-0450

Matthew C. Freeman^2^ 0000-0002-1517-2572

Bethany A. Caruso^1*^ 0000-0001-9738-9857

1 Hubert Department of Global Health, Rollins School of Public Health, Emory University, Atlanta, GA, USA; [bcaruso@emory.edu](mailto:bcaruso@emory.edu) (BAC); [sridevi.prasad@emory.edu](mailto:sridevi.prasad@emory.edu) (SKP)

2 Gangarosa Department of Environmental Health, Rollins School of Public Health, Emory University, Atlanta, GA, USA; [matthew.freeman@emory.edu](mailto:matthew.freeman@emory.edu) (MCF); [marlene.wolfe@emory.edu](mailto:marlene.wolfe@emory.edu) (MW) [jedidiah.snyder@emory.edu](mailto:jedidiah.snyder@emory.edu) (JSS); [lilly.obrien@emory.edu](mailto:lilly.obrien@emory.edu) (LAO); [erin.lafon@emory.edu](mailto:erin.lafon@emory.edu) (EL)

3 Woodruff Health Sciences Center Library, Emory University, Atlanta, GA, USA; [hannah.rogers@emory.edu](mailto:hannah.rogers@emory.edu) (HR)

4 Department of Disease Control, London School of Hygiene and Tropical Medicine, London, UK; [oliver.cumming@lshtm.ac.uk](mailto:oliver.cumming@lshtm.ac.uk) (OC)

5 Water, Sanitation, Hygiene and Health Unit, World Health Organization, Geneva, Switzerland; [estevesj@who.int](mailto:estevesj@who.int) (JEM); [gordonb@who.int](mailto:gordonb@who.int) (BG)

*Corresponding author: Bethany A. Caruso [bcaruso@emory.edu](mailto:bcaruso@emory.edu)

Emory University, Rollins School of Public Health, 1518 Clifton Rd, Atlanta, GA 30322

**Table S1.** Research question and eligibility criteria in SPIDER/PICOS format

| **Research question** | | **Sample** | **Phenomenon of interest** | **Design** | **Evaluation** | **Research type** |
| --- | --- | --- | --- | --- | --- | --- |
| RQ3.2. Among interventions to improve hand hygiene in community settings, what theories, barriers and enablers, intervention functions and behavior change techniques, and design features have been leveraged effectively to improve and sustain hand hygiene in community settings? | (3.2a) Among interventions to improve hand hygiene in community settings, which have been designed using behavior change theories? | General population in community settings | Behavior change theories among interventions to improve hand hygiene in community settings | Experimental or quasi-experimental designs, randomized and non-randomized controlled trials, before-after studies | Effective hand hygiene (i.e., any practice which removes or deactivates pathogens from hands and thereby limits diseases transmission) | Mixed methods (focus on quantitative evaluation and will consult any papers linked to the evaluation that may be relevant, e.g., protocols, follow-up studies (qual or quant) to assess sustainability) |
|  | (3.2b) Among interventions to improve hand hygiene in community settings*,* which have effectively leveraged identified barriers and enablers of hand hygiene in community settings? | General population in community settings | Effective leveraging of identified barriers and enablers of hand hygiene among interventions to improve hand hygiene in community settings | Experimental or quasi-experimental designs, randomized and non-randomized controlled trials, before-after studies | Effective hand hygiene (i.e., any practice which removes or deactivates pathogens from hands and thereby limits diseases transmission) | Mixed methods (focus on quantitative evaluation and will consult any papers linked to the evaluation that may be relevant, e.g., protocols, follow-up studies (qual or quant) to assess sustainability) |
|  | (3.2c) Among interventions to improve hand hygiene in community settings, what behaviour change techniques have been implemented to effectively improve and sustain handwashing practices? | General population in community settings | Behavior change techniques to promote handwashing among interventions to improve hand hygiene in community settings | Experimental or quasi-experimental designs, randomized and non-randomized controlled trials, before-after studies | Effective and sustained hand hygiene (i.e., consistent hand hygiene practices) | Mixed methods (focus on quantitative evaluation and will consult any papers linked to the evaluation that may be relevant, e.g., protocols, follow-up studies (qual or quant) to assess sustainability) |
|  | (3.2d) Among interventions to improve hand hygiene in community settings, what hand hygiene station designs have been effective at improving and sustaining hand hygiene? | General population in community settings | Hand hygiene station design among interventions to improve hand hygiene in community settings | Experimental or quasi-experimental designs, randomized and non-randomized controlled trials, before-after studies | Effective and sustained hand hygiene | Mixed methods (focus on quantitative evaluation and will consult any papers linked to the evaluation that may be relevant, e.g., protocols, follow-up studies (qual or quant) to assess sustainability) |
|  |  | **Participants** | **Interventions** | **Comparison** | **Outcome** | **Study design** |
|  | (3.2e) Among interventions to improve hand hygiene in community settings, what hand hygiene station design adaptations (e.g., placement, nudges, and cues) have been effective at improving and sustaining hand hygiene? | General population in community settings | Design adaptations (e.g., placement, nudges, and cues) of hand hygiene stations | No hand hygiene station design adaptation or a different type of adaptation among interventions to improve hand hygiene in community settings | Effective and sustained hand hygiene (i.e., consistent hand hygiene practices) | Randomized and non-randomized controlled trials, before-after studies (will consult any papers linked to the evaluation that may be relevant, e.g., protocols, follow-up studies (qual or quant) to assess sustainability) |
|  | (3.2f) Among interventions to improve hand hygiene in community settings, what level of frequency and intensity of behavior change interventions is necessary to effectively improve hand hygiene? | General population in community settings | Varying frequencies and intensities of behavior change interventions to promote effective hand hygiene | Standard frequency and intensity of behavior change interventions among interventions to improve hand hygiene in community settings | Effective and sustained hand hygiene (i.e., consistent hand hygiene practices) | Randomized and non-randomized controlled trials, before-after studies |
|  |  | **Sample** | **Phenomenon of interest** | **Design** | **Evaluation** | **Research type** |
|  | (3.2g) Among interventions to improve hand hygiene in community settings, how do hand hygiene practices vary by population groups, risk scenarios, or over time? | General population in community settings | Hand hygiene practices among key population groups and risk scenarios in community settings | Experimental or quasi-experimental design randomised and non-randomised controlled trials, before-after studies | Variations in hand hygiene practices | Mixed methods (focus on quantitative evaluation and will consult any papers linked to the evaluation that may be relevant, e.g., protocols, follow-up studies (qual or quant) to assess sustainability) |
