# Supplementary material for: Interventions to improve hand hygiene in community settings: a systematic review of theories, barriers and enablers, behaviour change techniques and hand hygiene station design features
Source: BMJ Glob Health. 2025 Sep 16;10(Suppl 7):e018928. doi: 10.1136/bmjgh-2025-018928 (PMC12443188; doi:10.1136/bmjgh-2025-018928)
Supplement: online supplemental file 4 [file bmjgh-10-Suppl_7-s004.docx]

**Interventions to improve hand hygiene in community settings: A systematic review of theories, barriers and enablers, behavior change techniques, and hand hygiene station design features**

*Authors*

Sridevi K. Prasad^1^ 0000-0003-0457-9534

Jedidiah S. Snyder^2^ 0000-0002-7688-4450

Erin LaFon^2^

Lilly A. O’Brien^2^ 0009-0004-1987-3706

Hannah Rogers^3^ 0000-0002-9515-1439

Oliver Cumming^4,5^ 0000-0002-5074-8709

Joanna Esteves Mills^5^

Bruce Gordon ^5^

Marlene Wolfe^2^ 0000-0002-6476-0450

Matthew C. Freeman^2^ 0000-0002-1517-2572

Bethany A. Caruso^1*^ 0000-0001-9738-9857

1 Hubert Department of Global Health, Rollins School of Public Health, Emory University, Atlanta, GA, USA; [bcaruso@emory.edu](mailto:bcaruso@emory.edu) (BAC); [sridevi.prasad@emory.edu](mailto:sridevi.prasad@emory.edu) (SKP)

2 Gangarosa Department of Environmental Health, Rollins School of Public Health, Emory University, Atlanta, GA, USA; [matthew.freeman@emory.edu](mailto:matthew.freeman@emory.edu) (MCF); [marlene.wolfe@emory.edu](mailto:marlene.wolfe@emory.edu) (MW) [jedidiah.snyder@emory.edu](mailto:jedidiah.snyder@emory.edu) (JSS); [lilly.obrien@emory.edu](mailto:lilly.obrien@emory.edu) (LAO); [erin.lafon@emory.edu](mailto:erin.lafon@emory.edu) (EL)

3 Woodruff Health Sciences Center Library, Emory University, Atlanta, GA, USA; [hannah.rogers@emory.edu](mailto:hannah.rogers@emory.edu) (HR)

4 Department of Disease Control, London School of Hygiene and Tropical Medicine, London, UK; [oliver.cumming@lshtm.ac.uk](mailto:oliver.cumming@lshtm.ac.uk) (OC)

5 Water, Sanitation, Hygiene and Health Unit, World Health Organization, Geneva, Switzerland; [estevesj@who.int](mailto:estevesj@who.int) (JEM); [gordonb@who.int](mailto:gordonb@who.int) (BG)

*Corresponding author: Bethany A. Caruso [bcaruso@emory.edu](mailto:bcaruso@emory.edu)

Emory University, Rollins School of Public Health, 1518 Clifton Rd, Atlanta, GA 30322

# Extraction fields for RQ3.2

| **#** | **Field** | **Details** | **Entry** | **Source** |
| --- | --- | --- | --- | --- |
| **1. Information about the extraction** | | | | |
| 1.1 | Reviewer ID | First and last initial (e.g., JS) | Text |  |
| 1.2 | Date of extraction | mm/dd/yy | Text |  |
| **2. Information about the study** | | | | |
| 2.1 | Author | Family name of first author | Text  999 = Not applicable |  |
| 2.2 | Publication year |  | Text  999 = Not applicable |  |
| 2.3 | Title | Title of study that data are extracted from | Text |  |
| 2.4 | Link | Paste the DOI or hyperlink to publication | Text  999 = Not applicable |  |
| 2.5 | Study type |  | *Select one*  (1) Journal article  (2) Grey literature (e.g., unpublished academic papers [e.g., thesis], non-peer reviewed papers, research and committee reports, government reports, conference papers/abstract, ongoing research)  (777) Other - specify |  |
| 2.6 | Study designc |  | *Select one*  (1) Descriptive (survey)  (2) Descriptive (qualitative)  (3) Descriptive (mixed methods)  (4) Randomized controlled trial  (5) Non-randomized control trial  (6) Quasi-experimental  (7) Case-control  (8) Cross-sectional  (9) Cohort  (10) Non-primary research  (777) Other – specify |  |
| 2.7 | Registered trial | Does the study report that it is linked to a registered trial? (e.g., clinicaltrials.gov, ICTRP) | *Select one*  (1) Yes  (0) No |  |
| 2.7.1 | Trial # | If linked to a registered trial, paste trial registration number | Text  (999) Not applicable |  |
| **3. Eligibility** | | | | |
| 3.1 | Confirm eligibility | Confirm eligible based on RQ criteria in PICO(S) or SPIDER format  DO NOT PROCEED IF ALL ARE NOT CHECKED | *Check multiple*   - Sample - The study includes general populations in community settings - PoI/Intervention - The study includes the correct PoIs (a-e) and/or interventions (f-g) of: (a) Behavior change theories among interventions to improve hand hygiene in community settings; (b) Effective leveraging of identified barriers and enablers of hand hygiene among interventions to improve hand hygiene in community settings; (c) Behavior change techniques to promote handwashing among interventions to improve hand hygiene in community settings; (d) Hand hygiene station design among interventions to improve hand hygiene in community settings; (e) Hand hygiene practices among key population groups and risk scenarios in community settings; (f) Design adaptations (e.g., placement, nudges, and cues) of hand hygiene stations; (g) Varying frequencies and intensities of behavior change interventions to promote effective hand hygiene - Design - The study is an experimental or quasi-experimental study, randomized controlled trial, non-randomized control trial, or before-after study - Evaluation - The study evaluates effective (and sustained) hand hygiene or variations in hand hygiene practices - Research type - The study uses qualitative, quantitative, and/or mixed methods | [Phase 2 criteria](https://emory-my.sharepoint.com/:w:/r/personal/bcaruso_emory_edu/Documents/Research/WHO/WHO-HW%20Systematic%20Reviews/1.%20WHO%20HW%20Systematic%20Reviews%20-%20project%20folder/3.%20Research%20activities/(3)%20Training%20documents/Phase%202%20training%20materials/Phase%202%20eligibility%20criteria.docx?d=w93c15eb4a94847adbb31c7248d059553&csf=1&web=1&e=Qc1U2s) |
| **4. Setting** | | | | |
| 4.1 | Country | Which country is represented in the study? (List all countries separated by a comma, if study is from multiple sites) | Text  (999) Not applicable |  |
| 4.2 | Region | Which region is represented in the study? | *Check multiple*  (1) Africa  (2) Asia  (3) Europe  (4) Latin America/Caribbean  (5) Middle East  (6) North America  (8) Oceania  (9) Multiple Regions  (10) Unspecified  (999) Not applicable | [Women’s Empowerment WASH SR](https://journals.plos.org/water/article?id=10.1371/journal.pwat.0000026) |
| 4.3 | Urban/Rural | Does the setting of the population fall under any of these specific categories? Select all that apply | *Check multiple*  (1) Urban  (2) Rural  (3) Peri-urban  (777) Other - specify  (888) Not reported  (999) Not applicable |  |
| 4.4 | Community setting | Does the setting of the population fall under any of these specific categories? Select all that apply | *Check multiple*  (1) Domestic - Households  (2) Public - Markets  (3) Public - Public transportation hubs  (4) Public - Parks, squares, or other public outdoor spaces,  (5) Institutions - Workplace  (6) Institutions - Schools  (7) Institutions - Universities  (8) Institutions - Places of worship  (9) Institutions - Prisons and places of detention  (10) Internally displaced people camps  (777) Other - specify  (888) Not reported  (999) Not applicable | [Macleod et al](https://bmjopen.bmj.com/content/13/6/e068887) |
| **5. Methods** | | | | |
| 5.1 | Aim of study | Paste the aim/objective/purpose/goal as stated in the study | Text  (888) Not reported  (999) Not applicable |  |
| 5.2 | Primary study outcome | What was the primary outcome for this study? | *Select one*  (1) Hand hygiene  (2) Diarrheal diseases  (3) Respiratory infections  (4) Influenza  (5) Infectious diseases  (6) Nutrition  (7) Mental/social well-being  (8) Neglected tropical diseases  (9) School absenteeism  (10) COVID-19  (11) Food hygiene  (777) Other – specify  (999) Not applicable |  |
| 5.3 | Start date | What is the study start date? Month, Year | Text  (888) Not reported |  |
| 5.4 | End date | What is the study end date? Month, Year | Text  (888) Not reported |  |
| **6. Participants** | | | | |
| 6.1 | Study participants | What is the group of people that researchers are examining in the study? Select all that apply | *Check multiple*  (1) General population  (2) Adults (Women and Men)  (3) Adults (Women only)  (4) Adults (Men only)  (5) Children (Girls and Boys)  (6) Children (Girls only)  (7) Children (Boys only)  (8) Mother-child dyads  (9) Food workers  (10) Non-food occupational workers  (777) Other – specify  (888) Not reported  (999) Not applicable |  |
| 6.2 | Vulnerable populations | Does study examine any of the following vulnerable populations?  Select all that apply | *Check multiple*  (1) Individuals with specific illness or risk factors  (2) Specific ethnic or religious groups  (3) Persons experiencing homelessness  (4) Persons with disabilities  (5) Immigrants and migrants  (6) Refugees and displaced persons  (7) Elderly  (8) Pregnant women  (777) Other - specify  (888) Not reported  (999) Not applicable |  |
| 6.3 | Number of participants | What is the total number of participants/sample size? | Text  (888) Not reported  (999) Not applicable |  |
| 6.3.1 | Sample size – Intervention arm 1 | What is the sample size for intervention arm/group 1? | Text  (888) Not reported  (999) Not applicable |  |
| 6.3.2 | Sample size – Intervention arm 2 | What is the sample size for intervention arm/group 2? | Text  (888) Not reported  (999) Not applicable |  |
| 6.3.3 | Sample size – Control arm | What is the sample size for control arm/group? | Text  (888) Not reported  (999) Not applicable |  |
| 6.4 | Age group | What is the age range in the eligibility criteria for participants? | Text (888) Not reported |  |
| 6.5 | Sex | What is the sex of the primary research population? | *Select one*  (1) Male  (2) Female  (3) Both male and female  (4) Unspecified |  |
| **7. Intervention** This section also addresses: **RQ3.2b Among interventions to improve hand hygiene in community settings, which have effectively leveraged identified barriers and enablers of hand hygiene in community settings? RQ3.2c Among interventions to improve hand hygiene in community settings, what behavior change techniques have been implemented to effectively improve and sustain handwashing practices?** | | | | |
| 7.1 | Protocol | In the methods, is there reference to a published study protocol? | (0) No  (1) Yes |  |
| 7.1.1 | Protocol citation | If published protocol, paste full citation | Citation  (999) Not applicable |  |
| 7.2 | Intervention | Does the study report that it is linked to an intervention? | (0) No  (1) Yes |  |
| 7.2.1 | Intervention name | If linked to an intervention, paste the name of the intervention | Text  (888) Not reported  (999) Not applicable |  |
| 7.2.2 | Intervention overall effectiveness | Is the intervention effective at achieving the primary outcome? (Note: effectiveness is defined by the authors) | *Select one*  (0) No  (1) Yes |  |
| 7.3 | Intervention aim behaviors | Did the intervention target other behaviors in addition to handwashing? | *Select one*  (0) No  (1) Yes |  |
| 7.4 | Multi-arm interventions | Does the study have multiple intervention arms that targeted hand hygiene? | (0) No  (1) Yes |  |
| 7.5 | Soap provision | Did the study provide **soap**? | *Select one*  (0) No  (1) Yes  (999) Not applicable |  |
| 7.5.1 | Intervention text | For this component, please copy in the author’s description. | Text  (888) Not reported  (999) Not applicable |  |
| 7.5.2 | Multi-arm | If the study is a multi-arm trial, which arm provided this intervention? | Text  (999) Not applicable |  |
| 7.5.3 | Explicit barrier | What barrier did the authors explicitly note that this intervention activity addressed? | Text  (888) Not noted  (999) Not applicable |  |
| 7.5.4 | Assumed barriers | If the barrier was not noted by the authors, what is the assumed barrier? | Text (999) Not applicable |  |
| 7.5.5 | Explicit enabler | What enabler did the authors explicitly note that this intervention activity addressed? | Text  (888) Not noted  (999) Not applicable |  |
| 7.5.6 | Assumed enabler | If the enabler was not noted by the authors, what is the assumed enabler? | Text (999) Not applicable |  |
| 7.5.7 | COM-B category | Which COM-B category was addressed through the intervention activity? | *Select multiple*  (1) Physical capability  (2) Psychological capability  (3) Physical opportunity  (4) Social opportunity  (5) Automatic motivation  (6) Reflective motivation  (999) Not applicable |  |
| 7.5.8 | Intervention function | Which COM-B intervention function maps to the behavior change technique above? | *Select multiple*  (1) Education  (2) Persuasion  (3) Incentivization  (4) Coercion  (5) Training  (6) Restriction  (7) Environmental Restructuring  (8) Modelling  (9) Enablement  (999) Not applicable |  |
| 7.5.9 | Behavior change technique – Goals and planning | Goals and planning: For this component, what behavior change techniques map to the intervention activity described above? | *Select multiple*  (1) Goal setting (behavior)  (2) Problem solving  (3) Goal setting (outcome)  (4) Action planning  (5) Review behavior goals  (6) Discrepancy between current behavior and goal  (7) Review outcome goals  (8) Behavioral contract  (9) Commitment  (999) Not applicable |  |
| 7.5.10 | Behavior change technique – Feedback and monitoring | Feedback and monitoring: For this component, what behavior change techniques map to the intervention activity described above? | *Select multiple*  (1) Monitoring of behavior by others without feedback  (2) Feedback on behavior  (3) Self-monitoring of behavior  (4) Self-monitoring of outcome(s) of behavior)  (5) Monitoring outcome(s) of behavior by others without feedback (6) Biofeedback (7) Feedback on outcome(s) of behavior  (999) Not applicable |  |
| 7.5.11 | Behavior change technique – Social support | Social support: For this component, what behavior change techniques map to the intervention activity described above? | *Select multiple*  (1) Social support (unspecified)  (2) Social support (practical)  (3) Social support (emotional)  (999) Not applicable |  |
| 7.5.12 | Behavior change technique – Shaping knowledge | Shaping knowledge: For this component, what behavior change techniques map to the intervention activity described above? | *Select multiple*  (1) Instruction on how to perform a behavior  (2) Information about antecedents  (3) Re-attribution  (4) Behavioral experiments  (999) Not applicable |  |
| 7.5.13 | Behavior change technique – Natural consequences | Natural consequences: For this component, what behavior change techniques map to the intervention activity described above? | *Select multiple*  (1) Information about health consequences  (2) Salience of consequences  (3) Information about social and environmental consequences (4) Monitoring of emotional consequences  (5) Anticipated regret  (6) Information about emotional consequences  (999) Not applicable |  |
| 7.5.14 | Behavior change technique – Comparison of behavior | Comparison of behavior: For this component, what behavior change techniques map to the intervention activity described above? | *Select multiple*  (1) Demonstration of behavior  (2) Social comparison  (3) Information about others’ approval  (999) Not applicable |  |
| 7.5.15 | Behavior change technique – Associations | Associations: For this component, what behavior change techniques map to the intervention activity described above? | *Select multiple*  (1) Prompts/cues  (2) Cue signaling reward  (3) Reduce prompts/cues  (4) Remove access to the reward  (5) Remove aversive stimulus  (6) Satiation  (7) Exposure  (8) Associative learning  (999) Not applicable |  |
| 7.5.16 | Behavior change technique – Repetition and substitution | Repetition and substitution: For this component, what behavior change techniques map to the intervention activity described above? | *Select multiple*  (1) Behavioral practice/rehearsal  (2) Behavior substitution  (3) Habit formation  (4) Habit reversal  (5) Overcorrection  (6) Generalization of a target behavior (7) Graded tasks  (999) Not applicable |  |
| 7.5.17 | Behavior change technique – Comparison of outcomes | Comparison of outcomes: For this component, what behavior change techniques map to the intervention activity described above? | *Select multiple*  (1) Credible source (2) Pros and cons (3) Comparative imagining of future outcomes  (999) Not applicable |  |
| 7.5.18 | Behavior change technique – Reward and threat | Reward and threat: For this component, what behavior change techniques map to the intervention activity described above? | *Select multiple*  (1) Material incentive (behavior)  (2) Material reward (behavior)  (3) Non-specific reward  (4) Social reward  (5) Social incentive  (6) Non-specific incentive  (7) Self-incentive  (8) Incentive (outcome)  (9) Self-reward  (10) Reward (outcome)  (11) Future punishment  (999) Not applicable |  |
| 7.5.19 | Behavior change technique – Regulation | Regulation: For this component, what behavior change techniques map to the intervention activity described above? | *Select multiple*  (1) Pharmocological support  (2) Reduce negative emotions  (3) Conserving mental resources  (4) Paradoxical instructions  (999) Not applicable |  |
| 7.5.20 | Behavior change technique – Antecedents | Antecedents: For this component, what behavior change techniques map to the intervention activity described above? | *Select multiple*  (1) Restructuring the physical environment  (2) Restructuring the social environment  (3) Avoidance/reducing exposure to cues for the behavior  (4) Distraction  (5) Adding objects to the environment  (6) Body changes  (999) Not applicable |  |
| 7.5.21 | Behavior change technique – Identity | Identity: For this component, what behavior change techniques map to the intervention activity described above? | *Select multiple*  (1) Identification of self as role model  (2) Framing/reframing  (3) Incompatible beliefs  (4) Valued self-identity  (5) Identity associated with changed behavior  (999) Not applicable |  |
| 7.5.22 | Behavior change technique – Scheduled consequences | Scheduled consequences: For this component, what behavior change techniques map to the intervention activity described above? | *Select multiple*  (1) Behavior cost  (2) Punishment  (3) Remove reward  (4) Reward approximation  (5) Rewarding completion  (6) Situation-specific reward  (7) Reward incompatible behavior  (8) Reward alternative behavior  (9) Reduce reward frequency  (10) Remove punishment  (999) Not applicable |  |
| 7.5.23 | Behavior change technique – Self-belief | Self-belief: For this component, what behavior change techniques map to the intervention activity described above? | *Select multiple*  (1) Verbal persuasion about capability  (2) Mental rehearsal of successful performance  (3) Focus on past success  (4) Self-talk  (999) Not applicable |  |
| 7.5.24 | Behavior change technique – Covert learning | Covert learning: For this component, what behavior change techniques map to the intervention activity described above? | *Select multiple*  (1) Imaginary punishment  (2) Imaginary reward  (3) Vicarious consequences  (999) Not applicable |  |
| 7.6 | Provision of alcohol rub/gel/hand sanitizer | Did the study provide **alcohol rub/gel/hand sanitizer?** | *Select one*  (0) No  (1) Yes  (999) Not applicable |  |
| 7.6.1 | Intervention text | For this component, please copy in the author’s description. | Text  (888) Not reported  (999) Not applicable |  |
| 7.6.2 | Multi-arm | If the study is a multi-arm trial, which arm provided this intervention? | Text  (999) Not applicable |  |
| 7.6.3 | Explicit barrier | What barrier did the authors explicitly note that this intervention activity addressed? | Text  (888) Not noted  (999) Not applicable |  |
| 7.6.4 | Assumed barriers | If the barrier was not noted by the authors, what is the assumed barrier? | Text (999) Not applicable |  |
| 7.6.5 | Explicit enabler | What enabler did the authors explicitly note that this intervention activity addressed? | Text  (888) Not noted  (999) Not applicable |  |
| 7.6.6 | Assumed enabler | If the enabler was not noted by the authors, what is the assumed enabler? | Text (999) Not applicable |  |
| 7.6.7 | COM-B category | Which COM-B category was addressed through the intervention activity? | *Select multiple*  (1) Physical capability  (2) Psychological capability  (3) Physical opportunity  (4) Social opportunity  (5) Automatic motivation  (6) Reflective motivation  (999) Not applicable |  |
| 7.6.8 | Intervention function | Which COM-B intervention function maps to the behavior change technique above? | *Select multiple*  (1) Education  (2) Persuasion  (3) Incentivization  (4) Coercion  (5) Training  (6) Restriction  (7) Environmental Restructuring  (8) Modelling  (9) Enablement  (999) Not applicable |  |
| 7.6.9 | Behavior change technique – Goals and planning | Goals and planning: For this component, what behavior change techniques map to the intervention activity described above? | *Select multiple*  (1) Goal setting (behavior)  (2) Problem solving  (3) Goal setting (outcome)  (4) Action planning  (5) Review behavior goals  (6) Discrepancy between current behavior and goal  (7) Review outcome goals  (8) Behavioral contract  (9) Commitment  (999) Not applicable |  |
| 7.6.10 | Behavior change technique – Feedback and monitoring | Feedback and monitoring: For this component, what behavior change techniques map to the intervention activity described above? | *Select multiple*  (1) Monitoring of behavior by others without feedback  (2) Feedback on behavior  (3) Self-monitoring of behavior  (4) Self-monitoring of outcome(s) of behavior)  (5) Monitoring outcome(s) of behavior by others without feedback (6) Biofeedback (7) Feedback on outcome(s) of behavior  (999) Not applicable |  |
| 7.6.11 | Behavior change technique – Social support | Social support: For this component, what behavior change techniques map to the intervention activity described above? | *Select multiple*  (1) Social support (unspecified)  (2) Social support (practical)  (3) Social support (emotional)  (999) Not applicable |  |
| 7.6.12 | Behavior change technique – Shaping knowledge | Shaping knowledge: For this component, what behavior change techniques map to the intervention activity described above? | *Select multiple*  (1) Instruction on how to perform a behavior  (2) Information about antecedents  (3) Re-attribution  (4) Behavioral experiments  (999) Not applicable |  |
| 7.6.13 | Behavior change technique – Natural consequences | Natural consequences: For this component, what behavior change techniques map to the intervention activity described above? | *Select multiple*  (1) Information about health consequences  (2) Salience of consequences  (3) Information about social and environmental consequences (4) Monitoring of emotional consequences  (5) Anticipated regret  (6) Information about emotional consequences  (999) Not applicable |  |
| 7.6.14 | Behavior change technique – Comparison of behavior | Comparison of behavior: For this component, what behavior change techniques map to the intervention activity described above? | *Select multiple*  (1) Demonstration of behavior  (2) Social comparison  (3) Information about others’ approval  (999) Not applicable |  |
| 7.6.15 | Behavior change technique – Associations | Associations: For this component, what behavior change techniques map to the intervention activity described above? | *Select multiple*  (1) Prompts/cues  (2) Cue signaling reward  (3) Reduce prompts/cues  (4) Remove access to the reward  (5) Remove aversive stimulus  (6) Satiation  (7) Exposure  (8) Associative learning  (999) Not applicable |  |
| 7.6.16 | Behavior change technique – Repetition and substitution | Repetition and substitution: For this component, what behavior change techniques map to the intervention activity described above? | *Select multiple*  (1) Behavioral practice/rehearsal  (2) Behavior substitution  (3) Habit formation  (4) Habit reversal  (5) Overcorrection  (6) Generalization of a target behavior (7) Graded tasks  (999) Not applicable |  |
| 7.6.17 | Behavior change technique – Comparison of outcomes | Comparison of outcomes: For this component, what behavior change techniques map to the intervention activity described above? | *Select multiple*  (1) Credible source (2) Pros and cons (3) Comparative imagining of future outcomes  (999) Not applicable |  |
| 7.6.18 | Behavior change technique – Reward and threat | Reward and threat: For this component, what behavior change techniques map to the intervention activity described above? | *Select multiple*  (1) Material incentive (behavior)  (2) Material reward (behavior)  (3) Non-specific reward  (4) Social reward  (5) Social incentive  (6) Non-specific incentive  (7) Self-incentive  (8) Incentive (outcome)  (9) Self-reward  (10) Reward (outcome)  (11) Future punishment  (999) Not applicable |  |
| 7.6.19 | Behavior change technique – Regulation | Regulation: For this component, what behavior change techniques map to the intervention activity described above? | *Select multiple*  (1) Pharmocological support  (2) Reduce negative emotions  (3) Conserving mental resources  (4) Paradoxical instructions  (999) Not applicable |  |
| 7.6.20 | Behavior change technique – Antecedents | Antecedents: For this component, what behavior change techniques map to the intervention activity described above? | *Select multiple*  (1) Restructuring the physical environment  (2) Restructuring the social environment  (3) Avoidance/reducing exposure to cues for the behavior  (4) Distraction  (5) Adding objects to the environment  (6) Body changes  (999) Not applicable |  |
| 7.6.21 | Behavior change technique – Identity | Identity: For this component, what behavior change techniques map to the intervention activity described above? | *Select multiple*  (1) Identification of self as role model  (2) Framing/reframing  (3) Incompatible beliefs  (4) Valued self-identity  (5) Identity associated with changed behavior  (999) Not applicable |  |
| 7.6.22 | Behavior change technique – Scheduled consequences | Scheduled consequences: For this component, what behavior change techniques map to the intervention activity described above? | *Select multiple*  (1) Behavior cost  (2) Punishment  (3) Remove reward  (4) Reward approximation  (5) Rewarding completion  (6) Situation-specific reward  (7) Reward incompatible behavior  (8) Reward alternative behavior  (9) Reduce reward frequency  (10) Remove punishment  (999) Not applicable |  |
| 7.6.23 | Behavior change technique – Self-belief | Self-belief: For this component, what behavior change techniques map to the intervention activity described above? | *Select multiple*  (1) Verbal persuasion about capability  (2) Mental rehearsal of successful performance  (3) Focus on past success  (4) Self-talk  (999) Not applicable |  |
| 7.6.24 | Behavior change technique – Covert learning | Covert learning: For this component, what behavior change techniques map to the intervention activity described above? | *Select multiple*  (1) Imaginary punishment  (2) Imaginary reward  (3) Vicarious consequences  (999) Not applicable |  |
| 7.7 | Installation of handwashing station | Did the study provide/install **a handwashing station?** | *Select one*  (0) No  (1) Yes  (999) Not applicable |  |
| 7.7.1 | Intervention text | For this component, please copy in the author’s description. | Text  (888) Not reported  (999) Not applicable |  |
| 7.7.2 | Multi-arm | If the study is a multi-arm trial, which arm provided this intervention? | Text  (999) Not applicable |  |
| 7.7.3 | Explicit barrier | What barrier did the authors explicitly note that this intervention activity addressed? | Text  (888) Not noted  (999) Not applicable |  |
| 7.7.4 | Assumed barriers | If the barrier was not noted by the authors, what is the assumed barrier? | Text (999) Not applicable |  |
| 7.7.5 | Explicit enabler | What enabler did the authors explicitly note that this intervention activity addressed? | Text  (888) Not noted  (999) Not applicable |  |
| 7.7.6 | Assumed enabler | If the enabler was not noted by the authors, what is the assumed enabler? | Text (999) Not applicable |  |
| 7.7.7 | COM-B category | Which COM-B category was addressed through the intervention activity? | *Select multiple*  (1) Physical capability  (2) Psychological capability  (3) Physical opportunity  (4) Social opportunity  (5) Automatic motivation  (6) Reflective motivation  (999) Not applicable |  |
| 7.7.8 | Intervention function | Which COM-B intervention function maps to the behavior change technique above? | *Select multiple*  (1) Education  (2) Persuasion  (3) Incentivization  (4) Coercion  (5) Training  (6) Restriction  (7) Environmental Restructuring  (8) Modelling  (9) Enablement  (999) Not applicable |  |
| 7.7.9 | Behavior change technique – Goals and planning | Goals and planning: For this component, what behavior change techniques map to the intervention activity described above? | *Select multiple*  (1) Goal setting (behavior)  (2) Problem solving  (3) Goal setting (outcome)  (4) Action planning  (5) Review behavior goals  (6) Discrepancy between current behavior and goal  (7) Review outcome goals  (8) Behavioral contract  (9) Commitment  (999) Not applicable |  |
| 7.7.10 | Behavior change technique – Feedback and monitoring | Feedback and monitoring: For this component, what behavior change techniques map to the intervention activity described above? | *Select multiple*  (1) Monitoring of behavior by others without feedback  (2) Feedback on behavior  (3) Self-monitoring of behavior  (4) Self-monitoring of outcome(s) of behavior)  (5) Monitoring outcome(s) of behavior by others without feedback (6) Biofeedback (7) Feedback on outcome(s) of behavior  (999) Not applicable |  |
| 7.7.11 | Behavior change technique – Social support | Social support: For this component, what behavior change techniques map to the intervention activity described above? | *Select multiple*  (1) Social support (unspecified)  (2) Social support (practical)  (3) Social support (emotional)  (999) Not applicable |  |
| 7.7.12 | Behavior change technique – Shaping knowledge | Shaping knowledge: For this component, what behavior change techniques map to the intervention activity described above? | *Select multiple*  (1) Instruction on how to perform a behavior  (2) Information about antecedents  (3) Re-attribution  (4) Behavioral experiments  (999) Not applicable |  |
| 7.7.13 | Behavior change technique – Natural consequences | Natural consequences: For this component, what behavior change techniques map to the intervention activity described above? | *Select multiple*  (1) Information about health consequences  (2) Salience of consequences  (3) Information about social and environmental consequences (4) Monitoring of emotional consequences  (5) Anticipated regret  (6) Information about emotional consequences  (999) Not applicable |  |
| 7.7.14 | Behavior change technique – Comparison of behavior | Comparison of behavior: For this component, what behavior change techniques map to the intervention activity described above? | *Select multiple*  (1) Demonstration of behavior  (2) Social comparison  (3) Information about others’ approval  (999) Not applicable |  |
| 7.7.15 | Behavior change technique – Associations | Associations: For this component, what behavior change techniques map to the intervention activity described above? | *Select multiple*  (1) Prompts/cues  (2) Cue signaling reward  (3) Reduce prompts/cues  (4) Remove access to the reward  (5) Remove aversive stimulus  (6) Satiation  (7) Exposure  (8) Associative learning  (999) Not applicable |  |
| 7.7.16 | Behavior change technique – Repetition and substitution | Repetition and substitution: For this component, what behavior change techniques map to the intervention activity described above? | *Select multiple*  (1) Behavioral practice/rehearsal  (2) Behavior substitution  (3) Habit formation  (4) Habit reversal  (5) Overcorrection  (6) Generalization of a target behavior (7) Graded tasks  (999) Not applicable |  |
| 7.7.17 | Behavior change technique – Comparison of outcomes | Comparison of outcomes: For this component, what behavior change techniques map to the intervention activity described above? | *Select multiple*  (1) Credible source (2) Pros and cons (3) Comparative imagining of future outcomes  (999) Not applicable |  |
| 7.7.18 | Behavior change technique – Reward and threat | Reward and threat: For this component, what behavior change techniques map to the intervention activity described above? | *Select multiple*  (1) Material incentive (behavior)  (2) Material reward (behavior)  (3) Non-specific reward  (4) Social reward  (5) Social incentive  (6) Non-specific incentive  (7) Self-incentive  (8) Incentive (outcome)  (9) Self-reward  (10) Reward (outcome)  (11) Future punishment  (999) Not applicable |  |
| 7.7.19 | Behavior change technique – Regulation | Regulation: For this component, what behavior change techniques map to the intervention activity described above? | *Select multiple*  (1) Pharmocological support  (2) Reduce negative emotions  (3) Conserving mental resources  (4) Paradoxical instructions  (999) Not applicable |  |
| 7.7.20 | Behavior change technique – Antecedents | Antecedents: For this component, what behavior change techniques map to the intervention activity described above? | *Select multiple*  (1) Restructuring the physical environment  (2) Restructuring the social environment  (3) Avoidance/reducing exposure to cues for the behavior  (4) Distraction  (5) Adding objects to the environment  (6) Body changes  (999) Not applicable |  |
| 7.7.21 | Behavior change technique – Identity | Identity: For this component, what behavior change techniques map to the intervention activity described above? | *Select multiple*  (1) Identification of self as role model  (2) Framing/reframing  (3) Incompatible beliefs  (4) Valued self-identity  (5) Identity associated with changed behavior  (999) Not applicable |  |
| 7.7.22 | Behavior change technique – Scheduled consequences | Scheduled consequences: For this component, what behavior change techniques map to the intervention activity described above? | *Select multiple*  (1) Behavior cost  (2) Punishment  (3) Remove reward  (4) Reward approximation  (5) Rewarding completion  (6) Situation-specific reward  (7) Reward incompatible behavior  (8) Reward alternative behavior  (9) Reduce reward frequency  (10) Remove punishment  (999) Not applicable |  |
| 7.7.23 | Behavior change technique – Self-belief | Self-belief: For this component, what behavior change techniques map to the intervention activity described above? | *Select multiple*  (1) Verbal persuasion about capability  (2) Mental rehearsal of successful performance  (3) Focus on past success  (4) Self-talk  (999) Not applicable |  |
| 7.7.24 | Behavior change technique – Covert learning | Covert learning: For this component, what behavior change techniques map to the intervention activity described above? | *Select multiple*  (1) Imaginary punishment  (2) Imaginary reward  (3) Vicarious consequences  (999) Not applicable |  |
| 7.8 | Education | Did the study implement an **education program on hand hygiene?** | *Select one*  (0) No  (1) Yes  (999) Not applicable |  |
| 7.8.1 | Intervention text | For this component, please copy in the author’s description. | Text  (888) Not reported  (999) Not applicable |  |
| 7.8.2 | Multi-arm | If the study is a multi-arm trial, which arm provided this intervention? | Text  (999) Not applicable |  |
| 7.8.3 | Explicit barrier | What barrier did the authors explicitly note that this intervention activity addressed? | Text  (888) Not noted  (999) Not applicable |  |
| 7.8.4 | Assumed barriers | If the barrier was not noted by the authors, what is the assumed barrier? | Text (999) Not applicable |  |
| 7.8.5 | Explicit enabler | What enabler did the authors explicitly note that this intervention activity addressed? | Text  (888) Not noted  (999) Not applicable |  |
| 7.8.6 | Assumed enabler | If the enabler was not noted by the authors, what is the assumed enabler? | Text (999) Not applicable |  |
| 7.8.7 | COM-B category | Which COM-B category was addressed through the intervention activity? | *Select multiple*  (1) Physical capability  (2) Psychological capability  (3) Physical opportunity  (4) Social opportunity  (5) Automatic motivation  (6) Reflective motivation  (999) Not applicable |  |
| 7.8.8 | Intervention function | Which COM-B intervention function maps to the behavior change technique above? | *Select multiple*  (1) Education  (2) Persuasion  (3) Incentivization  (4) Coercion  (5) Training  (6) Restriction  (7) Environmental Restructuring  (8) Modelling  (9) Enablement  (999) Not applicable |  |
| 7.8.9 | Behavior change technique – Goals and planning | Goals and planning: For this component, what behavior change techniques map to the intervention activity described above? | *Select multiple*  (1) Goal setting (behavior)  (2) Problem solving  (3) Goal setting (outcome)  (4) Action planning  (5) Review behavior goals  (6) Discrepancy between current behavior and goal  (7) Review outcome goals  (8) Behavioral contract  (9) Commitment  (999) Not applicable |  |
| 7.8.10 | Behavior change technique – Feedback and monitoring | Feedback and monitoring: For this component, what behavior change techniques map to the intervention activity described above? | *Select multiple*  (1) Monitoring of behavior by others without feedback  (2) Feedback on behavior  (3) Self-monitoring of behavior  (4) Self-monitoring of outcome(s) of behavior)  (5) Monitoring outcome(s) of behavior by others without feedback (6) Biofeedback (7) Feedback on outcome(s) of behavior  (999) Not applicable |  |
| 7.8.11 | Behavior change technique – Social support | Social support: For this component, what behavior change techniques map to the intervention activity described above? | *Select multiple*  (1) Social support (unspecified)  (2) Social support (practical)  (3) Social support (emotional)  (999) Not applicable |  |
| 7.8.12 | Behavior change technique – Shaping knowledge | Shaping knowledge: For this component, what behavior change techniques map to the intervention activity described above? | *Select multiple*  (1) Instruction on how to perform a behavior  (2) Information about antecedents  (3) Re-attribution  (4) Behavioral experiments  (999) Not applicable |  |
| 7.8.13 | Behavior change technique – Natural consequences | Natural consequences: For this component, what behavior change techniques map to the intervention activity described above? | *Select multiple*  (1) Information about health consequences  (2) Salience of consequences  (3) Information about social and environmental consequences (4) Monitoring of emotional consequences  (5) Anticipated regret  (6) Information about emotional consequences  (999) Not applicable |  |
| 7.8.14 | Behavior change technique – Comparison of behavior | Comparison of behavior: For this component, what behavior change techniques map to the intervention activity described above? | *Select multiple*  (1) Demonstration of behavior  (2) Social comparison  (3) Information about others’ approval  (999) Not applicable |  |
| 7.8.15 | Behavior change technique – Associations | Associations: For this component, what behavior change techniques map to the intervention activity described above? | *Select multiple*  (1) Prompts/cues  (2) Cue signaling reward  (3) Reduce prompts/cues  (4) Remove access to the reward  (5) Remove aversive stimulus  (6) Satiation  (7) Exposure  (8) Associative learning  (999) Not applicable |  |
| 7.8.16 | Behavior change technique – Repetition and substitution | Repetition and substitution: For this component, what behavior change techniques map to the intervention activity described above? | *Select multiple*  (1) Behavioral practice/rehearsal  (2) Behavior substitution  (3) Habit formation  (4) Habit reversal  (5) Overcorrection  (6) Generalization of a target behavior (7) Graded tasks  (999) Not applicable |  |
| 7.8.17 | Behavior change technique – Comparison of outcomes | Comparison of outcomes: For this component, what behavior change techniques map to the intervention activity described above? | *Select multiple*  (1) Credible source (2) Pros and cons (3) Comparative imagining of future outcomes  (999) Not applicable |  |
| 7.8.18 | Behavior change technique – Reward and threat | Reward and threat: For this component, what behavior change techniques map to the intervention activity described above? | *Select multiple*  (1) Material incentive (behavior)  (2) Material reward (behavior)  (3) Non-specific reward  (4) Social reward  (5) Social incentive  (6) Non-specific incentive  (7) Self-incentive  (8) Incentive (outcome)  (9) Self-reward  (10) Reward (outcome)  (11) Future punishment  (999) Not applicable |  |
| 7.8.19 | Behavior change technique – Regulation | Regulation: For this component, what behavior change techniques map to the intervention activity described above? | *Select multiple*  (1) Pharmocological support  (2) Reduce negative emotions  (3) Conserving mental resources  (4) Paradoxical instructions  (999) Not applicable |  |
| 7.8.20 | Behavior change technique – Antecedents | Antecedents: For this component, what behavior change techniques map to the intervention activity described above? | *Select multiple*  (1) Restructuring the physical environment  (2) Restructuring the social environment  (3) Avoidance/reducing exposure to cues for the behavior  (4) Distraction  (5) Adding objects to the environment  (6) Body changes  (999) Not applicable |  |
| 7.8.21 | Behavior change technique – Identity | Identity: For this component, what behavior change techniques map to the intervention activity described above? | *Select multiple*  (1) Identification of self as role model  (2) Framing/reframing  (3) Incompatible beliefs  (4) Valued self-identity  (5) Identity associated with changed behavior  (999) Not applicable |  |
| 7.8.22 | Behavior change technique – Scheduled consequences | Scheduled consequences: For this component, what behavior change techniques map to the intervention activity described above? | *Select multiple*  (1) Behavior cost  (2) Punishment  (3) Remove reward  (4) Reward approximation  (5) Rewarding completion  (6) Situation-specific reward  (7) Reward incompatible behavior  (8) Reward alternative behavior  (9) Reduce reward frequency  (10) Remove punishment  (999) Not applicable |  |
| 7.8.23 | Behavior change technique – Self-belief | Self-belief: For this component, what behavior change techniques map to the intervention activity described above? | *Select multiple*  (1) Verbal persuasion about capability  (2) Mental rehearsal of successful performance  (3) Focus on past success  (4) Self-talk  (999) Not applicable |  |
| 7.8.24 | Behavior change technique – Covert learning | Covert learning: For this component, what behavior change techniques map to the intervention activity described above? | *Select multiple*  (1) Imaginary punishment  (2) Imaginary reward  (3) Vicarious consequences  (999) Not applicable |  |
| 7.9 | Training | Did the study implement a **training program on hand hygiene?** | *Select one*  (0) No  (1) Yes  (999) Not applicable |  |
| 7.9.1 | Intervention text | For this component, please copy in the author’s description. | Text  (888) Not reported  (999) Not applicable |  |
| 7.9.2 | Multi-arm | If the study is a multi-arm trial, which arm provided this intervention? | Text  (999) Not applicable |  |
| 7.9.3 | Explicit barrier | What barrier did the authors explicitly note that this intervention activity addressed? | Text  (888) Not noted  (999) Not applicable |  |
| 7.9.4 | Assumed barriers | If the barrier was not noted by the authors, what is the assumed barrier? | Text (999) Not applicable |  |
| 7.9.5 | Explicit enabler | What enabler did the authors explicitly note that this intervention activity addressed? | Text  (888) Not noted  (999) Not applicable |  |
| 7.9.6 | Assumed enabler | If the enabler was not noted by the authors, what is the assumed enabler? | Text (999) Not applicable |  |
| 7.9.7 | COM-B category | Which COM-B category was addressed through the intervention activity? | *Select multiple*  (1) Physical capability  (2) Psychological capability  (3) Physical opportunity  (4) Social opportunity  (5) Automatic motivation  (6) Reflective motivation  (999) Not applicable |  |
| 7.9.8 | Intervention function | Which COM-B intervention function maps to the behavior change technique above? | *Select multiple*  (1) Education  (2) Persuasion  (3) Incentivization  (4) Coercion  (5) Training  (6) Restriction  (7) Environmental Restructuring  (8) Modelling  (9) Enablement  (999) Not applicable |  |
| 7.9.9 | Behavior change technique – Goals and planning | Goals and planning: For this component, what behavior change techniques map to the intervention activity described above? | *Select multiple*  (1) Goal setting (behavior)  (2) Problem solving  (3) Goal setting (outcome)  (4) Action planning  (5) Review behavior goals  (6) Discrepancy between current behavior and goal  (7) Review outcome goals  (8) Behavioral contract  (9) Commitment  (999) Not applicable |  |
| 7.9.10 | Behavior change technique – Feedback and monitoring | Feedback and monitoring: For this component, what behavior change techniques map to the intervention activity described above? | *Select multiple*  (1) Monitoring of behavior by others without feedback  (2) Feedback on behavior  (3) Self-monitoring of behavior  (4) Self-monitoring of outcome(s) of behavior)  (5) Monitoring outcome(s) of behavior by others without feedback (6) Biofeedback (7) Feedback on outcome(s) of behavior  (999) Not applicable |  |
| 7.9.11 | Behavior change technique – Social support | Social support: For this component, what behavior change techniques map to the intervention activity described above? | *Select multiple*  (1) Social support (unspecified)  (2) Social support (practical)  (3) Social support (emotional)  (999) Not applicable |  |
| 7.9.12 | Behavior change technique – Shaping knowledge | Shaping knowledge: For this component, what behavior change techniques map to the intervention activity described above? | *Select multiple*  (1) Instruction on how to perform a behavior  (2) Information about antecedents  (3) Re-attribution  (4) Behavioral experiments  (999) Not applicable |  |
| 7.9.13 | Behavior change technique – Natural consequences | Natural consequences: For this component, what behavior change techniques map to the intervention activity described above? | *Select multiple*  (1) Information about health consequences  (2) Salience of consequences  (3) Information about social and environmental consequences (4) Monitoring of emotional consequences  (5) Anticipated regret  (6) Information about emotional consequences  (999) Not applicable |  |
| 7.9.14 | Behavior change technique – Comparison of behavior | Comparison of behavior: For this component, what behavior change techniques map to the intervention activity described above? | *Select multiple*  (1) Demonstration of behavior  (2) Social comparison  (3) Information about others’ approval  (999) Not applicable |  |
| 7.9.15 | Behavior change technique – Associations | Associations: For this component, what behavior change techniques map to the intervention activity described above? | *Select multiple*  (1) Prompts/cues  (2) Cue signaling reward  (3) Reduce prompts/cues  (4) Remove access to the reward  (5) Remove aversive stimulus  (6) Satiation  (7) Exposure  (8) Associative learning  (999) Not applicable |  |
| 7.9.16 | Behavior change technique – Repetition and substitution | Repetition and substitution: For this component, what behavior change techniques map to the intervention activity described above? | *Select multiple*  (1) Behavioral practice/rehearsal  (2) Behavior substitution  (3) Habit formation  (4) Habit reversal  (5) Overcorrection  (6) Generalization of a target behavior (7) Graded tasks  (999) Not applicable |  |
| 7.9.17 | Behavior change technique – Comparison of outcomes | Comparison of outcomes: For this component, what behavior change techniques map to the intervention activity described above? | *Select multiple*  (1) Credible source (2) Pros and cons (3) Comparative imagining of future outcomes  (999) Not applicable |  |
| 7.9.18 | Behavior change technique – Reward and threat | Reward and threat: For this component, what behavior change techniques map to the intervention activity described above? | *Select multiple*  (1) Material incentive (behavior)  (2) Material reward (behavior)  (3) Non-specific reward  (4) Social reward  (5) Social incentive  (6) Non-specific incentive  (7) Self-incentive  (8) Incentive (outcome)  (9) Self-reward  (10) Reward (outcome)  (11) Future punishment  (999) Not applicable |  |
| 7.9.19 | Behavior change technique – Regulation | Regulation: For this component, what behavior change techniques map to the intervention activity described above? | *Select multiple*  (1) Pharmocological support  (2) Reduce negative emotions  (3) Conserving mental resources  (4) Paradoxical instructions  (999) Not applicable |  |
| 7.9.20 | Behavior change technique – Antecedents | Antecedents: For this component, what behavior change techniques map to the intervention activity described above? | *Select multiple*  (1) Restructuring the physical environment  (2) Restructuring the social environment  (3) Avoidance/reducing exposure to cues for the behavior  (4) Distraction  (5) Adding objects to the environment  (6) Body changes  (999) Not applicable |  |
| 7.9.21 | Behavior change technique – Identity | Identity: For this component, what behavior change techniques map to the intervention activity described above? | *Select multiple*  (1) Identification of self as role model  (2) Framing/reframing  (3) Incompatible beliefs  (4) Valued self-identity  (5) Identity associated with changed behavior  (999) Not applicable |  |
| 7.9.22 | Behavior change technique – Scheduled consequences | Scheduled consequences: For this component, what behavior change techniques map to the intervention activity described above? | *Select multiple*  (1) Behavior cost  (2) Punishment  (3) Remove reward  (4) Reward approximation  (5) Rewarding completion  (6) Situation-specific reward  (7) Reward incompatible behavior  (8) Reward alternative behavior  (9) Reduce reward frequency  (10) Remove punishment  (999) Not applicable |  |
| 7.9.23 | Behavior change technique – Self-belief | Self-belief: For this component, what behavior change techniques map to the intervention activity described above? | *Select multiple*  (1) Verbal persuasion about capability  (2) Mental rehearsal of successful performance  (3) Focus on past success  (4) Self-talk  (999) Not applicable |  |
| 7.9.24 | Behavior change technique – Covert learning | Covert learning: For this component, what behavior change techniques map to the intervention activity described above? | *Select multiple*  (1) Imaginary punishment  (2) Imaginary reward  (3) Vicarious consequences  (999) Not applicable |  |
| 7.10 | Multimedia messaging | Did the study use **multimedia messaging (posters, film/videos, pamphlets, etc.)** to promote hand hygiene? | *Select one*  (0)  (1) Yes  (999) Not applicable |  |
| 7.10.1 | Intervention text | For this component, please copy in the author’s description. | Text  (888) Not reported  (999) Not applicable |  |
| 7.10.2 | Multi-arm | If the study is a multi-arm trial, which arm provided this intervention? | Text  (999) Not applicable |  |
| 7.10.3 | Explicit barrier | What barrier did the authors explicitly note that this intervention activity addressed? | Text  (888) Not noted  (999) Not applicable |  |
| 7.10.4 | Assumed barriers | If the barrier was not noted by the authors, what is the assumed barrier? | Text (999) Not applicable |  |
| 7.10.5 | Explicit enabler | What enabler did the authors explicitly note that this intervention activity addressed? | Text  (888) Not noted  (999) Not applicable |  |
| 7.10.6 | Assumed enabler | If the enabler was not noted by the authors, what is the assumed enabler? | Text (999) Not applicable |  |
| 7.10.7 | COM-B category | Which COM-B category was addressed through the intervention activity? | *Select multiple*  (1) Physical capability  (2) Psychological capability  (3) Physical opportunity  (4) Social opportunity  (5) Automatic motivation  (6) Reflective motivation  (999) Not applicable |  |
| 7.10.8 | Intervention function | Which COM-B intervention function maps to the behavior change technique above? | *Select multiple*  (1) Education  (2) Persuasion  (3) Incentivization  (4) Coercion  (5) Training  (6) Restriction  (7) Environmental Restructuring  (8) Modelling  (9) Enablement  (999) Not applicable |  |
| 7.10.9 | Behavior change technique – Goals and planning | Goals and planning: For this component, what behavior change techniques map to the intervention activity described above? | *Select multiple*  (1) Goal setting (behavior)  (2) Problem solving  (3) Goal setting (outcome)  (4) Action planning  (5) Review behavior goals  (6) Discrepancy between current behavior and goal  (7) Review outcome goals  (8) Behavioral contract  (9) Commitment  (999) Not applicable |  |
| 7.10.10 | Behavior change technique – Feedback and monitoring | Feedback and monitoring: For this component, what behavior change techniques map to the intervention activity described above? | *Select multiple*  (1) Monitoring of behavior by others without feedback  (2) Feedback on behavior  (3) Self-monitoring of behavior  (4) Self-monitoring of outcome(s) of behavior)  (5) Monitoring outcome(s) of behavior by others without feedback (6) Biofeedback (7) Feedback on outcome(s) of behavior  (999) Not applicable |  |
| 7.10.11 | Behavior change technique – Social support | Social support: For this component, what behavior change techniques map to the intervention activity described above? | *Select multiple*  (1) Social support (unspecified)  (2) Social support (practical)  (3) Social support (emotional)  (999) Not applicable |  |
| 7.10.12 | Behavior change technique – Shaping knowledge | Shaping knowledge: For this component, what behavior change techniques map to the intervention activity described above? | *Select multiple*  (1) Instruction on how to perform a behavior  (2) Information about antecedents  (3) Re-attribution  (4) Behavioral experiments  (999) Not applicable |  |
| 7.10.13 | Behavior change technique – Natural consequences | Natural consequences: For this component, what behavior change techniques map to the intervention activity described above? | *Select multiple*  (1) Information about health consequences  (2) Salience of consequences  (3) Information about social and environmental consequences (4) Monitoring of emotional consequences  (5) Anticipated regret  (6) Information about emotional consequences  (999) Not applicable |  |
| 7.10.14 | Behavior change technique – Comparison of behavior | Comparison of behavior: For this component, what behavior change techniques map to the intervention activity described above? | *Select multiple*  (1) Demonstration of behavior  (2) Social comparison  (3) Information about others’ approval  (999) Not applicable |  |
| 7.10.15 | Behavior change technique – Associations | Associations: For this component, what behavior change techniques map to the intervention activity described above? | *Select multiple*  (1) Prompts/cues  (2) Cue signaling reward  (3) Reduce prompts/cues  (4) Remove access to the reward  (5) Remove aversive stimulus  (6) Satiation  (7) Exposure  (8) Associative learning  (999) Not applicable |  |
| 7.10.16 | Behavior change technique – Repetition and substitution | Repetition and substitution: For this component, what behavior change techniques map to the intervention activity described above? | *Select multiple*  (1) Behavioral practice/rehearsal  (2) Behavior substitution  (3) Habit formation  (4) Habit reversal  (5) Overcorrection  (6) Generalization of a target behavior (7) Graded tasks  (999) Not applicable |  |
| 7.10.17 | Behavior change technique – Comparison of outcomes | Comparison of outcomes: For this component, what behavior change techniques map to the intervention activity described above? | *Select multiple*  (1) Credible source (2) Pros and cons (3) Comparative imagining of future outcomes  (999) Not applicable |  |
| 7.10.18 | Behavior change technique – Reward and threat | Reward and threat: For this component, what behavior change techniques map to the intervention activity described above? | *Select multiple*  (1) Material incentive (behavior)  (2) Material reward (behavior)  (3) Non-specific reward  (4) Social reward  (5) Social incentive  (6) Non-specific incentive  (7) Self-incentive  (8) Incentive (outcome)  (9) Self-reward  (10) Reward (outcome)  (11) Future punishment  (999) Not applicable |  |
| 7.10.19 | Behavior change technique – Regulation | Regulation: For this component, what behavior change techniques map to the intervention activity described above? | *Select multiple*  (1) Pharmocological support  (2) Reduce negative emotions  (3) Conserving mental resources  (4) Paradoxical instructions  (999) Not applicable |  |
| 7.10.20 | Behavior change technique – Antecedents | Antecedents: For this component, what behavior change techniques map to the intervention activity described above? | *Select multiple*  (1) Restructuring the physical environment  (2) Restructuring the social environment  (3) Avoidance/reducing exposure to cues for the behavior  (4) Distraction  (5) Adding objects to the environment  (6) Body changes  (999) Not applicable |  |
| 7.10.21 | Behavior change technique – Identity | Identity: For this component, what behavior change techniques map to the intervention activity described above? | *Select multiple*  (1) Identification of self as role model  (2) Framing/reframing  (3) Incompatible beliefs  (4) Valued self-identity  (5) Identity associated with changed behavior  (999) Not applicable |  |
| 7.10.22 | Behavior change technique – Scheduled consequences | Scheduled consequences: For this component, what behavior change techniques map to the intervention activity described above? | *Select multiple*  (1) Behavior cost  (2) Punishment  (3) Remove reward  (4) Reward approximation  (5) Rewarding completion  (6) Situation-specific reward  (7) Reward incompatible behavior  (8) Reward alternative behavior  (9) Reduce reward frequency  (10) Remove punishment  (999) Not applicable |  |
| 7.10.23 | Behavior change technique – Self-belief | Self-belief: For this component, what behavior change techniques map to the intervention activity described above? | *Select multiple*  (1) Verbal persuasion about capability  (2) Mental rehearsal of successful performance  (3) Focus on past success  (4) Self-talk  (999) Not applicable |  |
| 7.10.24 | Behavior change technique – Covert learning | Covert learning: For this component, what behavior change techniques map to the intervention activity described above? | *Select multiple*  (1) Imaginary punishment  (2) Imaginary reward  (3) Vicarious consequences  (999) Not applicable |  |
| 7.11 | Other intervention | Did the study implement another hand hygiene intervention not described above? | *Select one*  (0) No  (1) Yes  (999) Not applicable |  |
| 7.11.1 | Intervention text | For this component, please copy in the author’s description. | Text  (888) Not reported  (999) Not applicable |  |
| 7.11.2 | Multi-arm | If the study is a multi-arm trial, which arm provided this intervention? | Text  (999) Not applicable |  |
| 7.11.3 | Explicit barrier | What barrier did the authors explicitly note that this intervention activity addressed? | Text  (888) Not noted  (999) Not applicable |  |
| 7.11.4 | Assumed barriers | If the barrier was not noted by the authors, what is the assumed barrier? | Text (999) Not applicable |  |
| 7.11.5 | Explicit enabler | What enabler did the authors explicitly note that this intervention activity addressed? | Text  (888) Not noted  (999) Not applicable |  |
| 7.11.6 | Assumed enabler | If the enabler was not noted by the authors, what is the assumed enabler? | Text (999) Not applicable |  |
| 7.11.7 | COM-B category | Which COM-B category was addressed through the intervention activity? | *Select multiple*  (1) Physical capability  (2) Psychological capability  (3) Physical opportunity  (4) Social opportunity  (5) Automatic motivation  (6) Reflective motivation  (999) Not applicable |  |
| 7.11.8 | Intervention function | Which COM-B intervention function maps to the behavior change technique above? | *Select multiple*  (1) Education  (2) Persuasion  (3) Incentivization  (4) Coercion  (5) Training  (6) Restriction  (7) Environmental Restructuring  (8) Modelling  (9) Enablement  (999) Not applicable |  |
| 7.11.9 | Behavior change technique – Goals and planning | Goals and planning: For this component, what behavior change techniques map to the intervention activity described above? | *Select multiple*  (1) Goal setting (behavior)  (2) Problem solving  (3) Goal setting (outcome)  (4) Action planning  (5) Review behavior goals  (6) Discrepancy between current behavior and goal  (7) Review outcome goals  (8) Behavioral contract  (9) Commitment  (999) Not applicable |  |
| 7.11.10 | Behavior change technique – Feedback and monitoring | Feedback and monitoring: For this component, what behavior change techniques map to the intervention activity described above? | *Select multiple*  (1) Monitoring of behavior by others without feedback  (2) Feedback on behavior  (3) Self-monitoring of behavior  (4) Self-monitoring of outcome(s) of behavior)  (5) Monitoring outcome(s) of behavior by others without feedback (6) Biofeedback (7) Feedback on outcome(s) of behavior  (999) Not applicable |  |
| 7.11.11 | Behavior change technique – Social support | Social support: For this component, what behavior change techniques map to the intervention activity described above? | *Select multiple*  (1) Social support (unspecified)  (2) Social support (practical)  (3) Social support (emotional)  (999) Not applicable |  |
| 7.11.12 | Behavior change technique – Shaping knowledge | Shaping knowledge: For this component, what behavior change techniques map to the intervention activity described above? | *Select multiple*  (1) Instruction on how to perform a behavior  (2) Information about antecedents  (3) Re-attribution  (4) Behavioral experiments  (999) Not applicable |  |
| 7.11.13 | Behavior change technique – Natural consequences | Natural consequences: For this component, what behavior change techniques map to the intervention activity described above? | *Select multiple*  (1) Information about health consequences  (2) Salience of consequences  (3) Information about social and environmental consequences (4) Monitoring of emotional consequences  (5) Anticipated regret  (6) Information about emotional consequences  (999) Not applicable |  |
| 7.11.14 | Behavior change technique – Comparison of behavior | Comparison of behavior: For this component, what behavior change techniques map to the intervention activity described above? | *Select multiple*  (1) Demonstration of behavior  (2) Social comparison  (3) Information about others’ approval  (999) Not applicable |  |
| 7.11.15 | Behavior change technique – Associations | Associations: For this component, what behavior change techniques map to the intervention activity described above? | *Select multiple*  (1) Prompts/cues  (2) Cue signaling reward  (3) Reduce prompts/cues  (4) Remove access to the reward  (5) Remove aversive stimulus  (6) Satiation  (7) Exposure  (8) Associative learning  (999) Not applicable |  |
| 7.11.16 | Behavior change technique – Repetition and substitution | Repetition and substitution: For this component, what behavior change techniques map to the intervention activity described above? | *Select multiple*  (1) Behavioral practice/rehearsal  (2) Behavior substitution  (3) Habit formation  (4) Habit reversal  (5) Overcorrection  (6) Generalization of a target behavior (7) Graded tasks  (999) Not applicable |  |
| 7.11.17 | Behavior change technique – Comparison of outcomes | Comparison of outcomes: For this component, what behavior change techniques map to the intervention activity described above? | *Select multiple*  (1) Credible source (2) Pros and cons (3) Comparative imagining of future outcomes  (999) Not applicable |  |
| 7.11.18 | Behavior change technique – Reward and threat | Reward and threat: For this component, what behavior change techniques map to the intervention activity described above? | *Select multiple*  (1) Material incentive (behavior)  (2) Material reward (behavior)  (3) Non-specific reward  (4) Social reward  (5) Social incentive  (6) Non-specific incentive  (7) Self-incentive  (8) Incentive (outcome)  (9) Self-reward  (10) Reward (outcome)  (11) Future punishment  (999) Not applicable |  |
| 7.11.19 | Behavior change technique – Regulation | Regulation: For this component, what behavior change techniques map to the intervention activity described above? | *Select multiple*  (1) Pharmocological support  (2) Reduce negative emotions  (3) Conserving mental resources  (4) Paradoxical instructions  (999) Not applicable |  |
| 7.11.20 | Behavior change technique – Antecedents | Antecedents: For this component, what behavior change techniques map to the intervention activity described above? | *Select multiple*  (1) Restructuring the physical environment  (2) Restructuring the social environment  (3) Avoidance/reducing exposure to cues for the behavior  (4) Distraction  (5) Adding objects to the environment  (6) Body changes  (999) Not applicable |  |
| 7.11.21 | Behavior change technique – Identity | Identity: For this component, what behavior change techniques map to the intervention activity described above? | *Select multiple*  (1) Identification of self as role model  (2) Framing/reframing  (3) Incompatible beliefs  (4) Valued self-identity  (5) Identity associated with changed behavior  (999) Not applicable |  |
| 7.11.22 | Behavior change technique – Scheduled consequences | Scheduled consequences: For this component, what behavior change techniques map to the intervention activity described above? | *Select multiple*  (1) Behavior cost  (2) Punishment  (3) Remove reward  (4) Reward approximation  (5) Rewarding completion  (6) Situation-specific reward  (7) Reward incompatible behavior  (8) Reward alternative behavior  (9) Reduce reward frequency  (10) Remove punishment  (999) Not applicable |  |
| 7.11.23 | Behavior change technique – Self-belief | Self-belief: For this component, what behavior change techniques map to the intervention activity described above? | *Select multiple*  (1) Verbal persuasion about capability  (2) Mental rehearsal of successful performance  (3) Focus on past success  (4) Self-talk  (999) Not applicable |  |
| 7.11.24 | Behavior change technique – Covert learning | Covert learning: For this component, what behavior change techniques map to the intervention activity described above? | *Select multiple*  (1) Imaginary punishment  (2) Imaginary reward  (3) Vicarious consequences  (999) Not applicable |  |
| **8. Hand Hygiene** | | | | |
| 8.1 | Practice | Type of practice (which can still be check multiple) | *Check multiple*  (1) Handwashing with soap and water  (2) Handwashing with water  (3) Handwashing with alcohol-based hand rub  (4) Handwashing with non-alcoholic antiseptics  (5) Handwashing with soap alternatives (e.g., ash)  (6) Handwashing - unspecified  (7) Hand drying  (777) Other – specify  (888) Not reported  (999) Not applicable |  |
| 8.2 | Outcome category | What category does this outcome fall into? | *Select one*  (1) Frequency of handwashing  (2) Efficacy of handwashing  (3) Consistency of handwashing  (777) Other - specify |  |
| 8.3 | Key moment | What key moment was this outcome looking at? | *Select one*  (1) Before, during, and after preparing food  (2) Before and after eating food (3) Before and after caring for someone who is sick with vomiting or diarrhea  (4) Before and after treating a cut or wound  (5) Before feeding a child  (6) After using the toilet  (7) After changing diapers/cleaning a child’s bottom  (8) After blowing your nose, coughing or sneezing  (9) After touching an animal, animal feed, or animal waste  (10) After touching garbage  (11) After handling pet food or pet treats  (12) Key moments index – combination (13) No specified key moment  (777) Other - specify |  |
| 8.4 | Participants | Copy in the population that’s measured by the outcome | Text  (999) Not applicable |  |
| 8.5 | Study arm | If effect estimate is reported separately by study arm, indicate which arm is below. | Text  (999) Not applicable |  |
| 8.6 | Measurement | How was this outcome measured? | *Select one*  (1) Direct observation  (2) Self-reported  (3) Proxy indicator  (888) Not reported  (999) Not applicable |  |
| 8.6.1 | Direct observation indicator | Paste in the indicator/outcome description that authors used for measurement. | Text  (999) Not applicable |  |
| 8.6.2 | Self-report | If hand hygiene is assessed by self-reporting, paste the survey question used | Text  (999) Not applicable |  |
| 8.6.3 | Proxy indicator | If hand hygiene is assessed by proxy indicator, paste the indicator used | Text  (999) Not applicable |  |
| 8.6.4 | Sex disaggregation | Was the hand hygiene practice(s) above disaggregated by sex? | *Select one*  (0) No  (1) Yes  (999) Not applicable |  |
| 8.6.5 | Effectiveness | Did the intervention have an impact on this outcome? (Note: Effectiveness is determined by study authors’ determination) | *Select one*  (0) No  (1) Yes  (888) Not reported  (999) Not applicable |  |
| 8.7.1 | Intervention group observations | What is the number of observations in the intervention group for this outcome? | Text  (888) Not reported  (999) Not applicable |  |
| 8.7.2 | Additional intervention group observations | What is the number of observations in the additional intervention group for this outcome? | Text  (888) Not reported  (999) Not applicable |  |
| 8.7.3 | Control group observations | What is the number of observations in the control group for this outcome? | Text  (888) Not reported  (999) Not applicable |  |
| 8.8.1 | Effect estimate | What is the effect estimate of hand hygiene practice? | Text  (888) Not reported  (999) Not applicable |  |
| 8.8.2 | Effect measure type | What type of effect measure is provided above? | *Select one*  (1) Risk ratio  (2) Odds ratio  (3) Prevalence ratio  (4) Rate ratio  (5) Mean difference  (6) Risk difference  (7) Proportion  (8) Number of events  (9) Percentage  (777) Other – specify  (999) Not applicable |  |
| 8.8.3 | Adjusted | Was the effect measure adjusted? | *Select one*  (1) No  (2) Yes  (888) Not reported  (999) Not applicable |  |
| 8.8.4 | Units | Copy in the units used for the effect (i.e. percentage points, number of events, etc.) | Text  (888) Not reported  (999) Not applicable |  |
| 8.8.5 | Lower CI | What is the lower confidence interval bound? | Text  (888) Not reported (999) Not applicable |  |
| 8.8.6 | Upper CI | What is the upper confidence interval bound? | Text  (888) Not reported (999) Not applicable |  |
| 8.8.7 | p-value | What is the p-value? | Text  (888) Not reported (999) Not applicable |  |
| 8.8.8 | Standard deviation | What is the standard deviation? | Text  (888) Not reported (999) Not applicable |  |
| **RQ3.2a Among interventions to improve hand hygiene in community settings, which have been designed using behavior change theories?** | | | | |
| 1. | Theory | Did the intervention report using a behavior change theory in its design? Select all that apply | *Select multiple*  (1) IBM-WASH  (2) RANAS  (3) Behavior Centered Design/Evo-Eco Model  (4) COM-B  (5) Theory of Planned Behavior  (6) Health Belief Model  (7) Social Ecological Model  (8) Theoretical Domains Framework  (777) Other – specify  (999) No theory reported | [White et al](https://www.sciencedirect.com/science/article/pii/S1438463919311101?via%3Dihub)  [The Handwashing Handbook](http://globalhandwashing.org/wp-content/uploads/2020/10/GHP_Handwashing-Handbook_FINAL.pdf) |
| 2 | Theory mention | Where did the authors discuss the theory that they used? | *Select one*  (1) Protocol  (2) Cited formative research  (3) Primary research paper  (777) Other – specify  (999) No theory reported |  |
| **RQ3.2d** **Among interventions to improve hand hygiene in community settings, what hand hygiene station designs have been effective at improving and sustaining hand hygiene?** | | | | |
| 1 | Handwashing station | Did the intervention use handwashing stations? | *Select one*  (0) No  (1) Yes  (999) Unclear |  |
| 1.1 | Handwashing station design types | If handwashing stations were used, what type was used? | *Select one*  (1) Tippy tap  (2) Raised bucket with tap/outlet  (3) Two buckets suspended  (4) Suspended bottle or bag with outlet/hole/pop-up plug  (5) Sink with tap  (6) Foot pump sink  (7) Purpose-built all-in-one system  (8) Free standing water tank with taps/outlets  (9) Tube with outlets  (777) Other - specify  (888) Not reported  (999) Not applicable | [UNICEF (2020) – Handwashing stations](https://www.unicef.org/media/75706/file/Handwashing%20Facility%20Worksheet.pdf) |
| 1.2 | Handwashing station design features – fixed vs mobile | If handwashing stations were used, was the station fixed or mobile? | *Select one*  (1) Fixed  (2) Mobile  (888) Not reported  (999) Not applicable | [UNICEF (2020) – Handwashing stations](https://www.unicef.org/media/75706/file/Handwashing%20Facility%20Worksheet.pdf) |
| 1.3 | Handwashing station design features – permanency | If handwashing stations were used, was the station permanent or temporary? | *Select one*  (1) Permanent  (2) Temporary  (888) Not reported  (999) Not applicable | [UNICEF (2020) – Handwashing stations](https://www.unicef.org/media/75706/file/Handwashing%20Facility%20Worksheet.pdf) |
| 1.4 | Handwashing station design features – users | If handwashing stations were used, who was the primary user base? | *Select one*  (1) Household  (2) Community  (3) Schools  (777) Other – specify  (888) Not reported  (999) Not applicable | [UNICEF (2020) – Handwashing stations](https://www.unicef.org/media/75706/file/Handwashing%20Facility%20Worksheet.pdf) |
| 1.5 | Handwashing station design features – water supply | If handwashing stations were used, what type of water supply was available? | *Select one*  (1) Individual storage tank  (2) Community storage tank  (3) Piped water  (777) Other – specify  (888) Not reported  (999) Not applicable | [UNICEF (2020) – Handwashing stations](https://www.unicef.org/media/75706/file/Handwashing%20Facility%20Worksheet.pdf) |
| 1.6 | Handwashing station design features – size | If handwashing stations were used, what was the size of the station? | Text  (888) Not reported  (999) Not applicable |  |
| 1.7 | Handwashing station design features – material | If handwashing station designs were used, what material were they made of? | Text  (888) Not reported  (999) Not applicable |  |
| 1.8 | Handwashing station design features – other | If handwashing stations were used, were there any other design features not yet captured? | Text  (888) Not reported  (999) Not applicable |  |
| **RQ3.2e** **Among interventions to improve hand hygiene in community settings, what design adaptations (e.g., placement, nudges, and cues) have been effective at improving and sustaining hand hygiene?** | | | | |
| 1 | Design adaptations | Did the intervention assess design adaptations of handwashing stations? | *Select one*  (0) No  (1) Yes  (999) Not applicable |  |
| 1.1 | Design adaptations comparison | How are the authors comparing their standard handwashing station design with their adapted design? | *Select one*  (1) Through multi-arm trial  (2) Comparing to design tested in earlier study  (999) Not applicable |  |
| 1.2 | Design adaptations standard | What was the handwashing station design that was the comparison/standard? | Text  (999) Not applicable |  |
| 1.3 | Design adaptations type | If design adaptations were implemented, what type of adaptation was used? | *Select one*  (1) Placement  (2) Nudges  (3) Cues  (777) Other – specify  (888) Not reported  (999) Not applicable |  |
| 1.4 | Design adaptation examples | If design adaptations were implemented, what were they? | *Text*  (888) Not reported  (999) Not applicable |  |
| 1.5 | Design adaptation effectiveness | Was the hand hygiene practice the same, better or worse in the adjusted design? | *Select one*  (1) Same  (2) Better  (3) Worse  (999) Not applicable |  |
| **RQ3.2f** **Among interventions to improve hand hygiene in community settings, what level of frequency and intensity of behavior change interventions is necessary to effectively improve hand hygiene?** | | | | |
| 1 | Frequency | Was the frequency of the behavior change intervention varied? | *Select one*  (0) No  (1) Yes  (999) Unclear |  |
| 1.1 | Frequency comparison | How are the authors comparing their standard level of frequency for the intervention with their adjusted design? | *Select one*  (1) Through multi-arm trial  (2) Comparing to design tested in earlier study  (999) Not applicable |  |
| 1.2 | Frequency standard | What level of frequency of the behavior change intervention was used as the comparison/standard? | Text  (999) Not applicable |  |
| 1.3 | Frequency level | What was the level of frequency of the behavior change intervention for the adjusted design? | Text  (999) Not applicable |  |
| 1.4 | Frequency level – additional arm | If the authors tested additional changes to their design (e.g., varying frequency in a 3^rd^ arm), please provide a description of the design change. | Text  (999) Not applicable |  |
| 1.5 | Frequency effectiveness | Was the hand hygiene practice the same, better or worse in the adjusted design? | *Select one*  (1) Same  (2) Better  (3) Worse  (999) Not applicable |  |
| 2 | Intensity | Was the intensity of the behavior change intervention varied? | *Select one*  (0) No  (1) Yes  (999) Unclear |  |
| 2.1 | Intensity comparison | How are the authors comparing their standard level of intensity for the intervention with their adjusted design? | *Select one*  (1) Through multi-arm trial  (2) Comparing to design tested in earlier study  (999) Not applicable |  |
| 2.2 | Intensity standard | What level of intensity of the behavior change intervention was used as the comparison/standard? | Text  (999) Not applicable |  |
| 2.3 | Intensity level | What was the level of intensity of the behavior change intervention for the adjusted design? | Text  (999) Not applicable |  |
| 2.4 | Intensity level – additional arm | If the authors tested additional changes to their design (e.g., varying intensity in a 3^rd^ arm), please provide a description of the design change. | Text  (999) Not applicable |  |
| 2.5 | Intensity effectiveness | Was the hand hygiene practice the same, better or worse in the adjusted design? | *Select one*  (1) Same  (2) Better  (3) Worse  (999) Not applicable |  |
| **RQ3.2g Among interventions to improve hand hygiene in community settings, how do hand hygiene practices vary by population groups, risk scenarios, or over time?** | | | | |
| 1 | Risk scenarios | Did the study evaluate hand hygiene in a risk scenario? | *Select one*  (0) No  (1) Yes  (999) Unclear |  |
| 1.1 | Risk scenario type | If yes, what was the risk scenario? | *Select one*  (1) COVID-19  (2) Flu  (3) Earthquake  (4) Flood  (5) Typhoon  (6) Forced migration  (7) Internal displacement  (8) Emergency setting  (777) Other – specify  (999) Not applicable |  |
| 1.2 | Risk scenario text | Please copy in the author’s text about the risk scenario. | Text (999) Not applicable |  |
| 2 | Over time | Did the study collect data at more than one time point after the intervention was implemented? | *Select one*  (0) No  (1) Yes  (999) Unclear |  |
| 2.1 | Over time – data points | If yes, how many time points did the study authors collect? | Text (999) Not applicable |  |
| 2.2 | Outcome category | What category does this outcome fall into? | *Select one*  (1) Frequency of handwashing  (2) Efficacy of handwashing  (3) Consistency of handwashing  (777) Other - specify |  |
| 2.3 | Key moment | What key moment was this outcome looking at? | *Select one*  (1) Before, during, and after preparing food  (2) Before and after eating food (3) Before and after caring for someone who is sick with vomiting or diarrhea  (4) Before and after treating a cut or wound  (5) Before feeding a child  (6) After using the toilet  (7) After changing diapers/cleaning a child’s bottom  (8) After blowing your nose, coughing or sneezing  (9) After touching an animal, animal feed, or animal waste  (10) After touching garbage  (11) After handling pet food or pet treats  (12) Key moments index – combination (13) No specified key moment  (777) Other - specify |  |
| 2.4 | Participants | Copy in the population that’s measured by the outcome | Text  (999) Not applicable |  |
| 2.5 | Study arm | If effect estimate is reported separately by study arm, indicate which arm is below. | Text  (999) Not applicable |  |
| 2.6 | Measurement | How was this outcome measured? | *Select one*  (1) Direct observation  (2) Self-reported  (3) Proxy indicator  (888) Not reported  (999) Not applicable |  |
| 2.6.1 | Direct observation indicator | Paste in the indicator/outcome description that authors used for measurement. | Text  (999) Not applicable |  |
| 2.6.2 | Self-report | If hand hygiene is assessed by self-reporting, paste the survey question used | Text  (999) Not applicable |  |
| 2.6.3 | Proxy indicator | If hand hygiene is assessed by proxy indicator, paste the indicator used | Text  (999) Not applicable |  |
| 2.6.4 | Sex disaggregation | Was the hand hygiene practice(s) above disaggregated by sex? | *Select one*  (0) No  (1) Yes  (999) Not applicable |  |
| 2.6.5 | Time elapsed | How much time has elapsed since the intervention | Text  (999) Not applicable |  |
| 2.7.1 | Intervention group observations | What is the number of observations in the intervention group for this outcome? | Text  (888) Not reported  (999) Not applicable |  |
| 2.7.2 | Additional intervention group observations | What is the number of observations in the additional intervention group for this outcome? | Text  (888) Not reported  (999) Not applicable |  |
| 2.7.3 | Control group observations | What is the number of observations in the control group for this outcome? | Text  (888) Not reported  (999) Not applicable |  |
| 2.8.1 | Effect estimate | What is the effect estimate of hand hygiene practice? | Text  (888) Not reported  (999) Not applicable |  |
| 2.8.2 | Effect measure type | What type of effect measure is provided above? | *Select one*  (1) Risk ratio  (2) Odds ratio  (3) Prevalence ratio  (4) Rate ratio  (5) Mean difference  (6) Risk difference  (7) Proportion  (8) Number of events  (9) Percentage  (777) Other – specify  (999) Not applicable |  |
| 2.8.3 | Adjusted | Was the effect measure adjusted? | *Select one*  (1) No  (2) Yes  (888) Not reported  (999) Not applicable |  |
| 2.8.4 | Units | Copy in the units used for the effect (i.e. percentage points, number of events, etc.) | Text  (888) Not reported  (999) Not applicable |  |
| 2.8.5 | Lower CI | What is the lower confidence interval bound? | Text  (888) Not reported (999) Not applicable |  |
| 2.8.6 | Upper CI | What is the upper confidence interval bound? | Text  (888) Not reported (999) Not applicable |  |
| 2.8.7 | p-value | What is the p-value? | Text  (888) Not reported (999) Not applicable |  |
| 2.8.8 | Standard deviation | What is the standard deviation? | Text  (888) Not reported (999) Not applicable |  |
|  |  |  |  |  |
|  | Notes | Any notes or comments to add about the study? | Text |  |

# Bias assessment

| **Bias Assessment** | | | | |
| --- | --- | --- | --- | --- |
| MMAT (All articles) | | | | |
| S1 | Screening question 1 (for all types) | Are there clear research questions? | (0) No  (1) Yes  (888) Can’t tell  (999) Not applicable | [MMAT User Guide](http://mixedmethodsappraisaltoolpublic.pbworks.com/w/file/fetch/127916259/MMAT_2018_criteria-manual_2018-08-01_ENG.pdf) |
| S2 | Screening question 2 (for all types) | Do the collected data allow to address the research questions? | (0) No  (1) Yes  (888) Can’t tell  (999) Not applicable | [MMAT User Guide](http://mixedmethodsappraisaltoolpublic.pbworks.com/w/file/fetch/127916259/MMAT_2018_criteria-manual_2018-08-01_ENG.pdf) |
| Qualitative | | | | |
| 1.1 | Is the qualitative approach appropriate to answer the research question? | | (0) No  (1) Yes  (888) Can’t tell  (999) Not applicable | [MMAT User Guide](http://mixedmethodsappraisaltoolpublic.pbworks.com/w/file/fetch/127916259/MMAT_2018_criteria-manual_2018-08-01_ENG.pdf) |
| 1.2 | Are the qualitative data collection methods adequate to address the research question? | | (0) No  (1) Yes  (888) Can’t tell  (999) Not applicable | [MMAT User Guide](http://mixedmethodsappraisaltoolpublic.pbworks.com/w/file/fetch/127916259/MMAT_2018_criteria-manual_2018-08-01_ENG.pdf) |
| 1.3 | Are the findings adequately derived from the data? | | (0) No  (1) Yes  (888) Can’t tell  (999) Not applicable | [MMAT User Guide](http://mixedmethodsappraisaltoolpublic.pbworks.com/w/file/fetch/127916259/MMAT_2018_criteria-manual_2018-08-01_ENG.pdf) |
| 1.4 | Is the interpretation of results sufficiently substantiated by data? | | (0) No  (1) Yes  (888) Can’t tell  (999) Not applicable | [MMAT User Guide](http://mixedmethodsappraisaltoolpublic.pbworks.com/w/file/fetch/127916259/MMAT_2018_criteria-manual_2018-08-01_ENG.pdf) |
| 1.5 | Is there coherence between qualitative data sources, collection, analysis and interpretation? | | (0) No  (1) Yes  (888) Can’t tell  (999) Not applicable | [MMAT User Guide](http://mixedmethodsappraisaltoolpublic.pbworks.com/w/file/fetch/127916259/MMAT_2018_criteria-manual_2018-08-01_ENG.pdf) |
| Quantitative randomized controlled trials | | | | |
| 2.1 | Is randomization appropriately performed? | | (0) No  (1) Yes  (888) Can’t tell  (999) Not applicable | [MMAT User Guide](http://mixedmethodsappraisaltoolpublic.pbworks.com/w/file/fetch/127916259/MMAT_2018_criteria-manual_2018-08-01_ENG.pdf) |
| 2.2 | Are the groups comparable at baseline? | | (0) No  (1) Yes  (888) Can’t tell  (999) Not applicable | [MMAT User Guide](http://mixedmethodsappraisaltoolpublic.pbworks.com/w/file/fetch/127916259/MMAT_2018_criteria-manual_2018-08-01_ENG.pdf) |
| 2.3 | Are there complete outcome data? | | (0) No  (1) Yes  (888) Can’t tell  (999) Not applicable | [MMAT User Guide](http://mixedmethodsappraisaltoolpublic.pbworks.com/w/file/fetch/127916259/MMAT_2018_criteria-manual_2018-08-01_ENG.pdf) |
| 2.4 | Are outcome assessors blinded to the intervention provided? | | (0) No  (1) Yes  (888) Can’t tell  (999) Not applicable | [MMAT User Guide](http://mixedmethodsappraisaltoolpublic.pbworks.com/w/file/fetch/127916259/MMAT_2018_criteria-manual_2018-08-01_ENG.pdf) |
| 2.5 | Did the participants adhere to the assigned intervention? | | (0) No  (1) Yes  (888) Can’t tell  (999) Not applicable | [MMAT User Guide](http://mixedmethodsappraisaltoolpublic.pbworks.com/w/file/fetch/127916259/MMAT_2018_criteria-manual_2018-08-01_ENG.pdf) |
| Quantitative non-randomized | | | | |
| 3.1 | Are the participants representative of the target population? | | (0) No  (1) Yes  (888) Can’t tell  (999) Not applicable | [MMAT User Guide](http://mixedmethodsappraisaltoolpublic.pbworks.com/w/file/fetch/127916259/MMAT_2018_criteria-manual_2018-08-01_ENG.pdf) |
| 3.2 | Are measurements appropriate regarding both the outcome and intervention (or exposure)? | | (0) No  (1) Yes  (888) Can’t tell  (999) Not applicable | [MMAT User Guide](http://mixedmethodsappraisaltoolpublic.pbworks.com/w/file/fetch/127916259/MMAT_2018_criteria-manual_2018-08-01_ENG.pdf) |
| 3.3 | Are there complete outcome data? | | (0) No  (1) Yes  (888) Can’t tell  (999) Not applicable | [MMAT User Guide](http://mixedmethodsappraisaltoolpublic.pbworks.com/w/file/fetch/127916259/MMAT_2018_criteria-manual_2018-08-01_ENG.pdf) |
| 3.4 | Are the confounders accounted for in the design and analysis? | | (0) No  (1) Yes  (888) Can’t tell  (999) Not applicable | [MMAT User Guide](http://mixedmethodsappraisaltoolpublic.pbworks.com/w/file/fetch/127916259/MMAT_2018_criteria-manual_2018-08-01_ENG.pdf) |
| 3.5 | During the study period, is the intervention administered (or exposure occurred) as intended? | | (0) No  (1) Yes  (888) Can’t tell  (999) Not applicable | [MMAT User Guide](http://mixedmethodsappraisaltoolpublic.pbworks.com/w/file/fetch/127916259/MMAT_2018_criteria-manual_2018-08-01_ENG.pdf) |
| Quantitative descriptive | | | | |
| 4.1 | Is the sampling strategy relevant to address the research question? | | (0) No  (1) Yes  (888) Can’t tell  (999) Not applicable | [MMAT User Guide](http://mixedmethodsappraisaltoolpublic.pbworks.com/w/file/fetch/127916259/MMAT_2018_criteria-manual_2018-08-01_ENG.pdf) |
| 4.2 | Is the sample representative of the target population? | | (0) No  (1) Yes  (888) Can’t tell  (999) Not applicable | [MMAT User Guide](http://mixedmethodsappraisaltoolpublic.pbworks.com/w/file/fetch/127916259/MMAT_2018_criteria-manual_2018-08-01_ENG.pdf) |
| 4.3 | Are the measurements appropriate? | | (0) No  (1) Yes  (888) Can’t tell  (999) Not applicable | [MMAT User Guide](http://mixedmethodsappraisaltoolpublic.pbworks.com/w/file/fetch/127916259/MMAT_2018_criteria-manual_2018-08-01_ENG.pdf) |
| 4.4 | Is the risk of nonresponse bias low? | | (0) No  (1) Yes  (888) Can’t tell  (999) Not applicable | [MMAT User Guide](http://mixedmethodsappraisaltoolpublic.pbworks.com/w/file/fetch/127916259/MMAT_2018_criteria-manual_2018-08-01_ENG.pdf) |
| 4.5 | Is the statistical analysis appropriate to answer the research question? | | (0) No  (1) Yes  (888) Can’t tell  (999) Not applicable | [MMAT User Guide](http://mixedmethodsappraisaltoolpublic.pbworks.com/w/file/fetch/127916259/MMAT_2018_criteria-manual_2018-08-01_ENG.pdf) |
| Mixed methods | | | | |
| 5.1 | Is there an adequate rationale for using a mixed methods design to address the research question? | | (0) No  (1) Yes  (888) Can’t tell  (999) Not applicable | [MMAT User Guide](http://mixedmethodsappraisaltoolpublic.pbworks.com/w/file/fetch/127916259/MMAT_2018_criteria-manual_2018-08-01_ENG.pdf) |
| 5.2 | Are the different components of the study effectively integrated to answer the research question? | | (0) No  (1) Yes  (888) Can’t tell  (999) Not applicable | [MMAT User Guide](http://mixedmethodsappraisaltoolpublic.pbworks.com/w/file/fetch/127916259/MMAT_2018_criteria-manual_2018-08-01_ENG.pdf) |
| 5.3 | Are the outputs of the integration of qualitative and quantitative components adequately interpreted? | | (0) No  (1) Yes  (888) Can’t tell  (999) Not applicable | [MMAT User Guide](http://mixedmethodsappraisaltoolpublic.pbworks.com/w/file/fetch/127916259/MMAT_2018_criteria-manual_2018-08-01_ENG.pdf) |
| 5.4 | Are divergences and inconsistencies between quantitative and qualitative results adequately addressed? | | (0) No  (1) Yes  (888) Can’t tell  (999) Not applicable | [MMAT User Guide](http://mixedmethodsappraisaltoolpublic.pbworks.com/w/file/fetch/127916259/MMAT_2018_criteria-manual_2018-08-01_ENG.pdf) |
| 5.5 | Do the different components of the study adhere to the quality criteria of each tradition of the methods involved? | | (0) No  (1) Yes  (888) Can’t tell  (999) Not applicable | [MMAT User Guide](http://mixedmethodsappraisaltoolpublic.pbworks.com/w/file/fetch/127916259/MMAT_2018_criteria-manual_2018-08-01_ENG.pdf) |
