# Supplementary material for: Interventions to improve hand hygiene in community settings: a systematic review of theories, barriers and enablers, behaviour change techniques and hand hygiene station design features
Source: BMJ Glob Health. 2025 Sep 16;10(Suppl 7):e018928. doi: 10.1136/bmjgh-2025-018928 (PMC12443188; doi:10.1136/bmjgh-2025-018928)
Supplement: online supplemental file 9 [file bmjgh-10-Suppl_7-s009.docx]

**Interventions to improve hand hygiene in community settings: A systematic review of theories, barriers and enablers, behavior change techniques, and hand hygiene station design features**

*Authors*

Sridevi K. Prasad^1^ 0000-0003-0457-9534

Jedidiah S. Snyder^2^ 0000-0002-7688-4450

Erin LaFon^2^

Lilly A. O’Brien^2^ 0009-0004-1987-3706

Hannah Rogers^3^ 0000-0002-9515-1439

Oliver Cumming^4,5^ 0000-0002-5074-8709

Joanna Esteves Mills^5^

Bruce Gordon ^5^

Marlene Wolfe^2^ 0000-0002-6476-0450

Matthew C. Freeman^2^ 0000-0002-1517-2572

Bethany A. Caruso^1*^ 0000-0001-9738-9857

1 Hubert Department of Global Health, Rollins School of Public Health, Emory University, Atlanta, GA, USA; [bcaruso@emory.edu](mailto:bcaruso@emory.edu) (BAC); [sridevi.prasad@emory.edu](mailto:sridevi.prasad@emory.edu) (SKP)

2 Gangarosa Department of Environmental Health, Rollins School of Public Health, Emory University, Atlanta, GA, USA; [matthew.freeman@emory.edu](mailto:matthew.freeman@emory.edu) (MCF); [marlene.wolfe@emory.edu](mailto:marlene.wolfe@emory.edu) (MW) [jedidiah.snyder@emory.edu](mailto:jedidiah.snyder@emory.edu) (JSS); [lilly.obrien@emory.edu](mailto:lilly.obrien@emory.edu) (LAO); [erin.lafon@emory.edu](mailto:erin.lafon@emory.edu) (EL)

3 Woodruff Health Sciences Center Library, Emory University, Atlanta, GA, USA; [hannah.rogers@emory.edu](mailto:hannah.rogers@emory.edu) (HR)

4 Department of Disease Control, London School of Hygiene and Tropical Medicine, London, UK; [oliver.cumming@lshtm.ac.uk](mailto:oliver.cumming@lshtm.ac.uk) (OC)

5 Water, Sanitation, Hygiene and Health Unit, World Health Organization, Geneva, Switzerland; [estevesj@who.int](mailto:estevesj@who.int) (JEM); [gordonb@who.int](mailto:gordonb@who.int) (BG)

*Corresponding author: Bethany A. Caruso [bcaruso@emory.edu](mailto:bcaruso@emory.edu)

Emory University, Rollins School of Public Health, 1518 Clifton Rd, Atlanta, GA 30322

***Supplementary File 9*.** Theories or models reported to be used in included studies (N=223).

| **Theory** | **Total**  **n (%)** | **Reported Effective****  **n (%)** |
| --- | --- | --- |
| **Total Studies** | **223** | **183 (82.1)** |
| **Study did not report using theory for intervention design** | **160 (71.8)** | **131 (81.9)** |
| **Study did report using theory for intervention design*** | **63 (28.2)** | **52 (82.5)** |
| Theory of Planned Behavior | 14 (22.2) | 10 (71.4) |
| Health Belief Model | 11 (17.5) | 10 (90.9) |
| Behaviour Centered Design/Evo-Eco Model | 7 (11.1) | 5 (71.4) |
| RANAS | 6 (9.5) | 5 (83.3) |
| COM-B | 6 (9.5) | 5 (83.3) |
| IBM-WASH | 5 (7.9) | 4 (80.0) |
| Health Access Process Approach | 2 (3.2) | 1 (50.0) |
| Social Ecological Model | 2 (3.2) | 2 (100.0) |
| Social-cognitive Learning Theory | 2 (3.2) | 2 (100.0) |
| Stages of Change Model | 2 (3.2) | 2 (100.0) |
| Theory of Normative Social Behavior | 2 (3.2) | 2 (100.0) |
| Theory of Reasoned Action | 2 (3.2) | 2 (100.0) |
| Behavior Place Theory | 1 (1.6) | 1 (100.0) |
| Bloom's Taxonomy of Learning Theory | 1 (1.6) | 1 (100.0) |
| Choice-architecture Approach | 1 (1.6) | 1 (100.0) |
| Ideation Theory | 1 (1.6) | 1 (100.0) |
| Dual Process Theory | 1 (1.6) | 0 (0.0) |
| Ecological Theory of Health Promotion | 1 (1.6) | 1 (100.0) |
| Health Behavior Change Model | 1 (1.6) | 1 (100.0) |
| Heuristic Model for Teachable Moments | 1 (1.6) | 1 (100.0) |
| Organization Behavior Modification Model | 1 (1.6) | 1 (100.0) |
| Focus Theory | 1 (1.6) | 1 (100.0) |
| Nudge Theory | 1 (1.6) | 1 (100.0) |
| Goal Attainment Model | 1 (1.6) | 1 (100.0) |
| Social Cognitive Theory | 1 (1.6) | 1 (100.0) |

***Note: *****Multiple theories could have been reported per study so the total number of theories (74) is greater than the total number of studies that report using theory (63).; **Reported effectiveness is determined if authors reported that the intervention was effective at improving hand hygiene outcomes*
