# Supplementary material for: Interventions to improve hand hygiene in community settings: a systematic review of theories, barriers and enablers, behaviour change techniques and hand hygiene station design features
Source: BMJ Glob Health. 2025 Sep 16;10(Suppl 7):e018928. doi: 10.1136/bmjgh-2025-018928 (PMC12443188; doi:10.1136/bmjgh-2025-018928)
Supplement: online supplemental file 11 [file bmjgh-10-Suppl_7-s011.docx]

**Interventions to improve hand hygiene in community settings: A systematic review of theories, barriers and enablers, behavior change techniques, and hand hygiene station design features**

*Authors*

Sridevi K. Prasad^1^ 0000-0003-0457-9534

Jedidiah S. Snyder^2^ 0000-0002-7688-4450

Erin LaFon^2^

Lilly A. O’Brien^2^ 0009-0004-1987-3706

Hannah Rogers^3^ 0000-0002-9515-1439

Oliver Cumming^4,5^ 0000-0002-5074-8709

Joanna Esteves Mills^5^

Bruce Gordon ^5^

Marlene Wolfe^2^ 0000-0002-6476-0450

Matthew C. Freeman^2^ 0000-0002-1517-2572

Bethany A. Caruso^1*^ 0000-0001-9738-9857

1 Hubert Department of Global Health, Rollins School of Public Health, Emory University, Atlanta, GA, USA; [bcaruso@emory.edu](mailto:bcaruso@emory.edu) (BAC); [sridevi.prasad@emory.edu](mailto:sridevi.prasad@emory.edu) (SKP)

2 Gangarosa Department of Environmental Health, Rollins School of Public Health, Emory University, Atlanta, GA, USA; [matthew.freeman@emory.edu](mailto:matthew.freeman@emory.edu) (MCF); [marlene.wolfe@emory.edu](mailto:marlene.wolfe@emory.edu) (MW) [jedidiah.snyder@emory.edu](mailto:jedidiah.snyder@emory.edu) (JSS); [lilly.obrien@emory.edu](mailto:lilly.obrien@emory.edu) (LAO); [erin.lafon@emory.edu](mailto:erin.lafon@emory.edu) (EL)

3 Woodruff Health Sciences Center Library, Emory University, Atlanta, GA, USA; [hannah.rogers@emory.edu](mailto:hannah.rogers@emory.edu) (HR)

4 Department of Disease Control, London School of Hygiene and Tropical Medicine, London, UK; [oliver.cumming@lshtm.ac.uk](mailto:oliver.cumming@lshtm.ac.uk) (OC)

5 Water, Sanitation, Hygiene and Health Unit, World Health Organization, Geneva, Switzerland; [estevesj@who.int](mailto:estevesj@who.int) (JEM); [gordonb@who.int](mailto:gordonb@who.int) (BG)

*Corresponding author: Bethany A. Caruso [bcaruso@emory.edu](mailto:bcaruso@emory.edu)

Emory University, Rollins School of Public Health, 1518 Clifton Rd, Atlanta, GA 30322

***Supplementary File 11*. Representative examples of reported and addressed classifications**

| **COM-B Component** | | **Reported but not addressed** | **Addressed but not reported** |
| --- | --- | --- | --- |
| **Capability** | |  |  |
|  | *Physical* | N/A | N/A |
|  | *Psychological* | N/A | Briceño (2017) used multimedia messages and community health worker visits to improve knowledge of hand hygiene behavior but did not report **action knowledge** as a barrier |
| **Opportunity** | |  |  |
|  | *Physical* | Ashraf (2017) reported **water availability** as an enabler but the intervention provided soap and a handwashing station without water provision | Biran (2014) provided a handwashing station but did not report any barriers or enablers related to **handwashing infrastructure** |
|  | *Social* | Capps (2022) reported **social pressure** as a barrier and enabler but the intervention involved placing posters near hand sanitizer dispensers | Edward (2019) used visits from community health workers to improve hand hygiene behavior but did not report **network support** or **role modeling** as enablers |
| **Motivation** | |  |  |
|  | *Automatic* | Huang (2021) reported **internal motivation** as a barrier but provided a handwashing station | Tousman (2011) used diaries and journals to stimulate hand hygiene as a habit but did not report **internal motivation** as a barrier |
|  | *Reflective* | Abbot (2012) reported **time prioritization** as a barrier but used multimedia videos to instruct on how to perform hand hygiene behavior | Greene (2012) implemented a school-based education program that discussed the health consequences from inadequate hand hygiene but did not report **disease risk** as an enabler |
