# Supplementary material for: Interventions to improve hand hygiene in community settings: a systematic review of theories, barriers and enablers, behaviour change techniques and hand hygiene station design features
Source: BMJ Glob Health. 2025 Sep 16;10(Suppl 7):e018928. doi: 10.1136/bmjgh-2025-018928 (PMC12443188; doi:10.1136/bmjgh-2025-018928)
Supplement: online supplemental file 12 [file bmjgh-10-Suppl_7-s012.docx]

**Interventions to improve hand hygiene in community settings: A systematic review of theories, barriers and enablers, behavior change techniques, and hand hygiene station design features**

*Authors*

Sridevi K. Prasad^1^ 0000-0003-0457-9534

Jedidiah S. Snyder^2^ 0000-0002-7688-4450

Erin LaFon^2^

Lilly A. O’Brien^2^ 0009-0004-1987-3706

Hannah Rogers^3^ 0000-0002-9515-1439

Oliver Cumming^4,5^ 0000-0002-5074-8709

Joanna Esteves Mills^5^

Bruce Gordon ^5^

Marlene Wolfe^2^ 0000-0002-6476-0450

Matthew C. Freeman^2^ 0000-0002-1517-2572

Bethany A. Caruso^1*^ 0000-0001-9738-9857

1 Hubert Department of Global Health, Rollins School of Public Health, Emory University, Atlanta, GA, USA; [bcaruso@emory.edu](mailto:bcaruso@emory.edu) (BAC); [sridevi.prasad@emory.edu](mailto:sridevi.prasad@emory.edu) (SKP)

2 Gangarosa Department of Environmental Health, Rollins School of Public Health, Emory University, Atlanta, GA, USA; [matthew.freeman@emory.edu](mailto:matthew.freeman@emory.edu) (MCF); [marlene.wolfe@emory.edu](mailto:marlene.wolfe@emory.edu) (MW) [jedidiah.snyder@emory.edu](mailto:jedidiah.snyder@emory.edu) (JSS); [lilly.obrien@emory.edu](mailto:lilly.obrien@emory.edu) (LAO); [erin.lafon@emory.edu](mailto:erin.lafon@emory.edu) (EL)

3 Woodruff Health Sciences Center Library, Emory University, Atlanta, GA, USA; [hannah.rogers@emory.edu](mailto:hannah.rogers@emory.edu) (HR)

4 Department of Disease Control, London School of Hygiene and Tropical Medicine, London, UK; [oliver.cumming@lshtm.ac.uk](mailto:oliver.cumming@lshtm.ac.uk) (OC)

5 Water, Sanitation, Hygiene and Health Unit, World Health Organization, Geneva, Switzerland; [estevesj@who.int](mailto:estevesj@who.int) (JEM); [gordonb@who.int](mailto:gordonb@who.int) (BG)

*Corresponding author: Bethany A. Caruso [bcaruso@emory.edu](mailto:bcaruso@emory.edu)

Emory University, Rollins School of Public Health, 1518 Clifton Rd, Atlanta, GA 30322

***Supplementary Table 12*.** Frequency of identified behavior change technique (BCT) packages and frequency of BCTs per package with reported effectiveness across included studies (N=223)

| **Number of BCT packages per study** | **Number and % of studies** | **Reported Effectiveness^2^** **n (%)** | | **Number of BCTs per package** | **Number and % of studies^1^** | **Reported Effectiveness^2^** **n (%)** |
| --- | --- | --- | --- | --- | --- | --- |
| 1 package | 100 (44.8%) | 78 (78.0%) | | 1 BCT | 71 (31.8%) | 57 (80.3%) |
| 2 packages | 62 (27.8%) | 53 (85.5%) | | 2 BCTs | 167 (74.9%) | 143 (85.6%) |
| 3 packages | 38 (17.0%) | 33 (86.8%) | | 3 BCTs | 109 (48.8%) | 87 (79.8%) |
| 4 packages | 17 (7.6%) | 13 (76.5%) | |  |  |  |
| 5 packages | 5 (2.2%) | 5 (100.0%) | |  |  |  |
| 6 packages | 1 (0.5%) | 1 (100.0%) | |  |  |  |
| Total Packages^1^ | 437 |  |  |  |  |  |

***Note:*** ^1^Adds up to more than the total number of studies included as studies could have evaluated packages with different numbers of BCTs*; ^2^Reported effectiveness is determined if authors reported that the intervention was effective at improving hand hygiene outcomes*
