# Supplementary material for: Interventions to improve hand hygiene in community settings: a systematic review of theories, barriers and enablers, behaviour change techniques and hand hygiene station design features
Source: BMJ Glob Health. 2025 Sep 16;10(Suppl 7):e018928. doi: 10.1136/bmjgh-2025-018928 (PMC12443188; doi:10.1136/bmjgh-2025-018928)
Supplement: online supplemental file 14 [file bmjgh-10-Suppl_7-s014.docx]

**Interventions to improve hand hygiene in community settings: A systematic review of theories, barriers and enablers, behavior change techniques, and hand hygiene station design features**

*Authors*

Sridevi K. Prasad^1^ 0000-0003-0457-9534

Jedidiah S. Snyder^2^ 0000-0002-7688-4450

Erin LaFon^2^

Lilly A. O’Brien^2^ 0009-0004-1987-3706

Hannah Rogers^3^ 0000-0002-9515-1439

Oliver Cumming^4,5^ 0000-0002-5074-8709

Joanna Esteves Mills^5^

Bruce Gordon ^5^

Marlene Wolfe^2^ 0000-0002-6476-0450

Matthew C. Freeman^2^ 0000-0002-1517-2572

Bethany A. Caruso^1*^ 0000-0001-9738-9857

1 Hubert Department of Global Health, Rollins School of Public Health, Emory University, Atlanta, GA, USA; [bcaruso@emory.edu](mailto:bcaruso@emory.edu) (BAC); [sridevi.prasad@emory.edu](mailto:sridevi.prasad@emory.edu) (SKP)

2 Gangarosa Department of Environmental Health, Rollins School of Public Health, Emory University, Atlanta, GA, USA; [matthew.freeman@emory.edu](mailto:matthew.freeman@emory.edu) (MCF); [marlene.wolfe@emory.edu](mailto:marlene.wolfe@emory.edu) (MW) [jedidiah.snyder@emory.edu](mailto:jedidiah.snyder@emory.edu) (JSS); [lilly.obrien@emory.edu](mailto:lilly.obrien@emory.edu) (LAO); [erin.lafon@emory.edu](mailto:erin.lafon@emory.edu) (EL)

3 Woodruff Health Sciences Center Library, Emory University, Atlanta, GA, USA; [hannah.rogers@emory.edu](mailto:hannah.rogers@emory.edu) (HR)

4 Department of Disease Control, London School of Hygiene and Tropical Medicine, London, UK; [oliver.cumming@lshtm.ac.uk](mailto:oliver.cumming@lshtm.ac.uk) (OC)

5 Water, Sanitation, Hygiene and Health Unit, World Health Organization, Geneva, Switzerland; [estevesj@who.int](mailto:estevesj@who.int) (JEM); [gordonb@who.int](mailto:gordonb@who.int) (BG)

*Corresponding author: Bethany A. Caruso [bcaruso@emory.edu](mailto:bcaruso@emory.edu)

Emory University, Rollins School of Public Health, 1518 Clifton Rd, Atlanta, GA 30322

***Supplementary Text and Table 14:***

Discussion

Consistent with other reviews, implementing hand hygiene stations were found to be effective in improving hand hygiene practice (Mbakaya et al. 2020). Among the 223 studies reviewed, 21% (n=46) reported using a hand hygiene station in their intervention design (**S14 Table below**). Data from a 2020 review on the use of tippy-taps on hand hygiene revealed there were increases in hand hygiene practice after the tippy-tap had been introduced among the studies that measured hand hygiene practice (Mbakaya et al. 2020). In this review, 80% of studies that used hand hygiene stations were effective in improving hand hygiene outcomes. The most common design being a raised bucket with tap/outlet, showing a 75% effectiveness rate. The effectiveness varied based on station features such as mobility, permanency, and water supply, with fixed, permanent stations with piped water supply showing higher effectiveness rates. However, the analysis of design features and hand hygiene station types was limited by incomplete reporting on station characteristics and settings across many studies, further underscoring the need for more comprehensive data and reporting.

**S14 Table. Hand hygiene station types and designs features reported in included studies (N=223)**

| **Hand hygiene station types and designs features** | **Total**  **n (%)** | **Reported Effective****  **n (%)** |
| --- | --- | --- |
| **Studies reporting any handwashing station types and design features** | **46 (20.6)** | **37 (80.4)** |
| **Users** |  |  |
| School Populations | 24 (52.2) | 20 (83.3) |
| Household Populations | 15 (32.6) | 12 (80.0) |
| Community Members | 3 (6.5) | 2 (66.7) |
| Migrant Workers | 1 (2.2) | 1 (100.0) |
| Workplace Populations | 1 (2.2) | 1 (100.0) |
| Not reported | 2 (4.4) | 1 (50.0) |
| **Total Studies Reporting on Hand Washing Stations** | **46 (100.0)** | **37 (80.4)** |
| Raised bucket with tap/outlet | 20 (44.4) | 15 (75.0) |
| Sink with tap | 7 (15.6) | 7 (100.0) |
| Tippy tap | 4 (8.9) | 4 (100.0) |
| Free standing water tank with taps | 1 (2.2) | 1 (100.0) |
| Foot pump sink | 1 (2.2) | 0 ( 0.0) |
| Hand pump | 1 (2.2) | 1 (100.0) |
| Purpose-built all-in-one system | 1 (2.2) | 1 (100.0) |
| Two buckets suspended | 1 (2.2) | 1 (100.0) |
| Handwashing Station Type Not reported | 10 (22.2) | 7 (70.0) |
| **Mobility** |  |  |
| Mobile | 13 (28.3) | 9 (69.2) |
| Fixed | 12 (26.1) | 11 (91.7) |
| Not reported | 21 (45.7) | 17 (81.0) |
| **Permanency** |  |  |
| Temporary | 15 (32.6) | 11 (73.3) |
| Permanent | 11 (23.9) | 10 (90.9) |
| Not reported | 20 (43.5) | 16 (80.0) |
| **Water supply** |  |  |
| Individual storage tank | 13 (28.3) | 11 (84.6) |
| Piped water | 5 (10.9) | 5 (100.0) |
| Not reported | 28 (60.9) | 21 (75.0) |
| **Material** |  |  |
| Plastic | 7 (15.2) | 7 (100.0) |
| Not reported | 39 (84.8) | 30 (76.9) |
| **Location*** |  |  |
| Near baby's room | 2 (4.1) | 1 (50.0) |
| Near cooking area | 2 (4.1) | 0 (0.0) |
| Near construction sites | 1 (2.0) | 1 (100.0) |
| Near markets | 1 (2.0) | 1 (100.0) |
| Near bathroom | 1 (2.0) | 0 (0.0) |
| Not reported | 42 (85.7) | 35 (83.3) |
| **Additional features** |  |  |
| Additional soap provided | 5 (10.9) | 4 (80.0) |
| Added cues to action (stickers, painted footpaths) | 3 (6.5) | 1 (33.3) |
| Bowl for run-off water provided | 3 (6.5) | 3 (100.0) |
| Additional water storage provided | 2 (4.4) | 1 (50.0) |
| Mirror | 2 (4.4) | 2 (100.0) |
| Aesthetic features | 1 (2.2) | 1 (100.0) |
| Addition features not reported | 30 (65.2) | 25 (83.3) |
| **Studies reporting handwashing station storage capacity (n=13)** | **Total**  **mean (SD)** | **Effective**  **mean (SD)** |
| Average storage capacity in L | 43.5 (29.8) | 46 (31.7) |

*Note: *Location is multi-select so could have multiple locations per handwashing station study; ** Reported effectiveness is determined if authors reported that the intervention was effective at improving hand hygiene outcomes*
