# Supplementary material for: Interventions to improve hand hygiene in community settings: a systematic review of theories, barriers and enablers, behaviour change techniques and hand hygiene station design features
Source: BMJ Glob Health. 2025 Sep 16;10(Suppl 7):e018928. doi: 10.1136/bmjgh-2025-018928 (PMC12443188; doi:10.1136/bmjgh-2025-018928)
Supplement: online supplemental file 15 [file bmjgh-10-Suppl_7-s015.docx]

**Interventions to improve hand hygiene in community settings: A systematic review of theories, barriers and enablers, behavior change techniques, and hand hygiene station design features**

*Authors*

Sridevi K. Prasad^1^ 0000-0003-0457-9534

Jedidiah S. Snyder^2^ 0000-0002-7688-4450

Erin LaFon^2^

Lilly A. O’Brien^2^ 0009-0004-1987-3706

Hannah Rogers^3^ 0000-0002-9515-1439

Oliver Cumming^4,5^ 0000-0002-5074-8709

Joanna Esteves Mills^5^

Bruce Gordon ^5^

Marlene Wolfe^2^ 0000-0002-6476-0450

Matthew C. Freeman^2^ 0000-0002-1517-2572

Bethany A. Caruso^1*^ 0000-0001-9738-9857

1 Hubert Department of Global Health, Rollins School of Public Health, Emory University, Atlanta, GA, USA; [bcaruso@emory.edu](mailto:bcaruso@emory.edu) (BAC); [sridevi.prasad@emory.edu](mailto:sridevi.prasad@emory.edu) (SKP)

2 Gangarosa Department of Environmental Health, Rollins School of Public Health, Emory University, Atlanta, GA, USA; [matthew.freeman@emory.edu](mailto:matthew.freeman@emory.edu) (MCF); [marlene.wolfe@emory.edu](mailto:marlene.wolfe@emory.edu) (MW) [jedidiah.snyder@emory.edu](mailto:jedidiah.snyder@emory.edu) (JSS); [lilly.obrien@emory.edu](mailto:lilly.obrien@emory.edu) (LAO); [erin.lafon@emory.edu](mailto:erin.lafon@emory.edu) (EL)

3 Woodruff Health Sciences Center Library, Emory University, Atlanta, GA, USA; [hannah.rogers@emory.edu](mailto:hannah.rogers@emory.edu) (HR)

4 Department of Disease Control, London School of Hygiene and Tropical Medicine, London, UK; [oliver.cumming@lshtm.ac.uk](mailto:oliver.cumming@lshtm.ac.uk) (OC)

5 Water, Sanitation, Hygiene and Health Unit, World Health Organization, Geneva, Switzerland; [estevesj@who.int](mailto:estevesj@who.int) (JEM); [gordonb@who.int](mailto:gordonb@who.int) (BG)

*Corresponding author: Bethany A. Caruso [bcaruso@emory.edu](mailto:bcaruso@emory.edu)

Emory University, Rollins School of Public Health, 1518 Clifton Rd, Atlanta, GA 30322

***Supplementary Text and Table 15:***

Results

Ten studies evaluated hand hygiene station design adaptations, six (40%) of which performed better than the standard design (**S15 Table below**). Five studies evaluated the use of ‘nudges’ (e.g., painted or paved footpaths), and only three (60%) performed better than the standard design; the other two performed the same. Successful ‘nudge’ adaptations used painted footpaths (Huang et al. 2021; Prasetyo et al. 2022) or arrows (Weijers et al. 2021). Adaptations that performed the same as the standard design employed similar nudges; both had paved footpaths and painted handwashing stations, and one also had painted shoeprints. Three evaluated cues to action (e.g., posters, automatic towel availability); two performed better and one performed worse. Successful ‘cue to action’ adaptations included providing a poster indicating higher prevalence (i.e., from one out of five to four out of five) of hand hygiene practices among the target user (Lapinski et al. 2013) or having towels that are automatically available without user action compared to motion-activated dispensers (Ford et al. 2014). The ‘cue to action adaptation that performed worse was a poster warning about flu transmission, compared to the standard design which offered handwashing instructions (Davis et al. 2013). Two studies evaluated changes to placement; placement in front of a nature-style background performed better than the standard in front of a simple background (Bai et al. 2022), and placement by a mirror performed worse (Thorseth et al. 2021).

Discussion

Among the limited sample, hand hygiene adaptations were effective, though more research is warranted. Of the 46 studies that implemented a hand hygiene station, only ten studies examined adaptations in hand hygiene station design. Of the six studies that outperformed the original design, successful adaptations included aesthetic improvements (Bai et al. 2022), nudges such as painted footpaths and arrows (Huang et al. 2021; Prasetyo et al. 2022; Weijers et al. 2021), and cues to action, such as posters indicating higher hand hygiene prevalence or readily available towels (Lapinski et al. 2013; Ford et al. 2014). Other research has highlighted the importance of design adaptations in improving acceptability and adoption of the hand hygiene station. In a qualitative assessment of hand hygiene stations in displacement camps, participants reported that there would be greater acceptability of the hand hygiene station if the station had been redesigned to be more aesthetically pleasing and functional (White et al. 2022). Since only ten studies evaluated adaptations in hand hygiene station design within this review, additional research on design adaptations is needed to understand how adaptations may improve effectiveness of hand hygiene station interventions.

**S15 Table. Design adaptations of hand hygiene stations.**

| **Study ID, Country, Setting** | **Outcome of interest** | **Standard design** | **Design adaptation** | **Reported Effectiveness*** |
| --- | --- | --- | --- | --- |
| Davis 2013**  United States; Universities | Daily soap usage | Handwashing instructions on posters | **Cues to action** - Poster warnings of flu transmission | Adaptation performed worse |
| Lapinski 2013**  United States; Universities | Observed handwashing | Low-prevalence poster ("One out of five college students wash their hands EVERY time they use the bathroom") | **Cues to action** - High-prevalence poster ("Four out of five college students wash their hands EVERY time they use the bathroom") | Adaptation performed better |
| Ford 2014**  United States; Universities | Weekly soap consumption | Motion-activated towel dispenser | **Cues to action** - Towel was automatically available without action from users | Adaptation performed better |
| Dreibelbis 2016  Bangladesh; Schools | Observed handwashing after toileting events | No nudges | **“Nudges”***** - Paved footpath with painted handwashing station | Adaptation performed the same |
| Grover 2018 Bangladesh; Schools | Observed handwashing after toileting events | No nudges | **“Nudges”***** - Paved footpath, painted handwashing station, and painted shoeprints | Adaptation performed the same |
| Huang 2021  Philippines; Schools | Observed handwashing with soap after toilet use | No painted footpath or other nudges added | **“Nudges”***** - Painted footpath with an arrow sticker | Adaptation performed better |
| Prasetyo 2022  Indonesia; Workplaces | Observed handwashing with water or soap | Sink alone | **“Nudges”*** -** Bright yellow footprints painted | Adaptation performed better |
| Weijers 2020**  The Netherlands; Markets | Observed hand disinfection upon entering store | Message on dispenser | **“Nudges”***** - Message combined with three blue arrows pointing to dispenser | Adaptation performed better |
| Bai 2022  China; Schools | Observed handwashing rates | Simple background with manual faucet | **Placement** – Aesthetic background (nature/wood) with spotlight and automatic faucet | Adaptation performed better |
| Thorseth 2021  Ethiopia; Internally displaced people camps | Observed handwashing with soap at critical events (after defecation, before preparing food, before eating, before serving/feeding another person food, and after cleaning a child's bottom) | No additions | **Placement** - Addition of a mirror | Adaptation performed worse |

***Notes:*** *Grey highlights are adaptations that performed better; *Reported effectiveness is determined if authors reported that the intervention was effective at improving hand hygiene outcomes; **Interventions that did not provide a handwashing station but used cues and “nudges” to modify/adapt an existing handwashing station; ***Interventions are classified as “nudges” as study authors used the term to characterize their own work;*
