# Supplementary material for: Interventions to improve hand hygiene in community settings: a systematic review of theories, barriers and enablers, behaviour change techniques and hand hygiene station design features
Source: BMJ Glob Health. 2025 Sep 16;10(Suppl 7):e018928. doi: 10.1136/bmjgh-2025-018928 (PMC12443188; doi:10.1136/bmjgh-2025-018928)
Supplement: online supplemental file 16 [file bmjgh-10-Suppl_7-s016.docx]

**Interventions to improve hand hygiene in community settings: A systematic review of theories, barriers and enablers, behavior change techniques, and hand hygiene station design features**

*Authors*

Sridevi K. Prasad^1^ 0000-0003-0457-9534

Jedidiah S. Snyder^2^ 0000-0002-7688-4450

Erin LaFon^2^

Lilly A. O’Brien^2^ 0009-0004-1987-3706

Hannah Rogers^3^ 0000-0002-9515-1439

Oliver Cumming^4,5^ 0000-0002-5074-8709

Joanna Esteves Mills^5^

Bruce Gordon ^5^

Marlene Wolfe^2^ 0000-0002-6476-0450

Matthew C. Freeman^2^ 0000-0002-1517-2572

Bethany A. Caruso^1*^ 0000-0001-9738-9857

1 Hubert Department of Global Health, Rollins School of Public Health, Emory University, Atlanta, GA, USA; [bcaruso@emory.edu](mailto:bcaruso@emory.edu) (BAC); [sridevi.prasad@emory.edu](mailto:sridevi.prasad@emory.edu) (SKP)

2 Gangarosa Department of Environmental Health, Rollins School of Public Health, Emory University, Atlanta, GA, USA; [matthew.freeman@emory.edu](mailto:matthew.freeman@emory.edu) (MCF); [marlene.wolfe@emory.edu](mailto:marlene.wolfe@emory.edu) (MW) [jedidiah.snyder@emory.edu](mailto:jedidiah.snyder@emory.edu) (JSS); [lilly.obrien@emory.edu](mailto:lilly.obrien@emory.edu) (LAO); [erin.lafon@emory.edu](mailto:erin.lafon@emory.edu) (EL)

3 Woodruff Health Sciences Center Library, Emory University, Atlanta, GA, USA; [hannah.rogers@emory.edu](mailto:hannah.rogers@emory.edu) (HR)

4 Department of Disease Control, London School of Hygiene and Tropical Medicine, London, UK; [oliver.cumming@lshtm.ac.uk](mailto:oliver.cumming@lshtm.ac.uk) (OC)

5 Water, Sanitation, Hygiene and Health Unit, World Health Organization, Geneva, Switzerland; [estevesj@who.int](mailto:estevesj@who.int) (JEM); [gordonb@who.int](mailto:gordonb@who.int) (BG)

*Corresponding author: Bethany A. Caruso [bcaruso@emory.edu](mailto:bcaruso@emory.edu)

Emory University, Rollins School of Public Health, 1518 Clifton Rd, Atlanta, GA 30322

***Supplementary Text and Table***

Six studies evaluated adaptations in the frequency (n=1) or intensity (n=5) of behavior change interventions, three of which—all assessing higher intensity—performed better than the standard design (**S16 Table**). Successful adaptations included providing hygiene promotion activities (videos, Glo Germ, demonstrations, posters) with handwashing stations compared to providing handwashing stations alone (Amon-Tanoh et al. 2021); providing an enhanced community-led total sanitation (CLTS) approach with additional discussions, skit, films, pledges, stickers, and report cards compared to CLTS alone (Biran et al. 2022); and providing household visits from social workers and community health volunteers with trainings compared to trainings alone (Freeman et al. 2020).

**S16 Table. Variations in frequency and intensity of hand hygiene interventions**

| **Study ID**  **Country, Setting** | **Outcome of interest** | **Standard intervention** | **Intervention adaptation** | **Reported Effectiveness*** |
| --- | --- | --- | --- | --- |
| Dreibelbis 2016  Bangladesh; Schools | Observed handwashing after toileting events | Handwashing station built first before hygiene education provision | **Sequencing** - Hygiene education provided at same time as handwashing station provision | Adaptation performed the same |
| Amon-Tanoh 2021  Côte d’Ivoire; Households | Observed handwashing with soap after toilet use | Handwashing station only | **Intensity -** Handwashing station combined with hygiene promotion activities (videos, Glo Germ demonstrations, posters) | Adaptation performed better |
| Biran 2020  Nigeria; Households | Observed handwashing with soap at key events (before eating or serving a meal, after defecation or latrine-use and after cleaning a child's bottom post-defecation) | Standard community-led total sanitation (CLTS) | **Intensity –** CLTS+, including additional discussions, skits, films, pledges, stickers, and report cards | Adaptation performed better |
| Freeman 2020  Kenya; Households | Households with a functional handwashing station | Standard program (THRIVE II) Training | **Intensity** - THRIVE II Training plus household visits from social workers and community health volunteers | Adaptation performed better |
| Nuhu 2019  Bangladesh; Households | Compliance with handwashing steps | Simple handwashing instructions | **Intensity** - Complex handwashing instructions | Adaptation performed the same |
| Sangalang 2021  Philippines; Schools | Handwashing practice score | WASH policy workshop for teachers and two health education sessions for students | **Intensity** - Medium-intensity (three 1-hr sessions) and high-intensity education sessions (four 1-hr sessions) | Adaptation performed worse |

***Notes:*** *Grey highlights are adaptations that performed better; *Reported effectiveness is determined if authors reported that the intervention was effective at improving hand hygiene outcomes*
