# Supplementary material for: Interventions to improve hand hygiene in community settings: a systematic review of theories, barriers and enablers, behaviour change techniques and hand hygiene station design features
Source: BMJ Glob Health. 2025 Sep 16;10(Suppl 7):e018928. doi: 10.1136/bmjgh-2025-018928 (PMC12443188; doi:10.1136/bmjgh-2025-018928)

**Interventions to improve hand hygiene in community settings: A systematic review of theories, barriers and enablers, behavior change techniques, and hand hygiene station design features**

*Authors*

Sridevi K. Prasad^1^ 0000-0003-0457-9534

Jedidiah S. Snyder^2^ 0000-0002-7688-4450

Erin LaFon^2^

Lilly A. O’Brien^2^ 0009-0004-1987-3706

Hannah Rogers^3^ 0000-0002-9515-1439

Oliver Cumming^4,5^ 0000-0002-5074-8709

Joanna Esteves Mills^5^

Bruce Gordon ^5^

Marlene Wolfe^2^ 0000-0002-6476-0450

Matthew C. Freeman^2^ 0000-0002-1517-2572

Bethany A. Caruso^1*^ 0000-0001-9738-9857

1 Hubert Department of Global Health, Rollins School of Public Health, Emory University, Atlanta, GA, USA; [bcaruso@emory.edu](mailto:bcaruso@emory.edu) (BAC); [sridevi.prasad@emory.edu](mailto:sridevi.prasad@emory.edu) (SKP)

2 Gangarosa Department of Environmental Health, Rollins School of Public Health, Emory University, Atlanta, GA, USA; [matthew.freeman@emory.edu](mailto:matthew.freeman@emory.edu) (MCF); [marlene.wolfe@emory.edu](mailto:marlene.wolfe@emory.edu) (MW) [jedidiah.snyder@emory.edu](mailto:jedidiah.snyder@emory.edu) (JSS); [lilly.obrien@emory.edu](mailto:lilly.obrien@emory.edu) (LAO); [erin.lafon@emory.edu](mailto:erin.lafon@emory.edu) (EL)

3 Woodruff Health Sciences Center Library, Emory University, Atlanta, GA, USA; [hannah.rogers@emory.edu](mailto:hannah.rogers@emory.edu) (HR)

4 Department of Disease Control, London School of Hygiene and Tropical Medicine, London, UK; [oliver.cumming@lshtm.ac.uk](mailto:oliver.cumming@lshtm.ac.uk) (OC)

5 Water, Sanitation, Hygiene and Health Unit, World Health Organization, Geneva, Switzerland; [estevesj@who.int](mailto:estevesj@who.int) (JEM); [gordonb@who.int](mailto:gordonb@who.int) (BG)

*Corresponding author: Bethany A. Caruso [bcaruso@emory.edu](mailto:bcaruso@emory.edu)

Emory University, Rollins School of Public Health, 1518 Clifton Rd, Atlanta, GA 30322

***Supplemental Text and Figure:***

Among all hand hygiene interventions studies, we report if and how handwashing practices are reported to vary by population groups, risk scenarios, and over time (**S17 Figure**). Among the 223 studies, 240 population groups were engaged, with some studies targeting multiple groups. The largest proportion of studies focused on children (83; 35%), of which 69 (83%) were effective.

Ten distinct vulnerable groups were identified across 34 studies: pregnant women (13; 38%), individuals with specific illnesses or risk factors (6; 18%), persons with disabilities (4; 12%), people of low income (2; 6%), children who are orphans or in foster care (2; 6%), people who are refugees or displaced (2; 6%), people belonging to specific ethnic or religious groups (2; 6%), people who inject drugs (1; 3%), newborns (1; 3%), and people who are immigrants or migrants (1; 3%). Sixty-two percent (8/13) of studies that focused on pregnant women reported interventions to be effective, and 83% (5/6) of studies that focused on individuals with specific illnesses or risk factors reported interventions to be effective.

Six risk scenarios were explored across studies, most focused on infectious disease: Flu (8; 47%), COVID-19 (4; 24%), internal displacement (2; 12%), hurricane (1; 6%), earthquake (1; 6%), and Ebola (1; 6%). Seventy-five percent (6/8) of studies that focused on Flu reported interventions to be effective, and 75% (3/4) of studies that focused on COVID-19 reported interventions to be effective.

Across studies, a minority collected data post-intervention at two or more time points: two time points (30); three (13); four (3), five (2), six (2). Seventy-nine percent (24/30) of studies that collected data two times post-intervention reported interventions to be effective, and 100% (13/13) of studies that collected data three times post-intervention reported interventions to be effective.

**S17 Figure. Proportion of studies reporting effective interventions stratified by (A) key population groups, (B) vulnerable groups, (C) risk scenarios, and (D) data points collected post-intervention.**


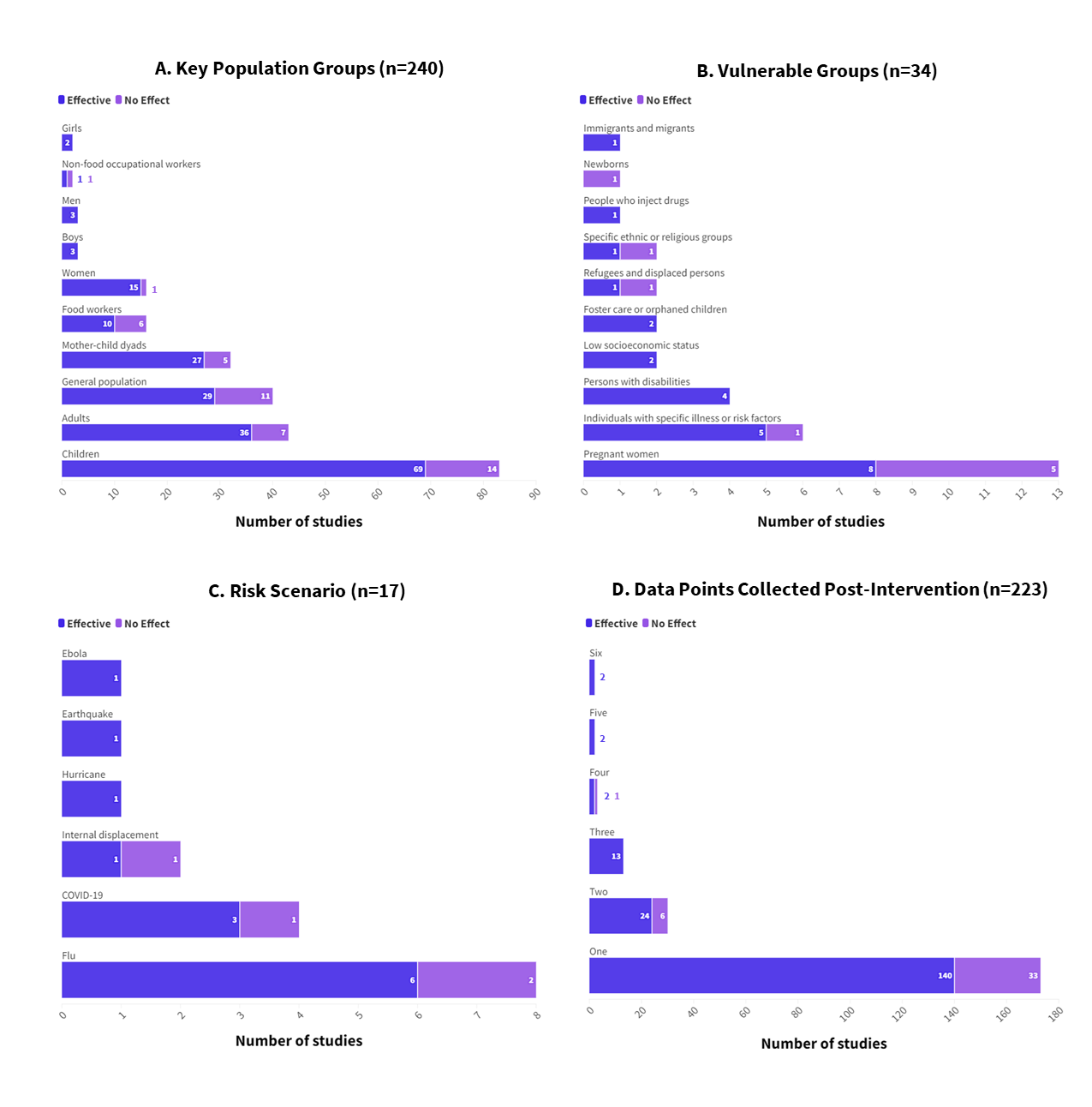

Supplement: online supplemental file 17 [file bmjgh-10-Suppl_7-s017.docx]
