# Supplementary material for: Interventions to improve hand hygiene in community settings: a systematic review of theories, barriers and enablers, behaviour change techniques and hand hygiene station design features
Source: BMJ Glob Health. 2025 Sep 16;10(Suppl 7):e018928. doi: 10.1136/bmjgh-2025-018928 (PMC12443188; doi:10.1136/bmjgh-2025-018928)
Supplement: online supplemental file 18 [file bmjgh-10-Suppl_7-s018.docx]

**Interventions to improve hand hygiene in community settings: A systematic review of theories, barriers and enablers, behavior change techniques, and hand hygiene station design features**

*Authors*

Sridevi K. Prasad^1^ 0000-0003-0457-9534

Jedidiah S. Snyder^2^ 0000-0002-7688-4450

Erin LaFon^2^

Lilly A. O’Brien^2^ 0009-0004-1987-3706

Hannah Rogers^3^ 0000-0002-9515-1439

Oliver Cumming^4,5^ 0000-0002-5074-8709

Joanna Esteves Mills^5^

Bruce Gordon ^5^

Marlene Wolfe^2^ 0000-0002-6476-0450

Matthew C. Freeman^2^ 0000-0002-1517-2572

Bethany A. Caruso^1*^ 0000-0001-9738-9857

1 Hubert Department of Global Health, Rollins School of Public Health, Emory University, Atlanta, GA, USA; [bcaruso@emory.edu](mailto:bcaruso@emory.edu) (BAC); [sridevi.prasad@emory.edu](mailto:sridevi.prasad@emory.edu) (SKP)

2 Gangarosa Department of Environmental Health, Rollins School of Public Health, Emory University, Atlanta, GA, USA; [matthew.freeman@emory.edu](mailto:matthew.freeman@emory.edu) (MCF); [marlene.wolfe@emory.edu](mailto:marlene.wolfe@emory.edu) (MW) [jedidiah.snyder@emory.edu](mailto:jedidiah.snyder@emory.edu) (JSS); [lilly.obrien@emory.edu](mailto:lilly.obrien@emory.edu) (LAO); [erin.lafon@emory.edu](mailto:erin.lafon@emory.edu) (EL)

3 Woodruff Health Sciences Center Library, Emory University, Atlanta, GA, USA; [hannah.rogers@emory.edu](mailto:hannah.rogers@emory.edu) (HR)

4 Department of Disease Control, London School of Hygiene and Tropical Medicine, London, UK; [oliver.cumming@lshtm.ac.uk](mailto:oliver.cumming@lshtm.ac.uk) (OC)

5 Water, Sanitation, Hygiene and Health Unit, World Health Organization, Geneva, Switzerland; [estevesj@who.int](mailto:estevesj@who.int) (JEM); [gordonb@who.int](mailto:gordonb@who.int) (BG)

*Corresponding author: Bethany A. Caruso [bcaruso@emory.edu](mailto:bcaruso@emory.edu)

Emory University, Rollins School of Public Health, 1518 Clifton Rd, Atlanta, GA 30322

***Supplementary file 18:***

Full References of included studies – linked studies are represented by ****** under the primary extracted study

1. Abbot JM, Policastro P, Bruhn C, Schaffner DW, Byrd-Bredbenner C. Development and evaluation of a university campus-based food safety media campaign for young adults. J Food Prot. 2012;75:1117–24.

2. Aboud FE, Sadika A. A cluster-randomized evaluation of a responsive stimulation and feeding intervention in Bangladesh. Pediatrics. 2011;127:e1191–7.

3. Adam B, Schmidt WP, Varadharajan KS, Divya R, Raja K, Greenland K, et al. Effect of a behaviour-change intervention on handwashing with soap in India (SuperAmma): a cluster-randomised trial. Lancet Global Health. 2014;2:e145–52.

4. Advaita S, Chaithra V, Anuradha B, Deepa B. Effective behavior change in hand hygiene practices among boys residing at foster care home in Bengaluru city: an interventional study. Indian Journal of Medical Sciences. 2021;73:82–7.

5. Aibana O, Franke MF, Teng JE, Hilaire J, Raymond M, Ivers LC. Cholera vaccination campaign contributes to improved knowledge regarding cholera and improved practice relevant to waterborne disease in rural Haiti. PLoS Negl Trop Dis. 2013;7:e2576.

******Childs L, François J, Choudhury A, Wannemuehler K, Dismer A, Hyde TB, et al. Evaluation of Knowledge and Practices Regarding Cholera, Water Treatment, Hygiene, and Sanitation Before and After an Oral Cholera Vaccination Campaign-Haiti, 2013-2014. Am J Trop Med Hyg. 2016;95:1305–13.

6. Aiello AE, Perez V, Coulborn RM, Davis BM, Uddin M, Monto AS. Facemasks, hand hygiene, and influenza among young adults: a randomized intervention trial. PLoS ONE. 2012;7:e29744.

7. Akina S, Schindler C, Odermatt P, Gerold J, Erismann S, Subodh S, et al. Nutritional and health status of children 15 months after integrated school garden, nutrition, and water, sanitation and hygiene interventions: a cluster-randomised controlled trial in Nepal. BMC Public Health. 2020;20:(03 February 2020).

******Erismann S, Diagbouga S, Schindler C, Odermatt P, Knoblauch AM, Gerold J, et al. School Children’s Intestinal Parasite and Nutritional Status One Year after Complementary School Garden, Nutrition, Water, Sanitation, and Hygiene Interventions in Burkina Faso. Am J Trop Med Hyg. 2017;97:904–13.

8. Akuokoasibey A, McPherson HJ. ASSESSING HYGIENE AND HEALTH-RELATED IMPROVEMENTS OF A RURAL WATER-SUPPLY AND SANITATION PROGRAM IN NORTHERN GHANA. Natural Resources Forum. 1994;18:49–54.

9. Alam N, Wojtyniak B, Henry FJ, Rahaman MM. Mothers’ personal and domestic hygiene and diarrhoea incidence in young children in rural Bangladesh. Int J Epidemiol. 1989;18:242–7.

10. Alexander AM, Mohan VR, Muliyil J, Dorny P, Rajshekhar V. Changes in knowledge and practices related to taeniasis/cysticercosis after health education in a south Indian community. International Health. 2012;4:164–9.

11. Ali E, Benedetti G, Van den BerghId R, Halford A, Bawo L, Massaquoiid M, et al. Distribution of household disinfection kits during the 2014-2015 ebola virus outbreak in monrovia, liberia: The MSF experience. PLoS Neglected Tropical Diseases. 2020;14:1–13.

12. Alkon A, Bernzweig J, To K, Wolff M, Mackie JF. Child care health consultation improves health and safety policies and practices. Acad Pediatr. 2009;9:366–70.

13. Almazan JU. Participatory Hygiene and Sanitation Transformation (PHAST) in a Remote and Isolated Community in Samar Province, Philippines. Curr Health Sci J. 2014;40:233–43.

14. Amon-Tanoh MA, McCambridge J, Blon PK, Kouamé HA, Nguipdop-Djomo P, Biran A, et al. Effects of a social norm-based handwashing intervention including handwashing stations, and a handwashing station-only intervention on handwashing with soap in urban Côte d’Ivoire: a cluster randomised controlled trial. Lancet Glob Health. 2021;9:e1707–18.

15. Andrade EL, Bingenheimer JB, Edberg MC, Zoerhoff KL, Putzer EM. Evaluating the effectiveness of a community-based hygiene promotion program in a rural Salvadoran setting. Glob Health Promot. 2019;26:69–80.

16. Anu R, Routh JA, Anagha L, Chemey E, Ayers T, Gunda AW, et al. Diffusion of handwashing knowledge and water treatment practices from mothers in an antenatal hygiene promotion program to nonpregnant friends and relatives, Machinga District, Malawi. International Quarterly of Community Health Education. 2018;39:63–9.

17. Appiah-Brempong E, Newton S, Harris MJ, Gulis G. Effect of a theory-based hand hygiene educational intervention for enhancing behavioural outcomes in Ghanaian schools: a cluster-randomised controlled trial. Int J Public Health. 2020;65:99–109.

18. Ar F, Kiliç E, Akbay Yarpuzlu A. A study of learning assessment of personal hygiene skills of mentally retarded individuals in drop-in day care services. Turkish Journal of Medical Sciences. 2008;38:447–53.

19. Ara G, Sanin KI, Khanam M, Sarker MSA, Tofail F, Nahar B, et al. A comprehensive intervention package improves the linear growth of children under 2-years-old in rural Bangladesh: a community-based cluster randomized controlled trial. Scientific Reports. 2022;12:21962.

20. Aragie S, Tadesse W, Dagnew A, Hailu D, Dubie M, Wittberg DM, et al. Changing hygiene behaviours: A cluster-randomized trial, Ethiopia. Bulletin of the World Health Organization. 2021;99:762-772A.

21. Arbianingsih null, Utario Y, Rustina Y, Krianto T, Ayubi D. Arbi Care application increases preschool children’s hand-washing self-efficacy among preschool children. Enfermeria Clinica. 2018;28:27–30.

22. Arıkan D, Gürarslan Baş N, Kurudirek F, Baştopcu A, Uslu H. The Effect of Therapeutic Clowning on Handwashing Technique and Microbial Colonization in Preschool Children. J Nurs Scholarsh. 2018;50:441–50.

23. Arnold B, Arana B, Mäusezahl D, Hubbard A, Colford Jr JM. Evaluation of a pre-existing, 3-year household water treatment and handwashing intervention in rural Guatemala. International Journal of Epidemiology. 2009;38:1651–61.

24. Ashraf S, Nizame FA, Mahfuza I, Dutta NC, Dalia Y, Sadika A, et al. Nonrandomized trial of feasibility and acceptability of strategies for promotion of soapy water as a handwashing agent in rural Bangladesh. American Journal of Tropical Medicine and Hygiene. 2017;96:421–9.

25. Ashtarian H, Shafiee F, Khezeli M, Almasi A, Rajati F, Zare F. Comparing the Effect of Lecture and Practical Demonstration Methods on Hand Hygiene in Elementary Students. Journal of Basic and Clinical Health Sciences. 2020;4:271–5.

26. Ashutosh S, Mubashir A. Improving hand washing among school children: an educational intervention in south India. Al Ameen Journal of Medical Sciences. 2015;8:81–5.

27. Au WH, Suen LKP, Kwok YL. Handwashing programme in kindergarten: a pilot study. Health Education. 2010;110:5–16.

28. Ãzyazıcıoğlu N, Ãnsal A, Sezgin S. The Effects of Toilet and Genital Hygiene Education on High School Students’ Behavior. International Journal of Caring Sciences. 2011;4:120–5.

29. Bai X, Li X, Yan D, Yang H, Tu K. Effects of Micro Architectural Environmental Interventions on Handwashing Compliance of Adolescents: A School-Based Intervention Trial. Herd. 2022;15:81–95.

30. Bajracharya D. Myanmar experiences in sanitation and hygiene promotion: lessons learned and future directions. Int J Environ Health Res. 2003;13 Suppl 1:S141-52.

31. Bickford AR, Lee JH, Borzekowski DLG. Cleaner, Happier, Healthier: Sesame Workshop’s Water, Sanitation, and Hygiene Intervention among Low-Income Groups in Bangladesh and India. Frontiers in Communication [Internet]. 2017;2. Available from: <https://www.scopus.com/inward/record.uri?eid=2-s2.0-85096139069&doi=10.3389%2ffcomm.2017.00020&partnerID=40&md5=6b2ac6dcaabc71b1a47cca73be5dbd77>

32. Bieri FA, Gray DJ, Williams GM, Raso G, Li YS, Yuan L, et al. Health-education package to prevent worm infections in Chinese schoolchildren. New England Journal of Medicine. 2013;368:1603‐1612.

33. Biran A, Schmidt WP, Wright R, Jones T, Seshadri M, Isaac P, et al. The effect of a soap promotion and hygiene education campaign on handwashing behaviour in rural India: a cluster randomised trial. Tropical Medicine & International Health. 2009;14:1303‐1314.

34. Biran A, White S, Awe B, Greenland K, Akabike K, Chuktu N, et al. A cluster-randomised trial to evaluate an intervention to promote handwashing in rural Nigeria. International Journal of Environmental Health Research. 2020;1–16.

35. Biswas D, Ahmed M, Roguski K, Ghosh PK, Parveen S, Nizame FA, et al. Effectiveness of a Behavior Change Intervention with Hand Sanitizer Use and Respiratory Hygiene in Reducing Laboratory-Confirmed Influenza among Schoolchildren in Bangladesh: a Cluster Randomized Controlled Trial. American Journal of Tropical Medicine and Hygiene. 2019;101:1446‐1455.

36. Blanton E, Ombeki S, Oluoch GO, Mwaki A, Wannemuehler K, Quick R. Evaluation of the role of school children in the promotion of point-of-use water treatment and handwashing in schools and households--Nyanza Province, Western Kenya, 2007. Am J Trop Med Hyg. 2010;82:664–71.

37. Bosomprah S, Beach LB, Beres LK, Newman J, Kapasa K, Rudd C, et al. Findings from a comprehensive diarrhoea prevention and treatment programme in Lusaka, Zambia. BMC Public Health. 2016;16:475.

38. Bowen A, Mubina A, Ayers T, Tobery T, Tariq M, Luby SP. Sustained improvements in handwashing indicators more than 5 years after a cluster-randomised, community-based trial of handwashing promotion in Karachi, Pakistan. Tropical Medicine and International Health. 2013;18:259–67.

39. Briceño B, Coville A, Gertler P, Martinez S. Are there synergies from combining hygiene and sanitation promotion campaigns: Evidence from a large-scale cluster-randomized trial in rural Tanzania. PLoS ONE. 2017;12:e0186228.

40. Briere EC, Ryman TK, Cartwright E, Russo ET, Wannemuehler KA, Nygren BL, et al. Impact of integration of hygiene kit distribution with routine immunizations on infant vaccine coverage and water treatment and handwashing practices of Kenyan mothers. J Infect Dis. 2012;205 Suppl 1:S56-64.

41. Bulled N, Poppe K, Ramatsisti K, Sitsula L, Winegar G, Gumbo J, et al. Assessing the environmental context of hand washing among school children in Limpopo, South Africa. Water Int. 2017;42:568–84.

42. Burke A, Dworkinn MS. High school students as the target of an integrated food safety educational intervention: successful results of a pilot study. Food Protection Trends. 2016;36:206–20.

43. Burns J, Maughan-Brown B, Mouzinho Â. Washing with hope: evidence of improved handwashing among children in South Africa from a pilot study of a novel soap technology. BMC Public Health. 2018;18:709.

44. Cairncross S, Shordt K, Zacharia S, Govindan BK. What causes sustainable changes in hygiene behaviour? A cross-sectional study from Kerala, India. Soc Sci Med. 2005;61:2212–20.

45. Capps KP, Updegraff JA, Foust JL, O’Brien AG, Taber JM. Field Experiment of Signs Promoting Hand Hygiene During the COVID-19 Pandemic. Health Psychology. 2022;41:826–32.

46. Carabin H, Gyorkos TW, Soto JC, Joseph L, Payment P, Collet JP. Effectiveness of a training program in reducing infections in toddlers attending day care centers. Epidemiology. 1999;10:219–27.

47. Cardinale Lagomarsino B, Gutman M, Freira L, Lanzalot ML, Lauletta M, Malchik LE, et al. PEER PRESSURE: EXPERIMENTAL EVIDENCE FROM RESTROOM BEHAVIOR. Economic Inquiry. 2017;55:1579–84.

48. Chard AN, Freeman MC. Design, Intervention Fidelity, and Behavioral Outcomes of a School-Based Water, Sanitation, and Hygiene Cluster-Randomized Trial in Laos. Int J Environ Res Public Health. 2018;15.

49. Contzen N, Meili I. Changing handwashing behavior in southern Ethiopia: A longitudinal study on infrastructural and commitment interventions. Psychology & Health. 2013;28:189–90.

50. Costa P, Ermini T, Sigaud CH de S. Effects of an educational playful intervention on nasal hygiene behaviors of preschoolers: a quasi-experimental study. Health Promotion Perspectives. 2019;9:50–4.

51. Cowling BJ, Chan KH, Fang VJ, Cheng CK, Fung RO, Wai W, et al. Facemasks and hand hygiene to prevent influenza transmission in households: a cluster randomized trial. Annals of Internal Medicine. 2009;151:437‐446.

52. Croghan E. Preventing sickness absence from early years education. British Journal of School Nursing. 2008;3:230–3.

53. Curtis V, Kanki B, Cousens S, Diallo I, et al. Evidence of behaviour change following a hygiene promotion programme in Burkina Faso. World Health Organization Bulletin of the World Health Organization. 2001;79:518–27.

54. Davis J, Pickering AJ, Rogers K, Mamuya S, Boehm AB. The effects of informational interventions on household water management, hygiene behaviors, stored drinking water quality, and hand contamination in peri-urban Tanzania. Am J Trop Med Hyg. 2011;84:184–91.

55. Davis OL, Fante RM, Jacobi LL. The effectiveness of sign prompts to increase hand washing behaviors in restrooms. North American Journal of Psychology. 2013;15:565–76.

56. Ditai J, Abeso J, Odeke NM, Mobbs N, Dusabe-Richards J, Mudoola M, et al. BabyGel pilot: a pilot cluster randomised trial of the provision of alcohol handgel to postpartum mothers to prevent neonatal and young infant infection-related morbidity in the community. Pilot and Feasibility Studies [Internet]. 2019;5. Available from: <https://www.cochranelibrary.com/central/doi/10.1002/central/CN-02095104/full>

57. Dreibelbis R, Kroeger A, Kamal H, Mohini V, Ram PK. Behavior change without behavior change communication: nudging handwashing among primary school students in Bangladesh. International Journal of Environmental Research and Public Health. 2016;13:129.

******Grover E, Hossain MK, Uddin S, Venkatesh M, Ram PK, Dreibelbis R. Comparing the behavioral impact of a nudge based handwashing intervention to high intensity hygiene education: a cluster-randomised trial in rural Bangladesh. Trop Med Int Health. 2018;23:10–25.

58. Duijster D, Buxton H, Benzian H, Dimaisip-Nabuab J, Monse B, Volgenant C, et al. Impact of a school-based water, sanitation and hygiene programme on children’s independent handwashing and toothbrushing habits: a cluster-randomised trial. Int J Public Health. 2020;65:1699–709.

59. Early E, Battle K, Cantwell E, English J, Lavin JE, Larson E. Effect of several interventions on the frequency of handwashing among elementary public school children. Am J Infect Control. 1998;26:263–9.

60. Ebuehi OM. Using community-based interventions to improve disease prevention practices of caregivers of under-5s in Ile-Ife, south-western Nigeria. SAJCH South African Journal of Child Health. 2010;4:32–6.

61. Edward A, Jung Y, Chhorvann C, Ghee AE, Chege J. Association of mother’s handwashing practices and pediatric diarrhea: evidence from a multi-country study on community oriented interventions. J Prev Med Hyg. 2019;60:E93-e102.

62. Ercan Oruc D, Pokharel S, Anantheswaran RC, Bucknavage MW, Gourama H, Shanina O, et al. A comprehensive food safety short course (FSSC) improves food safety knowledge, behaviors, attitudes, and skills of Ukrainian participants. Journal of Food Science Education. 2020;19:263–77.

63. Ercumen A, Mertens A, Arnold BF, Benjamin-Chung J, Hubbard AE, Ahmed MA, et al. Effects of Single and Combined Water, Sanitation and Handwashing Interventions on Fecal Contamination in the Domestic Environment: a Cluster-Randomized Controlled Trial in Rural Bangladesh. Environmental Science & Technology. 2018;52:12078‐12088.

******Parvez SM, Azad R, Rahman M, Unicomb L, Ram PK, Naser AM, et al. Achieving optimal technology and behavioral uptake of single and combined interventions of water, sanitation hygiene and nutrition, in an efficacy trial (WASH benefits) in rural Bangladesh. Trials [Internet]. 2018;19. Available from: <https://www.cochranelibrary.com/central/doi/10.1002/central/CN-01617390/full>

******Parvez SM, Rahman MJ, Rashidul A, Mahbubur R, Unicomb L, Ashraf S, et al. Achieving equitable uptake of handwashing and sanitation by addressing both supply and demand-based constraints: findings from a randomized controlled trial in rural Bangladesh. International Journal for Equity in Health. 2021;20.

******Luby SP, Rahman M, Arnold BF, Unicomb L, Ashraf S, Winch PJ, et al. Effects of water quality, sanitation, handwashing, and nutritional interventions on diarrhoea and child growth in rural Bangladesh: a cluster randomised controlled trial. The Lancet Global Health. 2018;6:e302–15.

64. Eun-Joo KIM, Andrew JP, Nam EK, Woo-Kyoung KIM, Young-Soon KIM, Hyun-Kyung M, et al. The effects of food safety education on adolescents’ hand hygiene behavior: an analysis of stages of change. Nutrition Research and Practice. 2012;169–74.

65. Evans Jr MW, Ramcharan M, Ndetan H, Floyd R, Globe G, Pfefer M, et al. Hand Hygiene and Treatment Table Sanitizing in Chiropractic Teaching Institutions: Results of an Education Intervention to Increase Compliance. Journal of Manipulative and Physiological Therapeutics. 2009;32:469–76.

66. Farhana S, Leanne U, Mahbubur R, Shahjahan A, Southern DL, Dalia Y, et al. Pilot of a low-cost elementary school handwashing intervention in Bangladesh: acceptability, feasibility, and potential for sustainability. American Journal of Tropical Medicine and Hygiene. 2022;106:239–49.

67. Ford EW, Boyer BT, Menachemi N, Huerta TR. Increasing hand washing compliance with a simple visual cue. Am J Public Health. 2014;104:1851–6.

68. Freeman MC, Delea MG, Snyder JS, Garn JV, Mulusew B, Caruso BA, et al. The impact of a demand-side sanitation and hygiene promotion intervention on sustained behavior change and health in Amhara, Ethiopia: a cluster-randomized trial. PLoS Global Public Health. 2022;2.

69. Freeman MC, Ellis AS, Ogutu EA, Caruso BA, Linabarger M, Micek K, et al. Impact of a demand-side integrated WASH and nutrition community-based care group intervention on behavioural change: a randomised controlled trial in western Kenya. BMJ Glob Health [Internet]. 2020;5. Available from: <https://gh.bmj.com/content/bmjgh/5/11/e002806.full.pdf>

70. Friedrich MND, Kappler A, Mosler H-J. Enhancing handwashing frequency and technique of primary caregivers in Harare, Zimbabwe: A cluster-randomized controlled trial using behavioral and microbial outcomes. Social Science & Medicine. 2018;196:66–76.

71. Galiani S, Gertler P, Ajzenman N, Orsola-Vidal A. Promoting Handwashing Behavior: The Effects of Large-scale Community and School-level Interventions. Health Econ. 2016;25:1545–59.

72. Gautam OP, Schmidt WP, Cairncross S, Cavill S, Curtis V. Trial of a Novel Intervention to Improve Multiple Food Hygiene Behaviors in Nepal. Am J Trop Med Hyg. 2017;96:1415–26.

73. Gedamu B, Hailu M, Fasil T. Effects of community-led total sanitation and hygiene implementation on diarrheal diseases prevention in children less than five years of age in South western Ethiopia: a quasi- experimental study. PLoS ONE. 2022;17.

******Gezahegn M, Kloos H, Metadel A. Prevalence of and factors associated with acute diarrhea among children under five in rural areas in Ethiopia with and without implementation of community-led total sanitation and hygiene. BMC Pediatrics. 2022;22:(21 March 2022).

74. Geller ES, Eason SL, Phillips JA, Pierson MD. Interventions to improve sanitation during food preparation. Journal of Organizational Behavior Management. 1980;2:229–40.

75. George CM, Biswas S, Jung D, Perin J, Parvin T, Monira S, et al. Psychosocial Factors Mediating the Effect of the CHoBI7 Intervention on Handwashing With Soap: A Randomized Controlled Trial. Health Education & Behavior. 2017;44:613–25.

******Bhuyian MSI, Perin J, Endres K, Fatema Z, Jahed M, Tahmina P, et al. Reduced diarrhea prevalence and improvements in handwashing with soap and stored drinking water quality associated with diarrheal disease awareness measured by interactive voice response messages in the CHoBI7 mobile health program. American Journal of Tropical Medicine and Hygiene. 2023;108:530–5.

******Burrowes V, Perin J, Monira S, Sack D, Rashid MU, Mahamud T, et al. Risk factors for household transmission of Vibrio Cholerae In Dhaka, Bangladesh (chobi7 trial). American Journal of Tropical Medicine and Hygiene. 2017;97:188‐.

76. Gimaiyo G, McManus J, Yarri M, Singh S, Trevett A, Moloney G, et al. Can child-focused sanitation and nutrition programming improve health practices and outcomes? Evidence from a randomised controlled trial in Kitui County, Kenya. BMJ Glob Health. 2019;4:e000973.

77. Goel AD, Gosain M, Amarchand R, Sharma H, Rai S, Kapoor SK, et al. Effectiveness of a Quality Improvement Program Using Difference-in-Difference Analysis for Home Based Newborn Care – Results of a Community Intervention Trial. Indian Journal of Pediatrics. 2019;86:1028–35.

78. Goel S, Chandrashekar BR. Evaluating the efficacy of handwashing demonstration on hand hygiene among school students - An interventional study. J Educ Health Promot. 2020;9:226.

79. Grace D, Dipeolu M, Olawoye J, Ojo E, Odebode S, Agbaje M, et al. Evaluating a group-based intervention to improve the safety of meat in Bodija market, Ibadan, Nigeria. Trop Anim Health Prod. 2012;44 Suppl 1:S61-6.

80. Greene LE, Freeman MC, Akoko D, Saboori S, Moe C, Rheingans R. Impact of a school-based hygiene promotion and sanitation intervention on pupil hand contamination in Western Kenya: a cluster randomized trial. Am J Trop Med Hyg. 2012;87:385–93.

******Freeman MC, Greene LE, Dreibelbis R, Saboori S, Muga R, Brumback B, et al. Assessing the impact of a school-based water treatment, hygiene and sanitation programme on pupil absence in Nyanza Province, Kenya: A cluster-randomized trial. Tropical Medicine and International Health. 2012;17:380–91.

81. Greenland K, Chipungu J, Curtis V, Schmidt WP, Siwale Z, Mudenda M, et al. Multiple behaviour change intervention for diarrhoea control in Lusaka, Zambia: a cluster randomised trial. Lancet Glob Health. 2016;4:e966–77.

82. Grover E, Hossain MK, Saker U, Mohini V, Ram PK, Dreibelbis R. Comparing the behavioural impact of a nudge-based handwashing intervention to high-intensity hygiene education: a cluster-randomised trial in rural Bangladesh. Tropical Medicine and International Health. 2018;23:10–25.

83. Guo N, Ma H, Deng J, Ma Y, Huang L, Guo R, et al. Effect of hand washing and personal hygiene on hand food mouth disease: A community intervention study. Medicine (Baltimore). 2018;97:e13144.

84. Hanson C, Allen E, Fullmer M, O’brien R, Dearden K, Garn J, et al. A national communication campaign in Indonesia is associated with improved WASH-related knowledge and behaviors in Indonesian mothers. International Journal of Environmental Research and Public Health [Internet]. 2020;17. Available from: <https://www.embase.com/search/results?subaction=viewrecord&id=L2004441274&from=export>

85. Her E, Behnke MSC, Almanza B. Does a water flow timer improve food handler hand washing practices in food service establishments? The effects of passive and indirect interventions. Journal of Environmental Health. 2019;81:8–13.

86. Hetherington E, Eggers M, Wamoyi J, Hatfield J, Manyama M, Kutz S, et al. Participatory science and innovation for improved sanitation and hygiene: process and outcome evaluation of project SHINE, a school-based intervention in Rural Tanzania. BMC Public Health. 2017;17:172.

87. Huang HC, Le N, Battle M, Villasenor JM, Maule L. Nudging Handwashing among Primary School Students in the Philippines: Evidence from a Cluster Randomized Trial. Am J Trop Med Hyg. 2021;105:1806–15.

88. Hurley KM, Phuka J, Kang Y, Ruel-Bergeron J, Buckland AJ, Mitra M, et al. A longitudinal impact evaluation of a comprehensive nutrition program for reducing stunting among children aged 6–23 months in rural Malawi. American Journal of Clinical Nutrition. 2021;114:248–56.

89. Hussam R, Rabbani A, Reggiani G, Rigol N. Rational Habit Formation: Experimental Evidence from Handwashing in India. American Economic Journal-Applied Economics. 2022;14:1–41.

90. Jafree SR, Muzammil A, Burhan SK, Bukhari N, Fischer F. Impact of a digital health literacy intervention and risk predictors for multimorbidity among poor women of reproductive years: Results of a randomized-controlled trial. Digital Health [Internet]. 2023;9. Available from: <https://www.scopus.com/inward/record.uri?eid=2-s2.0-85145500258&doi=10.1177%2f20552076221144506&partnerID=40&md5=1462175d818fab4419b0d0c28f49e585>

91. Jagals P, Nala NP, Tsubane TJ, Moabi M, Motaung KC. Measuring changes in water-related health and hygiene practices by developing-community households. Water Sci Technol. 2004;50:91–7.

92. Jetha Q, Bisserbe C, McManus J, Waldroop D, Naliponguit EC, Villasenor JM, et al. Can Social Motivators Improve Handwashing Behavior among Children? Evidence from a Cluster Randomized Trial of a School Hygiene Intervention in the Philippines. American Journal of Tropical Medicine and Hygiene. 2021;104:756–65.

******Bonnesen CT, Plauborg R, Denbæk AM, Due P, Johansen A. Process evaluation of a multi-component intervention to reduce infectious diseases and improve hygiene and well-being among school children: the Hi Five study. Health Educ Res. 2015;30:497–512.

******Denbæk AM, Andersen A, Bast LS, Bonnesen CT, Ersbøll AK, Due P, et al. Importance of implementation level when evaluating the effect of the Hi Five Intervention on infectious illness and illness-related absenteeism. Am J Infect Control. 2018;46:512–9.

93. Jinadu MK, Adegbenro CA, Esmai AO, Ojo AA, Oyeleye BA. Health promotion intervention for hygienic disposal of children’s faeces in a rural area of Nigeria. Health Education Journal. 2007;66:222–8.

94. Johnson HD, Sholcosky D, Gabello K, Ragni R, Ogonosky N. Sex differences in public restroom handwashing behavior associated with visual behavior prompts. Percept Mot Skills. 2003;97:805–10.

95. Judah G, Aunger R, Schmidt WP, Michie S, Granger S, Curtis V. Experimental pretesting of hand-washing interventions in a natural setting. Am J Public Health. 2009;99 Suppl 2:S405-11.

96. Kaewchana S, Simmerman M, Somrongthong R, Suntarattiwong P, Lertmaharit S, Chotipitayasunondh T. Effect of intensive hand washing education on hand washing behaviors in thai households with an influenza-positive child in urban Thailand. Asia Pac J Public Health. 2012;24:577–85.

97. Kajjura RB, Veldman FJ, Kassier SM. Effect of Nutrition Education on Knowledge, Complementary Feeding, and Hygiene Practices of Mothers With Moderate Acutely Malnourished Children in Uganda. Food Nutr Bull. 2019;40:221–30.

98. Kamm KB, Vujcic J, Nasreen S, Luby SP, Zaman K, El-Arifeen S, et al. Is pregnancy a teachable moment to promote handwashing with soap among primiparous women in rural Bangladesh? Follow-up of a randomised controlled trial. Tropical Medicine and International Health. 2016;21:1562–71.

99. Kang Y, Suh Y, Debele L, Juon H, Christian P. Effects of a community-based nutrition promotion programme on child feeding and hygiene practices among caregivers in rural Eastern Ethiopia. Public Health Nutrition. 2017;20:1461–72.

100. Kapadia-Kundu N, Storey D, Safi B, Trivedi G, Tupe R, Narayana G. Seeds of prevention: The impact on health behaviors of young adolescent girls in Uttar Pradesh, India, a cluster randomized control trial. Social Science and Medicine. 2014;120:169–79.

101. Kariuki JG, Magambo KJ, Njeruh MF, Muchiri EM, Nzioka SM, Kariuki S. Changing mother’s hygiene and sanitation practices in resource constrained communities: case study of Turkana District, Kenya. J Community Health. 2012;37:1185–91.

102. Karon AJ, Cronin AA, Cronk R, Hendrawan R. Improving water, sanitation, and hygiene in schools in Indonesia: A cross-sectional assessment on sustaining infrastructural and behavioral interventions. Int J Hyg Environ Health. 2017;220:539–50.

103. Kitsanapun A, Yamarat K. Evaluating the effectiveness of the “Germ-Free Hands” intervention for improving the hand hygiene practices of public health students. J Multidiscip Healthc. 2019;12:533–41.

104. Koehn HJ, Zheng S, Houser RF, O’Hara C, Rogers BL. Remuneration systems of community health workers in India and promoted maternal health outcomes: a cross-sectional study. BMC Health Serv Res. 2020;20:48.

105. Kumar A, Mahalakshmy T, Bitty T, Bharath N, Kanagarethinam R, Jayalakshmy R. How does school based hand-washing promotion program affect the handwashing behavior of students at the urban slums in Puducherry, South India? Mixed method design. International Journal of Medical Science and Public Health. 2018;7:874–8.

106. Labović SB, Joksimović I, Galić I, Knežević M, Mimović M. Food Safety Behaviours among Food Handlers in Different Food Service Establishments in Montenegro. Int J Environ Res Public Health. 2023;20.

107. Lange S, Barnard TG, Naicker N. The effect of a hand hygiene intervention on the behaviour, practices and health of parents of preschool children in South Africa. Perspect Public Health. 2022;142:338–46.

108. Langford R, Lunn P, Panter-Brick C. Hand-washing, subclinical infections, and growth: a longitudinal evaluation of an intervention in Nepali slums. Am J Hum Biol. 2011;23:621–9.

109. Langford R, Panter-Brick C. A health equity critique of social marketing: where interventions have impact but insufficient reach. Soc Sci Med. 2013;83:133–41.

110. Lapinski MK, Maloney EK, Braz M, Shulman HC. Testing the Effects of Social Norms and Behavioral Privacy on Hand Washing: A Field Experiment. Human Communication Research. 2013;39:21–46.

111. Lawson A, Vaganay-Miller M. The Effectiveness of a Poster Intervention on Hand Hygiene Practice and Compliance When Using Public Restrooms in a University Setting. Int J Environ Res Public Health. 2019;16.

112. Lee RLT, Leung C, Chen H, Lee PH, Kwok SWH. A cluster randomized controlled trial of a simplified 5‐step handwashing technique versus a conventional 7‐step handwashing technique among Chinese students with intellectual disabilities. Journal of Applied Research in Intellectual Disabilities. 2020;33:1090–9.

******Lee RLT, Leung C, Tong W, Chen H, Lee PH. Comparative efficacy of a simplified handwashing program for improvement in hand hygiene and reduction of school absenteeism among children with intellectual disability. AJIC - American Journal of Infection Control. 2015;43:907–12.

******Lee RL, Lee PH. To evaluate the effects of a simplified hand washing improvement program in schoolchildren with mild intellectual disability: a pilot study. Res Dev Disabil. 2014;35:3014–25.

113. Leventhal KS, DeMaria LM, Gillham JE, Andrew G, Peabody J, Leventhal SM. A psychosocial resilience curriculum provides the “missing piece” to boost adolescent physical health: A randomized controlled trial of Girls First in India. Soc Sci Med. 2016;161:37–46.

114. Lhakhang P, Lippke S, Knoll N, Schwarzer R. Evaluating brief motivational and self-regulatory hand hygiene interventions: a cross-over longitudinal design. BMC Public Health. 2015;15:79.

115. Liu X, Zhao Z, Hou W, Polinder S, Van Beeck EF, Zhang Z, et al. A multimodal intervention to improve hand hygiene compliance via social cognitive influences among kindergarten teachers in China. PLoS ONE. 2019;14:e0215824.

116. Locks LM, Nanama S, Addo OY, Albert B, Sandalinas F, Nanema A, et al. An integrated infant and young child feeding and small‐quantity lipid‐based nutrient supplementation programme in the Democratic Republic of Congo is associated with improvements in breastfeeding and handwashing behaviours but not dietary diversity. Maternal & Child Nutrition. 2019;15:N.PAG-N.PAG.

117. Lubna Y, Shamima A, Islam AMS, Rahman MDM, Hidechika A, Subrina J. Targeted interventions of ultra-poor women in rural Rangpur, Bangladesh: do they make a difference to appropriate cooking practices, food habits and sanitation? Journal of Biosocial Science. 2014;46:419–30.

118. Luby SP, Agboatwalla M, Raza A, Sobel J, Mintz ED, Baier K, et al. Microbiologic effectiveness of hand washing with soap in an urban squatter settlement, Karachi, Pakistan. Epidemiology and Infection. 2001;127:237‐244.

Luby SP, Aeboatwalla M, Bowen A, Kenah E, Sharker Y, Hoekstra RM. Difficulties in maintaining improved handwashing behavior, Karachi, Pakistan. American Journal of Tropical Medicine and Hygiene. 2009;81:140–5.

119. Luby SP, Kadir M, Sharker MAY, Yeasmin F, Unicomb L, Islam MS. A community-randomised controlled trial promoting waterless hand sanitizer and handwashing with soap, Dhaka, Bangladesh. Trop Med Int Health. 2010.

120. Machado RAM, Cutter CN. Training hard-to-reach Pennsylvanian cheesemakers about food safety, using a low-tech training tool. Food Protection Trends. 2018;38:266–83.

121. Mackert M, Liang M-C, Champlin S. “Think the sink:” Preliminary evaluation of a handwashing promotion campaign. American Journal of Infection Control. 2013;41:275–7.

122. Mahfuza I, Benjamin-Chung J, Sonia S, Leanne U, Alam M, Mahbubur R, et al. Effectiveness of mass media campaigns to improve handwashing-related behavior, knowledge, and practices in rural Bangladesh. American Journal of Tropical Medicine and Hygiene. 2021;104:1546–53.

******Johnston RB, Halder AK, Huda TMN, Akhter S, Amanullah al M, Huque MR, et al. Monitoring impacts of WASH interventions: the case of SHEWA-B. Loughborough: Water, Engineering and Development Centre (WEDC) Loughborough University of Technology; 2009. p. 352–9.

******Aluri KZ, Halder AK, Mahfuza I, Benjamin-Chung J, Monirul A, Shoab AK, et al. The effect of a large-scale water, sanitation and hygiene intervention in Bangladesh on knowledge, behaviour and health: findings from an endline programme evaluation. Tropical Medicine and International Health. 2022;27:913–24.

******Huda TMN, Unicomb L, Johnston RB, Halder AK, Yushuf Sharker MA, Luby SP. Interim evaluation of a large scale sanitation, hygiene and water improvement programme on childhood diarrhea and respiratory disease in rural Bangladesh. Social Science and Medicine. 2012;75:604–11.

123. Makata K, Ensink J, Ayieko P, Hansen C, Sichalwe S, Mngara J, et al. Hand hygiene intervention to optimise soil-transmitted helminth infection control among primary school children: the Mikono Safi cluster randomised controlled trial in northwestern Tanzania. BMC Medicine. 2021;19:125.

******Okello E, Kapiga S, Grosskurth H, Makata K, McHaro O, Kinungh’i S, et al. Factors perceived to facilitate or hinder handwashing among primary students: a qualitative assessment of the Mikono Safi intervention schools in NW Tanzania. BMJ Open. 2019;9:e030947.

******Sedekia Y, Kapiga S, McHaro O, Makata K, Torondel B, Dreibelbis R, et al. Does a school-based intervention to engage parents change opportunity for handwashing with soap at home? Practical experience from the Mikono Safi trial in Northwestern Tanzania. PLoS Negl Trop Dis. 2022;16:e0010438.

******Makata K, Kinung’hi S, Hansen C, Ayieko P, Sichalwe S, McHaro O, et al. Hand hygiene intervention to optimize helminth infection control: design and baseline results of Mikono Safi-An ongoing school-based cluster-randomised controlled trial in NW Tanzania. PLoS ONE. 2020;15:e0242240.

124. Malik FR, Asif R, Aimen N, Faryal K, Nayab A, Sonila K, et al. A quasi experimental study on hand hygiene practices among secondary school children in Khyber Pakhtunkhwa. JPMA, Journal of the Pakistan Medical Association. 2022;72:664–8.

125. Manaseki-Holland S, Manjang B, Hemming K, Martin JT, Bradley C, Jackson L, et al. Effects on childhood infections of promoting safe and hygienic complementary-food handling practices through a community-based programme: a cluster randomised controlled trial in a rural area of The Gambia. PLoS Medicine. 2021;18:e1003260.

126. Mane MA, Tata SH. A study to assess the effectiveness of hand hygiene technique among school children in Maharashtra, India. Asian Journal of Pharmaceutical Research and Health Care. 2017;9:174–9.

127. Mathew SM, Sujatha R. Effectiveness of Child To Child Approach on Practice of Hand Washing Among School Children in a Selected School at Mangalore. Nitte University Journal of Health Science. 2018;8:15–21.

128. Maughan C, Godwin S, Chambers D, Chambers EI. Recipe Modification Improves Food Safety Practices during Cooking of Poultry. J Food Prot. 2016;79:1436–9.

129. Mbakaya BC, Lee P, Lee L. Effect of a school-based hand hygiene program for Malawian children: a cluster randomized controlled trial. AJIC - American Journal of Infection Control. 2019;47:1460–4.

******Mbakaya BC, Lee RLT. Experiences of implementing hand hygiene for Malawian schoolchildren: a qualitative study. Int Nurs Rev. 2019;66:553–62.

130. McGuire-Wolfe C, Haiduven D, Hitchcock CD. A multifaceted pilot program to promote hand hygiene at a suburban fire department. Am J Infect Control. 2012;40:324–7.

131. Mendes PME, de Jesus Mateus LV, Costa P. Does a Playful Intervention Promote Hand Hygiene? Compliance and Educator’s Beliefs about Hand Hygiene at a Daycare Center. J Pediatr Nurs. 2020;51:e64–8.

132. Mezaache S, Briand-Madrid L, Rahni L, Poireau J, Branchu F, Moudachirou K, et al. A two-component intervention to improve hand hygiene practices and promote alcohol-based hand rub use among people who inject drugs: a mixed-methods evaluation. BMC Infect Dis. 2021;21:211.

133. Mohamed NA, Jamaluddin TZMT, Ismail Z, Rani MDM, Ramli S, Faroque H, et al. Healthy hands: Development and evaluation of a knowledge transfer program. Malaysian Journal of Medicine and Health Sciences. 2019;15:10–3.

134. Moll DM, McElroy RH, Sabogal R, Corrales LF, Gelting RJ. Health impact of water and sanitation infrastructure reconstruction programmes in eight Central American communities affected by Hurricane Mitch. J Water Health. 2007;5:51–65.

135. Morse T, Tilley E, Chidziwisano K, Malolo R, Musaya J. Health outcomes of an integrated behaviour-centred water, sanitation, hygiene and food safety intervention–a randomised before and after trial. International Journal of Environmental Research and Public Health [Internet]. 2020;17. Available from: <https://www.embase.com/search/results?subaction=viewrecord&id=L2004250583&from=export>

******Slekiene J, Chidziwisano K, Morse T. Does Poor Mental Health Impair the Effectiveness of Complementary Food Hygiene Behavior Change Intervention in Rural Malawi? Int J Environ Res Public Health. 2022;19.

******Chidziwisano K, Slekiene J, Hans-Joachim M, Morse T. Improving complementary food hygiene behaviors using the risk, attitude, norms, ability, and self-regulation approach in rural Malawi. American Journal of Tropical Medicine and Hygiene. 2020;102:1104–15.

136. Mott PJ, Sisk BW, Arbogast JW, Ferrazzano-Yaussy C, Bondi CAM, Sheehan JJ. Alcohol-based instant hand sanitizer use in military settings: A prospective cohort study of army basic trainees. Military Medicine. 2007;172:1170–6.

137. Nagapraveen V, Subramaniyan P, Praveenkumar BA, Arun G. Promotion of sanitation and hygiene in a rural area of South India: a community-based study. Journal of Family Medicine and Primary Care. 2016;5:587–92.

138. Nair N, Tripathy P, Sachdev HS, Pradhan H, Bhattacharyya S, Gope R, et al. Effect of participatory women’s groups and counselling through home visits on children’s linear growth in rural eastern India (CARING trial): a cluster-randomised controlled trial. The Lancet Global health. 2017;5:e1004‐e1016.

139. Naluonde T, Wakefield C, Markle L, Martin A, Tresphor C, Abdullah R, et al. A disruptive cue improves handwashing in school children in Zambia. Health Promot Int. 2019;34:e119–28.

140. Nandrup-Bus I. Mandatory handwashing in elementary schools reduces absenteeism due to infectious illness among pupils: a pilot intervention study. Am J Infect Control. 2009;37:820–6.

141. Newton-Lewis TA, Bahety G. Evaluating the effectiveness of Community Health Worker home visits on infant health: A quasi-experimental evaluation of Home Based Newborn Care Plus in India. J Glob Health. 2021;11:04060.

142. Nik Rosmawati NH, Wan Manan WM, Noor Izani NJ, Nik Nurain NH, Razlina AR. The effect of food safety education on handwashing practices in school canteens’ food handlers. Sains Malaysiana. 2018;47:2119–28.

143. Nuhu A, Sagerman DD, Nizame FA, Das KK, Md N, Yu J, et al. Effects of complexity of handwashing instructions on handwashing procedure replication in low-income urban slums in Bangladesh: a randomized non-inferiority field trial. Journal of Water, Sanitation and Hygiene for Development. 2019;9:416–28.

144. Öncü E, Vayısoğlu SK. Duration or technique to improve the effectiveness of children’ hand hygiene: A randomized controlled trial. Am J Infect Control. 2021;49:1395–401.

******Öncü E, Vayısoğlu SK, Lafci D, Yurtsever D, Bulut ER, Peker E. Comparison of Interactive Education Versus Fluorescent Concretization on Hand Hygiene Compliance Among Primary School Students: A Randomized Controlled Trial. J Sch Nurs. 2019;35:337–47.

145. Oruc DE, Pokharel S, Hirneisen AJ, Cutter CN. A food safety laboratory curriculum significantly improves knowledge, behaviors, attitudes, and handwashing skills of laboratory personnel in East and South Africa. Food Protection Trends. 2021;41:485–500.

146. Oswald WE, Hunter GC, Kramer MR, Leontsini E, Cabrera L, Lescano AG, et al. Provision of private, piped water and sewerage connections and directly observed handwashing of mothers in a peri-urban community of Lima, Peru. Trop Med Int Health. 2014;19:388–97.

147. Ozcan A, Ozdil K, Kaya SS, Sezer F. Hand Washing in Primary School Students Using “Demonstration, Puzzle, Dance, Song”: A Nursing Project Based on Multifaceted Skills Training. J Contin Educ Nurs. 2020;51:158–66.

148. Patel MK, Harris JR, Juliao P, Nygren B, Were V, Kola S. Impact of a hygiene curriculum and the installation of simple handwashing and drinking water stations in rural Kenyan primary schools on student health and hygiene practices. American Journal of Tropical Medicine and Hygiene. 2012;87:594‐601.

******Graves JM, Daniell WE, Harris JR, Obure AFXO, Quick R. Enhancing a safe water intervention with student-created visual aids to promote handwashing behavior in Kenyan primary schools. International Quarterly of Community Health Education. 2011;32:307–23.

******Con G la, Schilling K, Harris J, Person B, Owuor M, Ogange L, et al. Evaluation of student handwashing practices during a school-based hygiene program in rural Western Kenya, 2007. International Quarterly of Community Health Education. 2017;37:121–8.

149. Phuanukoonnon S, Namosha E, Kua L, Siba PM, Greenhill AR. Evaluation of a WASH intervention demonstrates the potential for improved hygiene practices in Hiri District, Central Province. P N G Med J. 2013;56:126–35.

150. Pickering AJ, Davis J, Blum AG, Scalmanini J, Oyier B, Okoth G, et al. Access to waterless hand sanitizer improves student hand hygiene behavior in primary schools in Nairobi, Kenya. Am J Trop Med Hyg. 2013;89:411–8.

151. Pickering AJ, Njenga SM, Steinbaum L, Swarthout J, Lin A, Arnold BF, et al. Effects of single and integrated water, sanitation, handwashing, and nutrition interventions on child soil-transmitted helminth and Giardia infections: a cluster-randomized controlled trial in rural Kenya. PLoS Medicine. 2019;16:e1002841.

******Christensen G, Dentz HN, Pickering AJ, Bourdier T, Arnold BF, Colford JM Jr, et al. Pilot cluster randomized controlled trials to evaluate adoption of water, sanitation, and hygiene interventions and their combination in rural western Kenya. Am J Trop Med Hyg. 2015;92:437–47.

152. Pinfold JV. Faecal contamination of water and fingertip-rinses as a method for evaluating the effect of low-cost water supply and sanitation activities on faeco-oral disease transmission. II. A hygiene intervention study in rural north-east Thailand. Epidemiol Infect. 1990;105:377–89.

******Pinfold JV. Analysis of different communication channels for promoting hygiene behaviour. Health Educ Res. 1999;14:629–39.

******Pinfold JV, Horan NJ. Measuring the effect of a hygiene behaviour intervention by indicators of behaviour and diarrhoeal disease. Trans R Soc Trop Med Hyg. 1996;90:366–71.

153. Pokharel S, Marcy JE, Neilan AM, Cutter CN. Development, dissemination, and assessment of a food safety systems management curriculum for agribusiness students in Armenia. Journal of Food Science Education. 2017;16:107–17.

154. Prado DB do, Bettoni AP, Correa VA, Abreu Filho BA de, Garcia LB, Tognim MCB, et al. Practice of hand hygiene in a university dining facility. Food Control. 2015;57:35–40.

155. Prasetyo DB, Sofyan L, Muchtar PA, Dewi DF. Nudging to handwash during the pandemic – The use of visual priming and salience. Analyses of Social Issues & Public Policy. 2022;22:836–56.

156. Ram PK, Begum F, Crabtree-Ide C, Uddin MR, Weaver AM, Dostogir Harun MG, et al. Waterless Hand Cleansing with Chlorhexidine during the Neonatal Period by Mothers and Other Household Members: findings from a Randomized Controlled Trial. American Journal of Tropical Medicine and Hygiene. 2020;103:2116‐2126.

157. Ram PK, Nasreen S, Kamm K, Allen J, Kumar S, Rahman MA, et al. Impact of an Intensive Perinatal Handwashing Promotion Intervention on Maternal Handwashing Behavior in the Neonatal Period: Findings from a Randomized Controlled Trial in Rural Bangladesh. Biomed Res Int. 2017;2017:6081470.

158. Ray SK, Zaman FA, Laskar NB. Hand washing practices in two communities of two states of Eastern India: an intervention study. Indian J Public Health. 2010;54:126–30.

159. Reyes Fernández B, Lippke S, Knoll N, Blanca Moya E, Schwarzer R. Promoting action control and coping planning to improve hand hygiene. BMC Public Health. 2015;15:964.

160. Riaz BK, Alim MA, Islam AS, Amin KB, Sarker MA, Hasan K, et al. Role of courtyard counselling meeting in improving household food safety knowledge and practices in Munshiganj district of Bangladesh. Nagoya J Med Sci. 2016;78:387–98.

161. Rissman L, Deavenport-Saman A, Corden MH, Zipkin R, Espinoza J. A pilot project: handwashing educational intervention decreases incidence of respiratory and diarrheal illnesses in a rural Malawi orphanage. Glob Health Promot. 2021;28:14–22.

162. Roberts KR, Barrett BB, Howells AD, Shanklin CW, Pilling VK, Brannon LA. Food safety training and foodservice employees’ knowledge and behavior. Food Protection Trends. 2008;28:252–60.

163. Roberts KR, Paez P, Sauer K, Alcorn M, Johnson DE. Impact of Training on Employees’ Handwashing Behaviors in School Nutrition Programs. J Acad Nutr Diet. 2022;

164. Rosen L, Manor O, Engelhard D, Brody D, Rosen B, Peleg H, et al. Can a handwashing intervention make a difference? Results from a randomized controlled trial in Jerusalem preschools. Prev Med. 2006;42:27–32.

******Rosen L, Zucker D, Brody D, Engelhard D, Manor O. The effect of a handwashing intervention on preschool educator beliefs, attitudes, knowledge and self-efficacy. Health Educ Res. 2009;24:686–98.

165. Routh JA, Loharikar A, Chemey E, Msoma A, Ntambo M, Mvula R, et al. Safe Water and Hygiene Integration with Human Immunodeficiency Virus and Antenatal Services: Leveraging Opportunities for Public Health Interventions and Improved Service Uptake. Am J Trop Med Hyg. 2018;98:1234–41.

166. Russo ET, Sheth A, Menon M, Wannemuehler K, Weinger M, Kudzala AC, et al. Water treatment and handwashing behaviors among non-pregnant friends and relatives of participants in an antenatal hygiene promotion program in Malawi. Am J Trop Med Hyg. 2012;86:860–5.

167. Rutter S, Stones C, Wood J, Macduff C, Gomez-Escalada M. Effectiveness and effciency of persuasive space graphics (PSG) in motivating UK primary school children’s hand hygiene. International Journal of Environmental Research and Public Health [Internet]. 2020;17. Available from: <https://www.embase.com/search/results?subaction=viewrecord&id=L2004200674&from=export>

168. Saboori S, Greene LE, Moe CL, Freeman MC, Caruso BA, Akoko D, et al. Impact of regular soap provision to primary schools on hand washing and E. coli hand contamination among pupils in Nyanza Province, Kenya: a cluster-randomized trial. Am J Trop Med Hyg. 2013;89:698–708.

******Caruso BA, Freeman MC, Garn JV, Dreibelbis R, Saboori S, Muga R, et al. Assessing the impact of a school-based latrine cleaning and handwashing program on pupil absence in Nyanza Province, Kenya: a cluster-randomized trial. Trop Med Int Health. 2014;19:1185–97.

******Saboori S, Mwaki A, Rheingans RD. Is soapy water a viable solution for handwashing in schools? Waterlines. 2010;29:329–36.

169. Samreen K, Hiba A, Sundus I, Baig-Ansari N. Impact of hand hygiene intervention on hand washing ability of school-aged children. Journal of Family Medicine and Primary Care. 2021;10:642–7.

170. Sanders AM, Dixon R, Stuck L, Kelly M, Woods G, Muheki EM, et al. Evaluation of facial cleanliness and environmental improvement activities: Lessons learned from Malawi, Tanzania, and Uganda. PLoS Negl Trop Dis. 2021;15:e0009962.

171. Sangalang S, Borgemeister C, Kistemann T, Ottong Z, Lemence A, Medina S, et al. Schoolchildren’s hygiene-related health literacy and handwashing practices: results of a cluster-randomized controlled trial in Manila, Philippines. Tropical Medicine & International Health. 2021;26:188–9.

******Sangalang SO, Lemence ALG, Ottong ZJ, Valencia JC, Olaguera M, Canja RJF, et al. School water, sanitation, and hygiene (WaSH) intervention to improve malnutrition, dehydration, health literacy, and handwashing: a cluster-randomised controlled trial in Metro Manila, Philippines. BMC Public Health. 2022;22:2034.

172. Schroeder M, Yang L, Eifert J, Boyer R, Chase M, Nieto-Montenegro S. Evaluation of how different signs affect poultry processing employees’ hand washing practices. Food Control. 2016;68:1–6.

173. Scott BE, Schmidt WP, Aunger R, Garbrah-Aidoo N, Animashaun R. Marketing hygiene behaviours: the impact of different communication channels on reported handwashing behaviour of women in Ghana. Health Educ Res. 2008;23:392–401.

174. Shah SN, Shah D, Desai N, Shah SH, Bhowmick S. Analysis of change in knowledge, attitude, and practices about COVID-19 following and awareness session in rural population of Western India. Ind Psychiatry J. 2021;30:S35-s40.

175. Shahar S, Shahar HK, Muthiah SG, Mani KKC. Evaluating Health Education Module on Hand, Food, and Mouth Diseases Among Preschoolers in Malacca, Malaysia. Front Public Health. 2022;10:811782.

176. Sheth M, Obrah M. Diarrhea prevention through food safety education. Indian J Pediatr. 2004;71:879–82.

177. Simiyu S, Aseyo E, Anderson J, Cumming O, Baker KK, Dreibelbis R, et al. A Mixed Methods Process Evaluation of a Food Hygiene Intervention in Low-Income Informal Neighbourhoods of Kisumu, Kenya. Matern Child Health J [Internet]. 2022; Available from: <https://link.springer.com/content/pdf/10.1007/s10995-022-03548-6.pdf>

178. Simmerman JM, Suntarattiwong P, Levy J, Jarman RG, Kaewchana S, Gibbons RV, et al. Findings from a household randomized controlled trial of hand washing and face masks to reduce influenza transmission in Bangkok, Thailand. Influenza and other Respiratory Viruses. 2011;5:256‐267.

179. Sneed J, Phebus R, Duncan-Goldsmith D, Milke D, Sauer K, Roberts KR, et al. Consumer food handling practices lead to cross-contamination. Food Protection Trends. 2015;35:36–48.

180. Snow M, White GL Jr, Kim HS. Inexpensive and time-efficient hand hygiene interventions increase elementary school children’s hand hygiene rates. J Sch Health. 2008;78:230–3.

181. Soares K, Garcia-Diez J, Esteves A, Oliveira I, Saraiva C. Evaluation of food safety training on hygienic conditions in food establishments. Food Control. 2013;34:613–8.

182. Sobel DM, Stricker LW. Parent-child interaction during a home STEM activity and children’s handwashing behaviors. Front Psychol. 2022;13:992710.

183. Solehati T, Kosasih CE, Susilawati S, Lukman M, Paryati SPY. Effect of school community empowerment model towards handwashing implementation among elementary school students in Dayeuhkolot Subdistrict. Kesmas: National Public Health Journal. 2017;11:111–6.

184. Stebbins S, Stark JH, Vukotich CJ Jr. Compliance with a multilayered nonpharmaceutical intervention in an urban elementary school setting. J Public Health Manag Pract. 2010;16:316–24.

185. Stedman-Smith M, DuBois CL, Grey SF, Kingsbury DM, Shakya S, Scofield J, et al. Outcomes of a pilot hand hygiene randomized cluster trial to reduce communicable infections among US office-based employees. J Occup Environ Med. 2015;57:374–80.

186. Strohbehn CH, Paez P, Sneed J, Meyer J. Mitigating cross contamination in four retail foodservice sectors. Food Protection Trends. 2011;31:620–30.

187. Suen K, Cheung P. Effectiveness of “hand hygiene fun month” for kindergarten children: a pilot quasi-experimental study. International Journal of Environmental Research and Public Health. 2020;17.

188. Sutherland C, Reynaert E, Sindall RC, Riechmann ME, Magwaza F, Lienert J, et al. Innovation for improved hand hygiene: Field testing the Autarky handwashing station in collaboration with informal settlement residents in Durban, South Africa. Sci Total Environ. 2021;796:149024.

189. Takanashi K, Dao To Q, Nguyen Thi Le H, Nguyen Cong K, Yasuoka J, Jimba M. Long-term impact of community-based information, education and communication activities on food hygiene and food safety behaviors in Vietnam: a longitudinal study. PLoS ONE. 2013;8:e70654.

190. Taware S, Gawai P, Chatterjee A, Thakur H. Outcome of School-Based Intervention Program in Promoting Personal Hygiene in Primary School Children of Mumbai, India. Int Q Community Health Educ. 2018;39:31–8.

191. Thorseth AH, Heath T, Andualem S, Mare H, White S. An exploratory pilot study of the effect of modified hygiene kits on handwashing with soap among internally displaced persons in Ethiopia. Conflict and Health. 2021;15:(04 May 2021).

192. Tian X, Yan L, Zhao G, Wang L, Cheng Y, Lu Y, et al. Evaluation of a multi-layered health promotion approach in rural China. Glob Health Promot. 2019;26:14–24.

193. Tidwell JB, Gopalakrishnan A, Lovelady S, Sheth E, Unni A, Wright R, et al. Effect of Two Complementary Mass-Scale Media Interventions on Handwashing with Soap among Mothers. J Health Commun. 2019;24:203–15.

194. Tidwell JB, Gopalakrishnan A, Unni A, Sheth E, Daryanani A, Singh S, et al. Impact of a teacher-led school handwashing program on children’s handwashing with soap at school and home in Bihar, India. PLoS ONE. 2020;15:e0229655.

******Lewis HE, Greenland K, Curtis V, Schmidt WP. Effect of a school-based hygiene behavior change campaign on handwashing with soap in Bihar, India: cluster-randomized trial. Am J Trop Med Hyg [Internet]. 2018. Available from: <https://www.ajtmh.org/downloadpdf/journals/tpmd/99/4/article-p924.pdf>

195. Topan A, Alkan I, Ayyildiz TK, Mutlu B. Evaluation of the Effectiveness of Hand Hygiene Training Given to the Hearing-Impaired Children. International Journal of Caring Sciences. 2020;13:2073–81.

196. Tousman SA, Zeitz H, Bond D, Stewart D, Rackow R, Greer R, et al. A randomized controlled behavioral trial of a new adult asthma self-management program. Journal of Asthma and Allergy Educators. 2011;2:91–6.

197. Tousman S, Arnold D, Helland W, Roth R, Heshelman N, Castaneda O, et al. Evaluation of a hand washing program for 2nd-graders. J Sch Nurs. 2007;23:342–8.

198. Umair Q, Saeed A. Hand washing behavior change effect of community-based hygiene and sanitation intervention in low resource setting. Journal of Public Health. 2019;43:381–4.

199. Underwood CR, Broaddus ET, Kc S, Thapa RK. Community Theater Participation and Nutrition-Related Practices: Evidence from Nepal. J Health Commun. 2017;22:327–36.

******Choufani J, Jamaluddine Z, Cunningham K. A Multisectoral Nutrition Program in Nepal Improves Knowledge of Dietary Diversity, Sick Child Feeding, and Handwashing, but Not All Practices: a Program Impact Pathways Mediation Analysis. Curr Dev Nutr. 2020;4:nzz135.

200. Updegraff JA, Emanuel AS, Gallagher KM, Steinman CT. Framing flu prevention--an experimental field test of signs promoting hand hygiene during the 2009-2010 H1N1 pandemic. Health Psychol. 2011;30:295–9.

201. Vally H, McMichael C, Doherty C, Li X, Guevarra G, Tobias P. The Impact of a School-Based Water, Sanitation and Hygiene Intervention on Knowledge, Practices, and Diarrhoea Rates in the Philippines. Int J Environ Res Public Health. 2019;16.

202. Vaz Nery S, Traub RJ, McCarthy JS, Clarke NE, Amaral S, Llewellyn S, et al. WASH for WORMS: a Cluster-Randomized Controlled Trial of the Impact of a Community Integrated Water, Sanitation, and Hygiene and Deworming Intervention on Soil-Transmitted Helminth Infections. American Journal of Tropical Medicine and Hygiene. 2019;100:750‐761.

203. Violant-Holz V, Rodríguez-Silva C, Carol M, Rodríguez MJ. Impact of cocreation training capsules for preschool teachers on children’s healthy habits: a pilot study conducted in Barcelona, Spain. BMC Public Health. 2021;21:2089.

204. Waterkeyn J, Cairncross S. Creating demand for sanitation and hygiene through Community Health Clubs: a cost-effective intervention in two districts in Zimbabwe. Soc Sci Med. 2005;61:1958–70.

205. Watson J, Dreibelbis R, Aunger R, Deola C, King K, Long S, et al. Child’s play: Harnessing play and curiosity motives to improve child handwashing in a humanitarian setting. Int J Hyg Environ Health. 2019;222:177–82.

206. Weijers RJ, de Koning BB. Nudging to increase hand hygiene during the COVID-19 pandemic: A field experiment. Canadian Journal of Behavioural Science [Internet]. 2020; Available from: <https://www.scopus.com/inward/record.uri?eid=2-s2.0-85101666305&doi=10.1037%2fcbs0000245&partnerID=40&md5=fc42a7b5e63b57ff8aa0aacf288f5d9e>

207. White C, Kolble R, Carlson R, Lipson N, Dolan M, Ali Y, et al. The effect of hand hygiene on illness rate among students in university residence halls. Am J Infect Control. 2003;31:364–70.

******White C, Kolble R, Carlson R, Lipson N. The impact of a health campaign on hand hygiene and upper respiratory illness among college students living in residence halls. J Am Coll Health. 2005;53:175–81.

208. Wichaidit W, Biswas S, Begum F, Yeasmin F, Nizame FA, Najnin N, et al. Effectiveness of a large-scale handwashing promotion intervention on handwashing behaviour in Dhaka, Bangladesh. Tropical Medicine & International Health. 2019;24:972‐986.

209. Wichaidit W, Steinacher R, Okal JA, Whinnery J, Null C, Kordas K, et al. Effect of an equipment-behavior change intervention on handwashing behavior among primary school children in Kenya: the Povu Poa school pilot study. BMC Public Health. 2019;19:647.

210. Wilson JM, Chandler GN. Sustained improvements in hygiene behaviour amongst village women in Lombok, Indonesia. Trans R Soc Trop Med Hyg. 1993;87:615–6.

******Wilson JM, Chandler GN, Muslihatun, Jamiluddin. HAND-WASHING REDUCES DIARRHEA EPISODES - A STUDY IN LOMBOK, INDONESIA. Transactions of the Royal Society of Tropical Medicine and Hygiene. 1991;85:819–21.

211. Witt SD, Spencer HA. Using educational interventions to improve the handwashing habits of preschool children. Early Child Development and Care. 2004;174:461–71.

212. Wong SYW, Mahyudin NA, Ho JA, Abidin UFUZ. Evaluation of self-efficacy-based intervention: improving school food handlers selected food safety behavior. Food Protection Trends. 2022;42:8–21.

213. Wu S, Szeweiwang R, Huang Y, Wan TTH, Tung T, Wang B. Effect of hand hygiene intervention in community kindergartens: a quasi-experimental study. International Journal of Environmental Research and Public Health. 2022;19.

214. Yang C, Hu J, Tao M, Li Y, Chai Y, Ning Y, et al. Effectiveness of a multifaceted intervention on improving the hand-washing skills and behaviors of migrant workers in Beijing. Glob Health Promot. 2017;24:32–9.

215. Yardley L, Miller S, Schlotz W, Little P. Evaluation of a Web-based intervention to promote hand hygiene: exploratory randomized controlled trial. J Med Internet Res. 2011;13:e107.

216. Yeboah-Antwi K, MacLeod WB, Biemba G, Sijenyi P, Höhne A, Verstraete L, et al. Improving Sanitation and Hygiene through Community-Led Total Sanitation: The Zambian Experience. Am J Trop Med Hyg. 2019;100:1005–12.

217. York VK, Brannon LA, Shanklin CW, Robert KR, Howells AD, Barrett EB. Foodservice employees benefit from interventions targeting barriers to food safety. Journal of the American Dietetic Association. 2009;109:1576–81.

218. Younie S, Mitchell C, Bisson MJ, Crosby S, Kukona A, Laird K. Improving young children’s handwashing behaviour and understanding of germs: The impact of A Germ’s Journey educational resources in schools and public spaces. PLoS ONE. 2020;15:e0242134.

219. Yu H, Neal J, Dawson M, Madera JM. Implementation of behavior-based training can improve food service employees’ handwashing frequencies, duration, and effectiveness. Cornell Hospitality Quarterly. 2018;59:70–7.

220. Zemichael G, Ayenew A. Evidence of households’ water, sanitation, and hygiene (wash) performance improvement following a WASH education program in Rural Dembiya, Northwest Ethiopia. Environmental Health Insights. 2020;14.

221. Zhang C, Mosa AJ, Hayward AS, Matthews SA. Promoting clean hands among children in Uganda: a school-based intervention using “tippy-taps.” Public Health. 2013;127:586–9.

222. Zhang L, Qin X, Zeng J, Feng Y, Zhang N, Tan Y, et al. A kindergarten‐based, family‐involved intervention to improve children’s hand hygiene behavior: A cluster‐randomized controlled trial. Public Health Nursing. 2021;38:738–50.

223. Zomer TP, Erasmus V, Looman CW, Van Beeck EF, Tjon-A-Tsien A, Richardus JH, et al. Improving hand hygiene compliance in child daycare centres: a randomized controlled trial. Epidemiology and Infection. 2016;144:2552‐2560.
